# Effects of sampling methods on bee pollinators observed in *Cucurbita pepo*

**DOI:** 10.7717/peerj.20649

**Published:** 2026-02-02

**Authors:** Courtney Walls, Thomas Kuhar, T’ai Roulston, James Wilson

**Affiliations:** 1 Department of Entomology, Virginia Polytechnic Institute and State University (Virginia Tech), Blacksburg, VA, United States of America; 2 Department of Environmental Sciences, University of Virginia, Charlottesville, VA, United States of America; 3 Blandy Experimental Farm, University of Virginia, Boyce, VA, United States of America

**Keywords:** Pollinator, Bowl trap, Vacuum sample, Visual observations, Cucurbits, *Cucurbita pepo*, Pollinators

## Abstract

This study was performed to compare catch abundance of three commonly used bee sampling methods within pumpkin and squash (*Cucurbita pepo*) fields. The three methods of sampling included visual sampling, vacuum sampling, and bowl trapping. *Cucurbita pepo* require bee pollinators to move pollen from male flower to female flower for fruit to set. In *Cucurbita pepo* the three major expected bee types that should be observed are squash bees *Xenoglossa pruinosa* Say (formerly *Eucera* (*Peponapis*)* pruinosa*) [Hymenoptera: Apidae], bumble bees (*Bombus* spp.) [Hymenoptera: Apidae], and honey bees (*Apis mellifera* L. [Hymenoptera: Apidae]). Knowing what bees are present in a grower’s field could help them to make field management decisions and potentially reduce input costs, thus we chose methods where collected specimens could be submitted to experts for identification. We used visual observations as our control as we could confirm these bees were contacting flower reproductive segments, and this method is widely adopted in literature. Through this study, a total of 2,502 bees were observed, of which 82% were squash bees, 3.4% were honey bees and 2.4% were bumble bees. Bowl sampling differed significantly from visual observations for all three major bee types, having significantly fewer catches. Vacuum sampling recovered no bumble bees. Vacuum sampling was not significantly different from visual observation for honey bees but caught fewer squash bees relative to visual sampling. This study also looked at other collected morphotaxa of bees, particularly in bowl trapping, however bees beyond the three taxa presented above had limited presence in visual observations, and as such were not considered to play a major role in the pollination of these plants. This study indicates that visual observation of flowers provides a more reliable estimate of active pollinators in *C. pepo* fields than the two other bee sampling methods, vacuum sampling and bowl trapping, and they should likely not be deployed by growers to gain a representative sample of active pollinators in the field.

## Introduction

Pumpkins are an important seasonal crop in the United States, valued at $235 million annually (USDA National Agricultural Statistics Service, NASS, 2024). Pumpkins, along with summer squash and zucchini, are cultivars of the species *Cucurbita pepo* L. *Cucurbita pepo* cultivars are monoecious, having both staminate (male) and pistillate (female) flowers on the same plant. Most cucurbits require insect pollinators, in this case bees, to move pollen from staminate to pistillate flower for fruit to set (Nepi & Pacini, 1993). Flowers of *C. pepo* open only once, beginning at dawn (∼0500 h) and often closing by 1200 h, approximately a 7-hour window (Nepi & Pacini, 1993).

The major bee pollinators previously reported in Mid-Atlantic cucurbit systems are squash bees *Xenoglossa pruinosa* Say (formerly *Eucera* (*Peponapis*) *pruinosa*) [Hymenoptera: Apidae] (Freitas et al., 2023), bumble bees (*Bombus* spp.) [Hymenoptera: Apidae], and honey bees (*Apis mellifera* L. [Hymenoptera: Apidae]) (Julier & Roulston, 2009; Artz & Nault, 2011; Adamson et al., 2012; McGrady, Troyer & Fleischer, 2020). Honey bees and squash bees require up to 16 visits to pollinate a female *C. pepo* flower, while bumble bees require half as many visits (McGrady, Troyer & Fleischer, 2020). Honey bees and bumble bees are eusocial bees living in colonies where there is one queen and a force of workers to help provide for the colony’s needs. In contrast, the squash bee is a solitary bee where the female foundress is the sole caretaker of the nest (Michener, 2007; Amsalem et al., 2015; Pernal, 2021). In the United States, federal regulations provide guidelines for bee species that are actively managed (contracted colonies) for pollination services (EPA, 2016). Honey bees and some bumble bee species fall under the category of managed bees and so are addressed in state managed pollinator protection plans, whereas squash bees are not. The lack of recommendations for non-managed bees should be concerning as over 50% of agriculturally significant bees are native ground nesting bees (Rondeau, Willis Chan & Pindar, 2022) and the value of native pollinators in pumpkin production alone is estimated to be $101 million (Khalifa et al., 2021). Knowing what bees are present and knowledge of where they are nesting could help inform management decisions for the benefit of the growers, especially in pollination-dependent systems such as *C. pepo*. 

Currently, *C. pepo* producers are adding managed bees to help ensure pollination demands are met. Recommendations for stocking rates of honey bees can be anywhere from one to three hives per acre (McGregor, 1976; Ullmann et al., 2017) which can average between $40 and $70 per hive in Virginia (VDACS, 2024). Growers can also place colonies of *Bombus impatiens* Cresson [Hymenoptera: Apidae]) in cucurbit fields to help ensure crop pollination (Brochu et al., 2020; Artz & Nault, 2011). A package of bumble bees can cost from $178 to $290 per colony (Arbico Organics, 2025). Native ground nesting bees add an additional challenge to growers in that they are not portable or manageable, like the aforementioned eusocial bees. To better support the beneficial native pollinator populations growers must use practices to encourage their presence, such as rotating fields less than 0.5 km away from the prior year’s cucurbit field (Brochu et al., 2020). However, squash bees are biologically predisposed to finding cucurbit fields, as they are obligate feeders on *Cucurbita* pollen, often nesting on the edge or within cucurbit fields. Thus, if the grower adopts squash bee-friendly practices they could potentially positively influence the native pollinator presence, ideally without increasing costs.

While native bees often play a large role in the pollination of cucurbits, most growers are unable to successfully differentiate between types of bees. With insight into what pollinators are present in a given system, growers could then potentially help encourage pollinator populations in the field without the added cost of managed bees. One way for growers to be able to better know, and take advantage of, the abundance and diversity of bees in their field, would be to submit a sample of bees to an expert. Experts could help them discern what species of bees are present in the fields. The identification of these species could lend itself to identification of the bee’s nesting locations, which in turn could encourage best field management practices leading to conservation of nesting habitat. If the predominate univoltine species, either native bumble bees or squash bees, are not present in samples from the field, then short term pollination contracts could be established to maintain yield. While short term solutions like pollination contracts could support same season yield demands, adopting long term best pollinator management practices could help encourage establishment of pollinators within cropping systems and better support long term pollination demands. 

To gain a better understanding of the composition of bee communities within a location or cropping system, there must first be a standard that growers can use to observe bees actively pollinating a field. Sampling methods for bees in a community often rely on an abundance of traps to collect specimens, employing both active and passive trapping methods (Gibbs et al., 2017; Prendergast et al., 2020). Active sampling includes methods where an observer or collector is actively in the field sampling bees, while a passive method allows for an observer to set out the traps and return some period later to retrieve specimens (Droege, 2015). Most passive sampling methods are lethal to the bees captured by the trapping system. Examples of commonly used passive sampling methods for bees include bowl traps, blue vane traps, camera trapping, and Malaise traps. Some authors raise concerns about bowl sampling, as the bowls may be biased in catching particular bee types, mainly smaller sized bees, predominantly those in the Halictidae family (Cane, Minckley & Kervin, 2000; Gill & O’Neal, 2015; Bell, Tronstad & Hotaling, 2023). Additionally, bees caught in the trap may not be from that particular pollination system but rather could be by-catch if the bees were attracted to the trap while flying over the system being monitored and would increase handling cost of samples without increasing understanding of the pollination system (Gill & O’Neal, 2015). Camera trapping would allow for the confirmation of bees with the reproductive parts of flowers, however require technology that can be seen as an expensive barrier to growers. Active sampling methods can include a variety of techniques with some of the more popular options including visual observation, sweep netting, and vacuum sampling. Visual observation allows for the most accurate representation of contact with the reproductive parts of the flower, as the observer is directly confirming such contact. However, identification of some bee species with the eye alone can be challenging, so this may require representative samples to be collected as well (McGrady, Troyer & Fleischer, 2020; Packer & Darla-West, 2021). Often, it is a combination of trapping methods that are used and compared to assess the abundance and diversity of bees in a cropping system (Gill & O’Neal, 2015; Rhoades et al., 2017; Prendergast et al., 2020). Bowl traps are widely used and relatively inexpensive (Bell, Tronstad & Hotaling, 2023; Klaus et al., 2024) and in this study served as a representative of a commonly used passive sampling method. Bowl trapping has also been used in Virginia cucurbit systems to explore pollinator presence previously (Adamson et al., 2012). Sweep netting or targeted netting is the most commonly used active method of sampling (Bell, Tronstad & Hotaling, 2023) however in the *C. pepo* system plants often have vegetative growth over the flowers which could lead to excessive plant injury during the sampling effort, likely rendering sweep netting a non-viable option for this system. Thus, for this study, vacuum sampling was adapted as the active approach to provide targeted selection over the flowers and limit plant damage. Growers are typically not trained in bee identification, so methods like vacuum sampling and bowl trapping would allow for submission of collected bee samples by the grower to an expert to identify what bees were present in their field. Submitting samples for identification could eliminate potential misidentification of species collected by growers or citizen scientist (Klaus et al., 2024) as identification to species would be precise.

The objective of this study is to compare visual observation of pollinators present in cucurbit fields with vacuum sampling and bowl trapping as alternative methods for pollinator detection in cucurbit fields in Virginia. Ultimately, the most effective sampling method is one that could be easily executed by growers to determine the bee community composition in their fields to help minimize growing input costs that may be incurred with supplemental pollination contracts. For this study, trapping methods were implemented that would be easily accessible to growers and inexpensive. Multiple studies that have looked at pollinators in cucurbit systems have employed visual observation methods previously (Shuler, Roulston & Farris, 2005; Adamson et al., 2012; McGrady, Troyer & Fleischer, 2020; Willis Chan & Raine, 2021), and thus, visual observations will serve as a baseline to see if the encounter rate of bees is comparable between these three sampling methods.

## Materials and Methods

### Sampling methods

**Figure 1:**
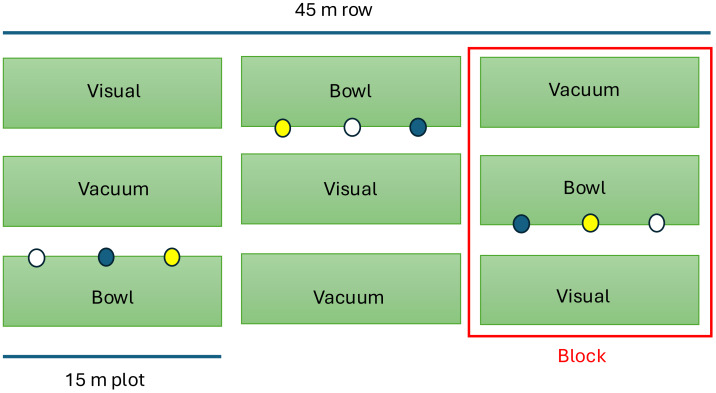
Sample zone plot design. Field arrangement of Latin square design with three trapping methods. Methods of visual sampling, vacuum sampling, and bowl sampling were implemented. The blue, yellow, and white circles indicate bowl placement and rotation of bowl color, which were interior to the plots.

The three methods used to observe the pollinator community present in the fields included visual observations, vacuum sampling, and bee bowl traps. Pumpkin fields were observed five times in two years (3 times in 2020 and 2 times in 2021) and the squash field two times (2020) during their respective peak bloom times for a total of seven observation days. A pair of sampling zones were set up in pumpkin plots and one sampling zone in summer squash (due to limitations of field size) totaling 12 observation plots. Sampling zones were comprised of three 45 m rows that were divided into 15 m blocks, which contained three plots. Each plot was 15 m long and with sampling methods organized as treatments in parallel rows which were assigned in a Latin square design (Fig. 1). Use of Latin square design in this cropping system allowed for control of flower density as an impacting factor of catch based on floral diversity, as previously reported in multiple studies that flower density can impact the number of bees observed by trapping method (Wilson, Griswold & Messinger, 2008; Lezzeri et al., 2024; Moldoveanu et al., 2025). All plots had similar number of plants based on plant spacing at planting. Fields were visited by trained observers on days where the weather was favorable for bee activity, characterized by temperatures above 17.8°C (64°F) (Rogers et al., 2023) and low wind speed <0.28 m/s (Adamson et al., 2012).

### Study sites

Survey locations were large (11.33-ha and 7-ha) commercial pumpkin fields located in Riner, VA (37.051077, −80.424509). The commercial pumpkin fields were managed with a three-year rotation using pumpkin, soybean, and corn so that each area was not planted in the same crop in consecutive years. Field crop rotations were typically adjacent to and within 20 m of the previous year’s cucurbit planting. Pumpkin fields in this study used a no-till winter rye cover crop that was rolled prior to planting, and pumpkins were seed drilled two inches in the ground at seeding a width of 1.2 m (4ft) plant spacing within a row and 2.4 m (8ft) between row centers. 25 honey bee hives were located 29 m from the field edge in 2020 and 4 m in 2021.

The summer squash small plot (0.1 ha) of yellow squash located at Kentland Farm in Whitethorne, VA (37.200905, −80.566607) was planted on raised beds covered with white plastic mulch with drip irrigation. Squash plants were spaced approximately 0.6 m apart within rows and beds were on 2-m centers. This study was performed during the peak of bloom of the cucurbit plants in (July/August 2020 and July 2021). Peak bloom was determined to be two weeks after the first presence of male flowers was observed.

### Morphotaxa classifications

For all sampling methods, observed bees were classified into nine morphotaxa: honey bee, bumble bee, squash bee, large black bee, small black bee, large striped bee, small striped bee, green bee, and other bee following McGrady, Troyer & Fleischer (2020). In-field collected bees were labeled with one of the above morphotaxa and stored according to method of observation. Authors performed preliminary species identification. Sam Droege, United States Geological Survey, provided final species determinations for collected specimens. Voucher specimens of all representative samples were deposited in the Virginia Tech Entomology Collection (Table S1). These morphotaxa categories were used to eliminate species bias as it is assumed that regardless of species, the specimens encompassed within each morphotaxa category are contributing equally to pollination efforts.

### Visual observations

Visual observations were performed at 0700, 0900, and 1100 h (when flowers were still open). Although, flowers could close as early as 0800 hrs (Seaton & Kremer, 1939). Three observers (with prior experience sampling bees using morphotaxa designations) worked in unison in individual 1-m sections of the same plot, allowing for each observer to record morphotaxa data from 5 m of each 15 m plot over 3 min, 45 sec/m of plot; the three observers worked together to visually sample the entire plot by the end of the period (McGrady, Troyer & Fleischer, 2020). Total number of flowers and sex of observed flowers were recorded. Flower visitations were only recorded if they were in the eye line of the observer and within a 1 ×1 m quadrant within the plot being observed. Bees were not limited to plot area during observation, as such bees may have made multiple encounters to multiple flowers across sampling time, however efforts were made to avoid double counted by individual observers. Bees were only recorded if they came into direct contact with the reproductive organs of the flower (stamens or stigma, respectively) (McGrady, Troyer & Fleischer, 2020). Representative samples of bees from each morphotaxon observed were collected in vials, placed on ice and stored in the freezer until washing (Droege, 2015) took place. Bees were saved to be used for identification at the species level to serve as representative samples of bees observed in the field for each morphotaxon recorded.

### Vacuum sampling

Plots were vacuum sampled directly after visual sampling was completed in the adjacent block at 0700 and 0900 (Fig. 1). The operator of the vacuum approached flowers with the vacuum first to limit the disruption of bees within a given flower. Every flower in the 15 m plot was vacuumed once and the collected bees were then placed on ice in the field and later moved to the freezer before being washed to remove pollen and pinned for identification to species level. Flower sex and count were recorded for each vacuum sampling. Vacuum sampling was performed with two vacuum types. The first was a modified leaf blower vacuum Homelite 150 MPH 400 CFM 26cc Gas Handheld Blower Vacuum (Homelite Corp., Anderson, SC) on the vacuum setting. A 0.6-liter Gatorade (PepsiCo Purchase, NY) bottle with the bottom of the bottle and cap removed was used to narrow the vacuum’s opening to direct more negative pressure over the flowers. A 18.93-liter paint strainer mesh bag was taped to the mouth of the bottle and the vacuum was turned on. Immediately after sampling the 15 m plot, the vacuum was turned off, and the paint strainer bag was closed and placed in a Ziploc bag (SC Johnson, Bay City, MI) to be set on ice. The leaf blower vacuum caused damage to female flowers, so the vacuum system was changed to a Bioquip heavy duty hand-held DC vacuum (Bioquip Products, Inc., Rancho Dominguez, CA* note, no longer in business) with a wire mesh extension at the end of the vacuum tube to allow flow of air inside the flower. All specimens were collected in the collection chamber and then placed in a gallon plastic storage bag to be placed on ice and then frozen prior to washing and pinning for identification to species level.

### Bee bowls

Bee bowls were made from 750 mL plastic food storage bowls (model number: 2115739 (clear)), Rubbermaid, Atlanta, GA), and painted with UV-bright yellow, Horizon fluorescent blue, or silica flat white paint (Blick Art Materials, Galesburg, IL, USA) following the methods of McCullough, Angelella & O’Rourke (2021). One bowl of each color was placed at 2.5 m, 7.5 m, and 12.5 m spacing in the 15 m designated plot in a rotating order of white, blue, and yellow (Droege et al., 2010; Gibbs et al., 2017; O’Connor et al., 2019; McCullough, 2020; Angelella, McCullough & O’Rourke, 2021; McCullough, Angelella & O’Rourke, 2021). Bowls were placed on the side of the plot that was interior to the Latin square and the neighboring plots in the row (Fig. 1). Each bowl was laid flat on the ground and filled halfway with a soapy water solution prepared with Dawn original dishwashing soap (Proctor & Gamble, Jackson MI) and enough soap was used to produce suds (∼0.2 ml), but not to leave an odor (adapted from Droege et al., 2010; McCullough, Angelella & O’Rourke, 2021). Bowls were set out at 1100 hrs. and collected 48 h later (one sample was collected 72 h later due to herbicide spray re-entry restrictions). Specimens were collected from the bee bowls by straining the collected specimens through a wire mesh strainer in the field and specimens were then placed in 70% ethanol until they were washed, dried, pinned and later identified to species level.

### Collected specimens preparation

All collected specimens were washed according to the following procedures following Droege (2015). Bees were swirled in soapy water solution made with Dawn Dishwashing Liquid (Proctor and Gamble, MO) for 2 minutes in a 500 mL Erlenmeyer flask and then strained and rinsed. After the wash, they were dipped in 70% ethanol and carefully patted dry using a cloth towel. Specimens were then placed back into the 500 mL Erlenmeyer flask with two Bounty napkins (Proctor and Gamble, Mehoopany, PA) and dried with pressurized air for 2 min or until hairs were visibly dry. Specimens were pinned, grouped by field-assigned morphotaxa, and labeled accordingly to then be identified to species.

### Statistical analysis

All analyses were conducted in R studio 2021.09.0 (R Core Team, 2000) for Mac. Histograms and Q-Q plots were developed for each morphotaxon to check for distribution (Chambers, 1983; R Core Team, 2000). Data were zero inflated for every morphotaxon and two morphotaxa were excluded from analysis due to very low observance rates (zero and seven total across trap types, see Table 1, large striped bee and other bee, respectively). For each of the seven remaining morphotaxa, the effect of trap type on bee count was analyzed using a zero inflated General Linear Mixed Model (GLMM) in the glmmTMB package, with fixed effects of trap type (Brooks et al., 2017). Random effects were set as date and location, where the variables were not confounded. Zero inflated intercept was set to one (ziformula ∼1) to account for zeros within data (Brooks et al., 2017). Distribution of data for each morphotaxon was determined to be negative binomial (nbinom2 or nbinom12) or Poisson distribution using the DHARMa package to run uniformity, distribution, zero- inflation, and homogeneity of variance tests (Hartig, 2022) to best fit distribution. Likelihood ratio tests (LRT) were performed using the anova function comparing GLMMs with and without the fixed effect of trap type to produce a chi-square value, degrees of freedom, and *p* value to indicate if trap type was significant (Brooks et al., 2017).

**Table 1:** Total bees collected by trapping method. Captured numbers of nine major bee morphotaxa by trap type, *n* = 2,502, representing overall trapping differences (* indicates data having catch numbers too low to be processed using General Linear Mixed Model methods).

Morphotaxa	Bowl	Vacuum	Visual
Honey Bee	6	19	62
Bumble Bee	6	0	55
Squash Bee	350	241	1,455
Small Black Bee	160	10	3
Large Black Bee	31	0	3
Small Striped Bee	39	0	4
Large Striped Bee*	6	0	1
Green Bee	43	1	7
Other Bee*	0	0	0
Total Bees by Trap	641	271	1,590
Total Bees			2,502

*Post Hoc* analysis of main effects was carried out using estimated marginal means (emmeans package) (Lenth, 2017) and sidak comparison with alpha 0.05 pairwise function to produce letters of significance using the compact letter display function (multcomp and multcompView packages) (Graves, Piepho & Selzer, 2015). For compact letter display, type was set to response to give a rate that was back transformed to count based numbers. Violin plots for data were produced using ggplot2 (Wickham, 2016), where wider width of plot illustrates the most frequently observed count values.

## Results

In total, 2,502 bees were recorded in this study (Table 1), including 665 collected as voucher specimens (Table S1). Through this study, three bee families, Andrenidae, Apidae, and Halictidae, were observed containing 12 genera and 30 species (Table S1). Squash bees comprised 82% of bee counts, while honey bee and bumble bees comprised 3% and 2%, respectively. Squash bees were the most observed taxon across all three sampling techniques including: 55% of all bowl samples, 89% of vacuum samples, and 92% of the visual sampling. Small black bees accounted for 7% of total bee counts but comprised 25% of total bowl samples. Large striped bees and other bees were rarely observed (seven total) and excluded from analysis.

**Table 2:** Honey bee general linear mixed model statistical results. Results of zero-inflated Poisson GLMM for honey bee counts with fixed effects of trap type. Random effect was set to date, with random intercepts and zero-inflation modeled with constant probability. (* indicates a *p* value of <0.05). (Bold text), indicates a statical significance based on a *p* value of < 0.05.

Fixed effects	Estimate	Standard error	Z-value	*P* value
Visual	−0.8991	1.1036	−0.815	0.415225
Bowl	−2.0877	0.5412	−3.858	0.000115*
Vacuum	−0.8339	0.29	−2.876	0.004031*
Intercept (Zero Inflated)	−0.582	0.4641	−1.254	0.21
Random Effects				
Date	4.447	2.109		
Model Fit				
AIC	205.1			

### Honey bees

**Figure 2:**
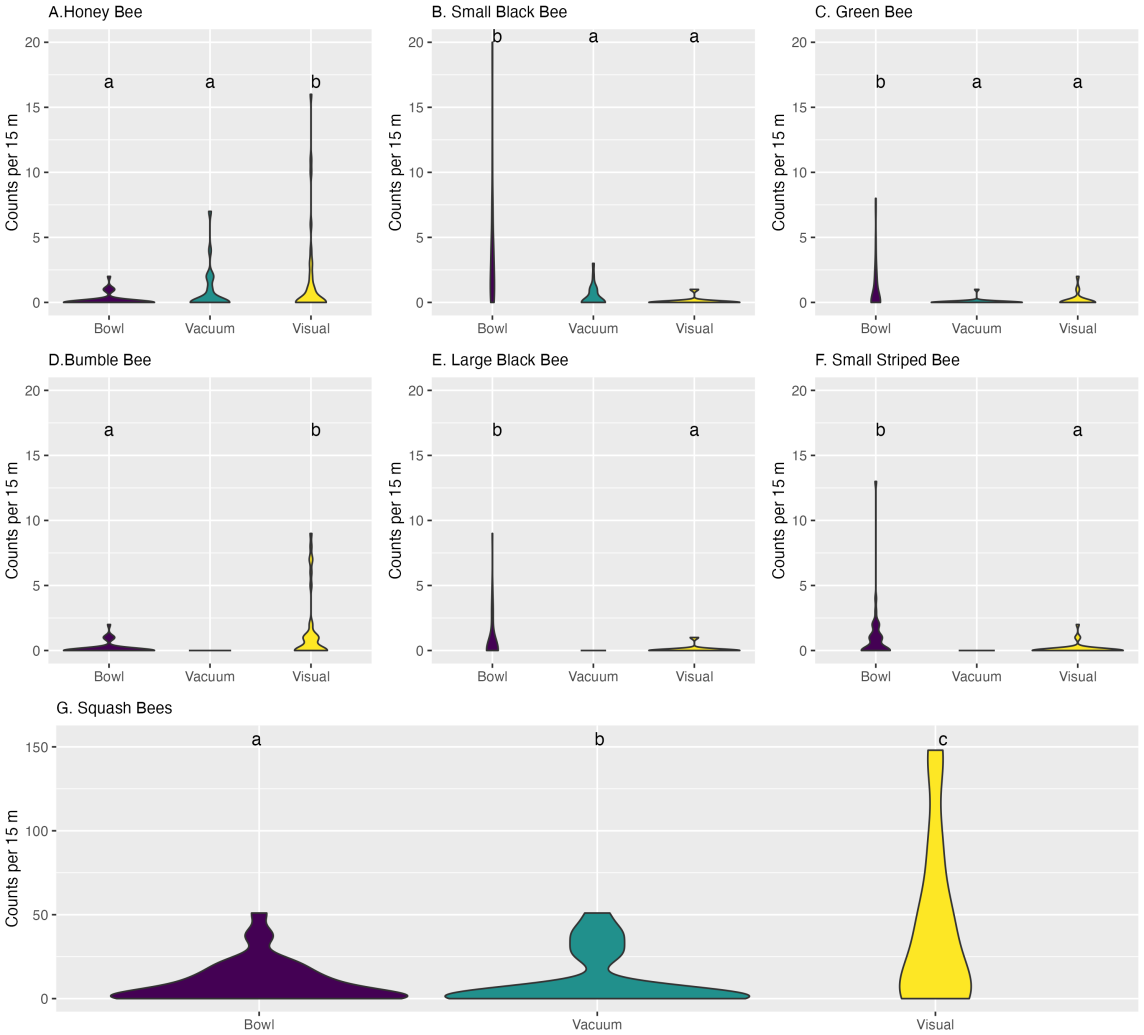
Violin plots of bees counts by plot of observation by Morphotaxon. Each violin plot, which represents counts of bees by observation event, is on the y axis and the x axis is trap type for bowl trapping, vacuum sampling, or visual observations. Violin plots have greater width at count values with higher frequencies of observation. For each morphotaxon, groups sharing the same cld letter are not significantly different (*p* < 0.05) based on zero-inflated GLMM run by each morphotaxon group.

For honey bees trap type had a large impact on the number of bees observed (GLMM LRT, X^2^ = 23.739, *df* = 2, *p*-value = 1.154e−05). When compared to the standard of visual sampling bowl, samples had significantly fewer samples (Table 2). Comparing the rates of the estimated marginal means (EMM) we see that there was not a large observation of honey bees. However, there is a significant difference between bowl sampling and vacuum and visual observation. With visual observations having 0.41 (standard error (SE) ± 0.45) bees per 15 m plot and vacuum with 0.18 (SE ± 0.20) bees per 15 m plot, while bowl sampling had a rate of 0.05 (SE ± 0.06) bees per 15 m (Table 3). *Post hoc* analysis separated visual and vacuum with compact letters (Fig. 2A). Zeros in these data were not significant (*p* = 0.21) (Table 2).

**Table 3:** Honey bee estimated marginal means table. Table includes statistical outputs from estimated marginal means for honey bees where Rate indicates estimated bee collection rate per 15 m, based on zero-inflated GLMM. Rates were back transformed from the log scale. Sidak groups of different letters indicate significant differences between trap type (*a* = 0.05, Sidak-adjusted).

TRAP	Rate	SE	df	asymp.LCL	asymp.UCL	Sidak group
Bowl	0.0504	0.0612	Inf	0.00279	0.913	a
Vacuum	0.1767	0.2011	Inf	0.01168	2.675	a
Visual	0.4069	0.4491	Inf	0.02918	5.675	b

### Bumble bees

No bumble bees were recovered with vacuum sampling (Fig. 2D). The model indicated that trap played a large role in the count of bumble bees observed (GLMM LRT, X^2^ = 36.26, *df* = 1, *p*-value = 1.727e−09). The zeros in these data did not have a large impact on the model with *p* = 0.99 (Table 4). There were significant differences observed between visual sampling (0.44 ± 0.29 bees/15 m) and bowl trapping (0.05 ± 0.4 bees/15 m), with bowl having significantly fewer than visual (Table 5), with *post hoc* separation of letters of “a” for bowl trapping and “b” for visual sampling (Fig. 2D).

**Table 4:** Bumble bee general linear mixed model statistical results. Results of zero-inflated GLMM with poisson distribution for bumble bee counts with fixed effects of trap type. Random effects were set to date, with random intercepts and zero inflation modeled with constant probability. (* indicates a *p* value of <0.05). Vacuum is not included as no bees were recovered from this method. (Bold text), indicates a statical significance based on a *p* value of < 0.05.

Fixed effects	Estimate	Standard error	Z-value	*P* value
Visual	−0.8239	0.6514	−1.265	0.206
Bowl	−2.1878	0.4344	−5.037	4.74e−07*
Intercept (Zero Inflated)	−21.4	14,640.3	−0.001	0.999
Random effects				
Date	2.213	1.488		
Model fit				
AIC	112.8			

**Table 5:** Bumble bee estimated marginal means table. Statistical outputs from estimated marginal means for bumble bees where rate indicates estimated bee collection rate per 15 m, based on zero inflated GLMM. Rates were back transformed from the log scale. Sidak groups of different letters indicate significant differences between trap type (*a* = 0.05, Sidak-adjusted).

TRAP	Rate	SE	df	asymp.LCL	asymp.UCL	Group
Bowl	0.0492	0.0368	Inf	0.00925	0.262	a
Visual	0.4387	0.2858	Inf	0.1022	1.883	b

### Squash bees

The effect of trap method was highly significant on squash bee capture (GLMM LRT, X^2^ = 24.274, *df* = 2, *p*-value = 5.357e−06). There was a small number of zeros in the data indicated by the zero inflated term *p* = 0.00267 (Table 6). Estimated marginal means showed a significance between all three trap types, with visual observations observing 10.78 (±9.66) squash bees per 15 m. Vacuum had the next highest rate with 6.48 (±5.89) bees per 15 m, while bowl had the fewest with 3.25 (±2.93) (Table 7). Using *post hoc* analysis all of these sampling methods were separated into their own group of “a”, “b”, and “c” for bowl, vacuum, and visual, respectively (Fig. 2G).

**Table 6:** Squash bee general linear mixed model statistical results. Results of zero-inflated GLMM with negative binomial distribution for squash bee counts with fixed effects of trap type. Random effects were set to date and location; both were set with random intercepts and zero-inflation modeled with constant probability. (* indicates a *p* value of <0.05. (Bold text), indicates a statical significance based on a *p* value of < 0.05.

Fixed effects	Estimate	Standard error	Z-value	*P* value
Visual	2.3775	0.8961	2.653	0.00797*
Bowl	−1.1991	0.1626	−7.375	1.64e−13*
Vacuum	−0.5081	0.1927	−2.636	0.00838*
Intercept (Zero Inflated)	−4.294	1.43	−3.004	0.00267*
Random effects				
Location	0.8057	0.8976		
Date	3.1179	1.7658		
Model fit				
AIC	590.1			

**Table 7:** Squash bee estimated marginal means table. Statistical outputs from estimated marginal means for squash bees where rate indicates estimated bee collection rate per 15 m, based on zero inflated GLMM. Rates were back transformed from the log scale. Sidak groups of different letters indicate significant differences between trap type (*a* = 0.05, Sidak-adjusted).

TRAP	Rate	SE	df	asymp.LCL	asymp.UCL	Sidak group
Bowl	3.25	2.93	Inf	0.377	28	a
Vacuum	6.48	5.89	Inf	0.742	56.6	b
Visual	10.78	9.66	Inf	1.269	91.6	c

**Table 8:** General linear mixed model statistical results for all other bee morphotaxa. (a) Results of zero-inflated GLMM negative binomial distribution for small black bee counts with fixed effects of trap type. Random effects were set to date and location with random intercepts and zero-inflation modeled with constant probability. (* indicates a *p* value of <0.05) (b). Results of zero-inflated GLMM poisson distribution for large black bee counts with fixed effects of trap type. Random effects was set as date, set with random intercepts and zero-inflation modeled with constant probability. (* indicates a *p* value of <0.05) Vacuum was not included as no counts were recorded. (c) Results of zero-inflated GLMM poisson distribution for small striped bee counts with fixed effects of trap type. Random effects were set to date and location with random intercepts and zero-inflation modeled with constant probability. (* indicates a *p* value of <0.05). Vacuum was not included as no counts were recorded. (d) Results of zero-inflated GLMM poisson distribution for green bee counts with fixed effects of trap type. Random effects were set to date and location with random intercepts and zero-inflation modeled with constant probability. (* indicates a *p* value of <0.05). (Bold text), indicates a statical significance based on a *p* value of < 0.05.

	Estimate	Standard error	Z-value	*P* value
Small Black Bee				
Fixed effects				
Visual	−2.4132	0.827	−2.918	**0**.00352*
Bowl	4.4435	0.7676	5.789	7.09e−09*
Vacuum	1.4199	0.7454	1.905	0.05681
Intercept (Zero Inflated)	−8.897	1003.434	−0.009	0.993
Random effects				
Location	0.54544	0.73854		
Date	0.006215	0.07884		
Model fit				
AIC	263.3			
Large Black Bee				
Fixed effects				
Visual	−1.4343	0.8075	−1.776	0.07569
Bowl	2.4018	0.7708	3.116	**0**.00183*
Intercept (Zero Inflated)	0.9127	0.4088	2.233	**0**.0256*
Random effects				
Date	0.3876	0.6225		
Model fit				
AIC	106.4			
Small Striped Bee				
Fixed effects				
Visual	−2.3574	0.7346	−3.209	**0**.00133*
Bowl	2.3866	0.5812	4.106	4.02e−05*
Intercept (Zero Inflated)	−1.2591	0.8304	−1.516	0.129
Random effects				
Location	6.28e−09	7.92e−05		
Date	1.24	1.11		
Model fit				
AIC	134.9			
Green Bee				
Fixed Effects				
Visual	−1.1906	0.7745	−1.537	.124
Bowl	2.2737	0.8299	2.74	**0**.00615*
Vacuum	−1.6503	1.1263	−1.465	0.14287
Intercept (Zero Inflated)	0.02292	0.57946	0.04	0.968
Random effects				
Location	.295	.543		
Date	0.3479	.590		
Model fit				
AIC	166.8			

### Other morphotaxa

For all other bee morphotaxa, bowl trapping had significantly more observed bees than visual or visual and vacuum sampling. Small black bee was the most observed for “other morphotaxon”, and trap method played a large role in the bees observed (GLMM LRT, X^2^ = 64.531, *df* = 2, *p*-value = 9.711e −15) for this morphotaxa as well. When compared to visual observation, bowl sampling was significantly different while vacuum sampling was not (Table 8). The estimated marginal means for small black bee bowl trapping had a rate of 6.27 (±3.66) while visual and vacuum were only 0.19 (±0.14) and 0.67 (±0.41), separating bowl from visual and vacuum (Fig. 2B). Green bee showed similar results (Table 8), with a significant effect of trap on bees observed (GLMM LRT, X^2^ = 22.618, *df* = 2, *p*-value = 1.22e−05). There were fewer green bees than small black bees in the estimated marginal means observed in bowl trapping; however, bowl trapping still had the greatest estimated marginal mean rate of 2.95 (±2.46) per 15 m (Table 9). *Post hoc* of green bees showed separation of groups with “a” assigned to visual and vacuum sampling and “b” assigned to bowl trapping (Fig. 2C). For large black bee and small striped bee, vacuum sampling was excluded from analysis as the count observed from this method was zero. Large black bee was significantly impacted by trap type in bees observed per 15 m (GLMM LRT, X^2^ = 7.6103, *df* = 1, *p*-value = 0.0058). Visual observation (EMM 0.24 ± 0.19) had significantly smaller rate of bee observation than bowl trapping observation (EMM 2.63 ± 1.16) (Table 9). For small striped bees, bowl trapping (EMM 1.03 ± 0.51) also had a higher rate of observation of bees per 15 m than visual observation (EMM 0.09 ± 0.07) (Table 9). Sampling methods for small striped bee proved to be significant in rate of observation of bees (GLMM LRT, X^2^ = 17.751, *df* = 1, *p*-value = 2.518e−05). *Post hoc* analysis for both large black bee (Fig. 2E) and small striped bee (Fig. 2F) separated visual observation and bowl trapping into “b” and “a” groups.

**Table 9:** Estimated marginal means table all other morphotaxa. Statistical outputs from estimated marginal means for small black bees, large black bees, small striped bees, and green bees where rate indicates estimated bee collection rate per 15 m, based on zero inflated GLMM for each of the respective morphotaxa. Rates were back transformed from the log scale. Sidak groups of different letters indicate significant differences between trap type (*a* = 0.05, Sidak-adjusted).

TRAP	Rate	SE	df	asymp.LCL	asymp.UCL	Sidak group
Small Black Bee						
Visual	0.193	0.144	Inf	0.0325	1.15	a
Vacuum	0.668	0.41	Inf	0.1544	2.89	a
Bowl	6.271	3.66	Inf	1.5559	25.27	b
Large Black Bee						
Visual	0.238	0.192	Inf	0.0392	1.45	a
Bowl	2.631	1.158	Inf	0.9831	7.04	b
Small Striped Bee						
Visual	0.0947	0.0695	Inf	0.0183	0.489	a
Bowl	1.0296	0.5108	Inf	0.3395	3.123	b
Green Bee						
Vacuum	0.0584	0.0765	Inf	0.00255	1.34	a
Visual	0.304	0.2355	Inf	0.04785	1.93	a
Bowl	2.9539	2.4643	Inf	0.40299	21.65	b

## Discussion

With the composition and abundance of pollinators changing rapidly worldwide, it is critical to understand the species acting as pollinators in agricultural systems, particularly in pollinator dependent cropping systems (Kluser & Peduzzi, 2007; van der Sluijs, 2020; Dicks et al., 2021; Vasiliev & Greenwood, 2021; LeBuhn & Luna, 2021; Rodger et al., 2021; Stout & Dicks, 2022). Cucurbits are a prime example of a pollinator-dependent cropping system, as they require pollinators to move pollen from the male flower to female flower for fruit to set (Willis Chan & Raine, 2023). The most commonly observed pollinators in pumpkins and squash (*C. pepo*) included honey bees, bumble bees, and squash bees (Shuler, Roulston & Farris, 2005; Adamson et al., 2012; McGrady, Troyer & Fleischer, 2020; Stoner, 2020; Rondeau, Willis Chan & Pindar, 2022; Willis Chan & Raine, 2023). Visual observations were used as our standard to compare the pollinators observed in this system as multiple studies prior have used them for sampling bees in cucurbit systems (Shuler, Roulston & Farris, 2005; Adamson et al., 2012; McGrady, Troyer & Fleischer, 2020; Willis Chan & Raine, 2021). Visual observations may not be conducive for growers if they have not been properly trained in bee identification. Identification can be important as the life cycles of the bees that could be working in their fields can vary significantly (Klaus et al., 2024) and current field management methods that protect eusocial bees may not help solitary bees that are just as likely to be key pollinators in cucurbit cropping systems. This study observed all three of the major pollinators within the cropping system; however, honey bees and bumble bees were significantly less abundant than squash bees as seen in Shuler, Roulston & Farris (2005) and Adamson et al. (2012) (both in Virginia), but contrary to the findings of McGrady, Troyer & Fleischer (2020) (in Pennsylvania) and Stoner (2020) (in Connecticut). This indicates that in Virginia, squash bees could play a critical role in pollination of *C. pepo*. Methods of trapping for all morphotaxa exhibited significant differences in count numbers between at least two trap methods indicating not all trapping methods accurately represent known pollinating species in this system.

Looking at the difference in the abundance of the three major pollinator morphotaxa, no bumble bees were captured in vacuum sampling, very few were recovered from bowl sampling, and significantly more bumble bees were observed in visual observations (Fig. 2D). The small number of bumble bees in bowls has been previously identified as an issue with this sampling method (Roulston, Smith & Brewster, 2007) with some studies arguing they are not suitable for sampling bumble bees at all (Tronstad, Bell & Crawford, 2022; Bell, Tronstad & Hotaling, 2023). Recovering no bumble bee specimens indicates vacuum sampling is not appropriate for this system and could miss an entire group of key pollinators for growers in this system. For honey bees (Fig. 2A), there were significantly fewer honey bees observed by bowl sampling than with visual observations but vacuum and visual observations were not significantly different when the pumpkin fields had honey bees on site for pollination services. While these pumpkin fields were under pollination contracts with 25 colonies on site, counts for honey bees were not greater than 10 bees per plot through both years (Fig. 2). Our small number of observed honey bees was not surprising, as multiple studies have shown that the addition of honey bees to a cucurbit field did not increase their presence as pollinators within the *Cucurbita* system (Shuler, Roulston & Farris, 2005; Petersen, Reiners & Nault, 2013; Brochu et al., 2020; Willis Chan & Raine, 2021). In fact, these estimates predict less than one honey bee per 15 m of field. Squash bees made up a majority of the bees observed in this study, 82%. Squash bees estimated rates were significantly different across all three sampling methods (Fig. 2G), with visual sampling having the highest recorded rate, followed by vacuum sampling and then bowl trapping. Results for the three main morphotaxa of cucurbit pollinators show that visual observation tallied more specimens than bowl trapping or vacuum sampling for every species indicating that these sampling methods estimate disproportionately pollinator presence and activity in cucurbit systems. Additionally, visual observations were only made if the bees came into contact with the reproductive parts of the flower and the other traps had the potential to collect bees that were not active pollinators of the system. These methods would not be suitable for deployment by growers for an assessment of field pollinator communities in cucurbit production systems.

We chose to use vacuum sampling equipment that was not specialized (a common leaf blower with reverse function) and could be accessible to growers. Ozanne (2005) state that vacuum sampling may not be suitable for large Hymenoptera; however, it could be practical for smaller hymenopterans. This could explain why we recovered no bumble bees using these methods, but very few small bees were recovered overall using this method. Our results show the vacuum sampling was most suitable for a medium sized bee, like squash or honey bee. In *Cucurbita* spp. cropping systems, plants often have vegetative growth over flowers which means sampling methods like sweep netting or vacuuming could lead to potential damage to flowers or plants (McCravy, 2018), here we aimed to minimize impacting plants in our sampling methods. In our study, we observed damage to the flowers and thus the fruiting bodies of the plants from the method of vacuum sampling. As such, vacuum sampling would be more damaging than useful as an effective pollinator sampling method in the *C. pepo* system, in addition our results show that this method is also likely underestimating the pollinators present in this system.

In this study, bowl sampling led to a large collection of bee types outside the major three morphotaxa. In fact, for all small bee types (smaller than the size of a honey bee >15 mm)—including small striped bee, small black bee, and green bee—there were significantly more bees observed in the bowl traps than in visual sampling (Fig. 2). When assessing pollinator abundance or diversity based on bowl data alone, results are heavily skewed towards that of small bee types, a trend observed in several other studies (Portman, Bruninga-Socolar & Cariveau, 2020; Tronstad, Bell & Crawford, 2022; Bell, Tronstad & Hotaling, 2023). Even with these large capture numbers however, other bee types are not often considered effective pollinators in the cucurbit systems despite being present in bee bowl samples. For example, it is estimated the *Lasioglossum* bees would take 123 trips to a female flower to pollinate *Cucurbita* (Pfister et al., 2017). *Lassioglossum* was included in our small black bee morphotaxa, which had a significant difference between sampling methods, with bowl trapping having the largest collection amount and visual observations and vacuum sampling having significantly fewer. Additionally, some arguments show that the bees captured in these bowls could be incidental by-catch as they were in the field either for weed bloom or accidental captures flying over the field and not contributing to crop pollination in the first place (Angelella, McCullough & O’Rourke, 2021; McCullough, Angelella & O’Rourke, 2021).

Some studies encourage the use of multiple sampling methods, for example using both net sampling and bowl trapping, in efforts to give an accurate description of species abundance and diversity within a system (Wilson, Griswold & Messinger, 2008; Moldoveanu et al., 2025), however for the purpose of this study we aimed to provide methods that were easy to implement. The methods included in this study were to save growers’ costs, not only including materials needed, but also in the cost of time. Additionally, the capture of bowl traps in this system did not lend to the collection of the bees considered major pollinators the *C. pepo* system (Shuler, Roulston & Farris, 2005; Adamson et al., 2012; McGrady, Troyer & Fleischer, 2020; Stoner, 2020; Rondeau, Willis Chan & Pindar, 2022; Willis Chan & Raine, 2023) which could be misleading to growers. In Virginia, pollinator protection and best management practice recommendations are focused on honey bees or other cavity nesting and managed bees (including some bumble bees) being contracted to pollinate (Ullmann et al., 2017; Reiter et al., 2022). Contract pollinator colonies rely on large work forces of generalist bees which can be moved in or out of fields they are contracted to pollinate. In contrast, squash bees are native ground nesting specialist bees that are obligatory feeders on the plant host genus *Cucurbita* (Hurd Jr, Linsley & Michelbacher, 1974) and they make nests in or on the edge of the cucurbit fields that must remain undisturbed between years. As such, squash bees have a higher risk of potential exposure to pesticides and can be directly impacted by field management techniques (Rondeau, Willis Chan & Pindar, 2022). For example, deep tillage practices cause a later emergence in squash bees in the following year (Ullmann, Meisner & Williams, 2016) and plastic mulching can have a negative impact on their ability to nest (Splawski et al., 2014). With the observed high abundance of squash bees in this study, it is critical for growers to have management practices that are easy to implement while being cost effective to help protect these ground nesting, native bees that are critical to Virgina cucurbit production.

## Conclusions

Overall, our study illustrates that bowl trapping and vacuum sampling likely underrepresent the pollinators present in the *C. pepo* systems of Virginia compared to visual observations. This is especially true in the case of the three major pollinator types of squash bee, bumble bee, and honey bee present in this cropping system. The sampling methods of bowl trapping and vacuum sampling, while quick and inexpensive, are not suitable replacements for visual sampling in *C. pepo* systems and would not be effective tools that could be deployed by growers to estimate pollinator presence in their field. However, it is still critical for growers to understand pollinator presence in their fields as these findings suggest that the native ground nesting bee, the squash bee, plays a large role in Virginia *C. pepo* production as 82% of the bees recovered in this study were squash bees. This study indicates that management practices that encourage ground nesting bees in these *C. pepo* should be developed in a prescriptive way and implemented, as currently the plan to mitigate risks to managed pollinators (VDACS, 2017) is the only available literature to growers in our state. Further work is needed to explore the impact that agricultural inputs and practices may already be having on these ground nesting native bees that appear to carry out the pollination needed in this pollination dependent system.

##  Supplemental Information

10.7717/peerj.20649/supp-1 Supplemental Information 1 Raw Data compiled by 15 meter plot

10.7717/peerj.20649/supp-2 Supplemental Information 2 The complete code and analysis (PDF)

10.7717/peerj.20649/supp-3 Supplemental Information 3 Collected Bee Specimens From Trapping Methods

10.7717/peerj.20649/supp-4 Supplemental Information 4 R code

## Additional Information and Declarations

### Data Availability

The following information was supplied regarding data availability: The raw data and code are available in the Supplemental Files.
