# Supplementary material for: Effects of sampling methods on bee pollinators observed in Cucurbita pepo
Source: PeerJ. 2026 Feb 2;14:e20649. doi: 10.7717/peerj.20649 (PMC12875216; doi:10.7717/peerj.20649)
Supplement: Supplemental Information 2 [file peerj-14-20649-s002.pdf]

Analyisis for Walls et al.

2025-08-08

Library

```
library(glmmTMB)
```

```
## Warning: package 'glmmTMB' was built under R version 4.3.3

## Warning in checkMatrixPackageVersion(): Package version inconsistency detected.
## TMB was built with Matrix version 1.6.0
## Current Matrix version is 1.6.1.1
## Please re-install 'TMB' from source using install.packages('TMB', type = 'source') or ask CRAN for a

## Warning in check_dep_version(dep_pkg = "TMB"): package version mismatch:
## glmmTMB was built with TMB package version 1.9.17
## Current TMB package version is 1.9.6
## Please re-install glmmTMB from source or restore original 'TMB' package (see '?reinstalling' for more
```

```
library(tidyr)
library(dplyr)
```

```
##
## Attaching package: 'dplyr'

## The following objects are masked from 'package:stats':
##
##     filter, lag

## The following objects are masked from 'package:base':
##
##     intersect, setdiff, setequal, union
```

```
library(emmeans)
library(DHARMa)
```

```
## Warning: package 'DHARMa' was built under R version 4.3.3

## This is DHARMa 0.4.7. For overview type '?DHARMa'. For recent changes, type news(package = 'DHARMa')
```

```
library(hms)
library(lubridate)
```

```
##
## Attaching package: 'lubridate'

## The following object is masked from 'package:hms':
##
##     hms

## The following objects are masked from 'package:base':
##
##     date, intersect, setdiff, union
```

```r
library(multcomp)
```

```
## Loading required package: mvtnorm

## Loading required package: survival

## Loading required package: TH.data

## Loading required package: MASS

##
## Attaching package: 'MASS'

## The following object is masked from 'package:dplyr':
##
##     select

##
## Attaching package: 'TH.data'

## The following object is masked from 'package:MASS':
##
##     geyser
```

```r
library(ggplot2)
```

```
## Warning: package 'ggplot2' was built under R version 4.3.3
```

```r
library(stringr)
library(gridExtra)
```

```
##
## Attaching package: 'gridExtra'

## The following object is masked from 'package:dplyr':
##
##     combine
```

```
library(viridis) #for letters on graph
```

```
## Loading required package: viridisLite
```

```
library(patchwork)
```

```
##
## Attaching package: 'patchwork'

## The following object is masked from 'package:MASS':
##
##     area
```

```
library(grid)
library(gridExtra)
```

Import data

```
#imports my data
Trap.im <- (read.csv("~/Desktop/trap.type.final/data/final.combined.bee.data.use.csv"))

#removed Missing year values column from data
Trap.im <- Trap.im %>% filter(!is.na(YEAR))

#changes all blanks to zeros
Trap.im [is.na(Trap.im)] <- 0

#sets variables to factor/date
Trap.im$YEAR <- as.factor(Trap.im$YEAR)
Trap.im$LOC <- as.factor(Trap.im$LOC)
Trap.im$Plot <- as.factor(Trap.im$Plot)
Trap.im$TRAP <- as.factor(Trap.im$TRAP)

#set time as time variable
Trap.im <- Trap.im %>%
  mutate(Time = as_hms(parse_date_time(Time, orders = "I:M p")))

#set date as date variable
Trap.im$DATE <- as.Date(Trap.im$DATE, format = "%m/%d/%y")

#hours as numeric
Trap.im$TRAP_HOURS <- as.numeric(Trap.im$TRAP_HOURS)

#Pivoting data for analysis
Trap <- Trap.im %>%   # Drop TIME column
  group_by(Plot, DATE, YEAR, TRAP, LOC) %>%
  summarise(
    TEMP = first(TEMP),
    TRAP_HOURS = sum(TRAP_HOURS, na.rm = TRUE),
    HB = sum(HB, na.rm = TRUE),
    BB = sum(BB, na.rm = TRUE),
```

```
    SQB = sum(SQB, na.rm = TRUE),
    SBB = sum(SBB, na.rm = TRUE),
    LBB = sum(LBB, na.rm = TRUE),
    SSB = sum(SSB, na.rm = TRUE),
    LSB = sum(LSB, na.rm = TRUE),
    GB = sum(GB, na.rm = TRUE),
    O = sum(O, na.rm = TRUE),
    .groups = "drop"
  )

#makes the Trap.novac data for morpohotaxa without vacuum
Trap.novac <- subset(Trap, TRAP != "VAC" )
Trap.novac <- subset(Trap, TRAP != "VAC " )

#pivots longer for violin graphing
TRAP.1 <-Trap %>% pivot_longer(cols=c('HB', 'BB', 'LBB', 'LSB', 'SBB', 'GB', 'O', 'SSB', 'SQB'),
                    names_to='MORPHO',
                    values_to='COUNT')

#sets data so visual observation is standard
Trap$TRAP <- factor(Trap$TRAP)
Trap$TRAP <- relevel(Trap$TRAP, ref = "VIS ")
Trap.novac$TRAP <- factor(Trap.novac$TRAP)
Trap.novac$TRAP <- relevel(Trap.novac$TRAP, ref = "VIS ")
```

Honey Bee

```
#checking for zero inflation in the data for honey bees using histogram and qq plots, shows data is ver
hist(Trap$HB)
```

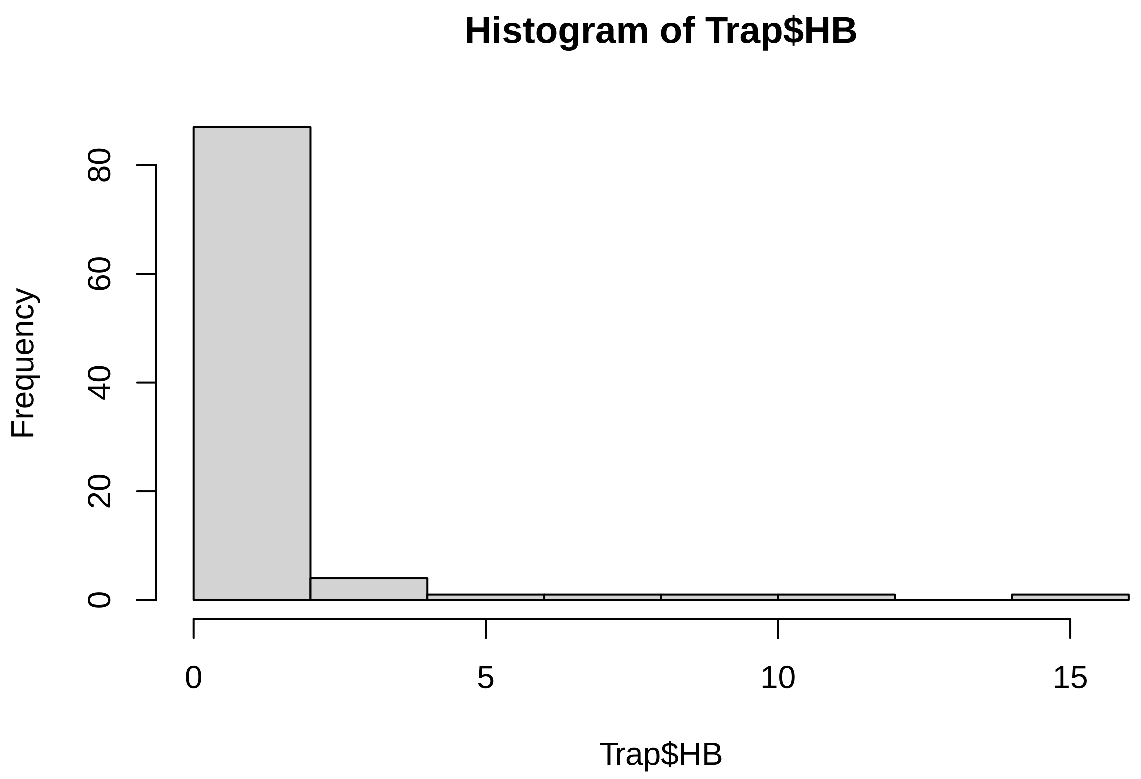


```r
qqnorm(Trap$HB)
```

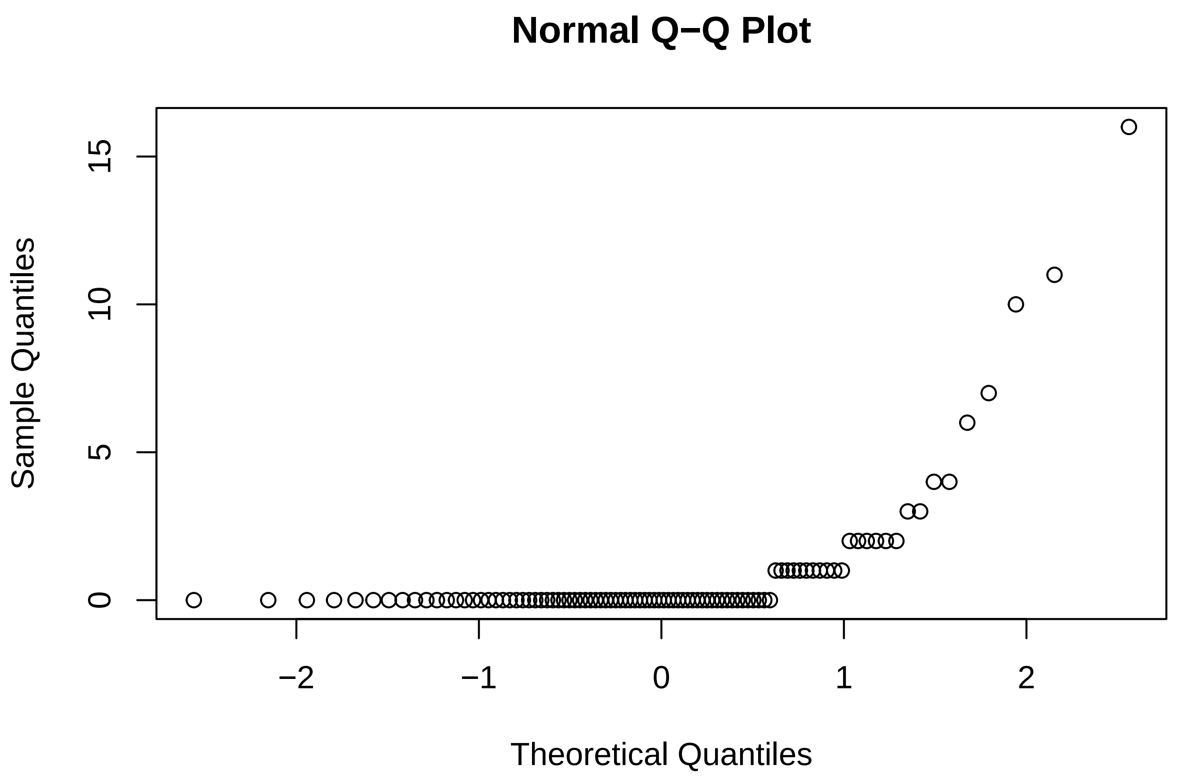


```r
#runs zero inflated glmm
model.HB <- glmmTMB(HB ~ TRAP  + (1 | DATE),
             ziformula = ~1,
             family = poisson(),
            data = Trap)
summary(model.HB)
```

```
##  Family: poisson  ( log )
## Formula:          HB ~ TRAP + (1 | DATE)
## Zero inflation:      ~1
## Data: Trap
##
##      AIC       BIC    logLik -2*log(L)  df.resid
##    205.1     217.9     -97.5     195.1        91
##
## Random effects:
##
## Conditional model:
##  Groups Name        Variance Std.Dev.
##  DATE   (Intercept) 4.447    2.109
## Number of obs: 96, groups:  DATE, 8
##
## Conditional model:
##              Estimate Std. Error z value Pr(>|z|)
## (Intercept)  -0.8991     1.1036  -0.815 0.415225
## TRAPBowl     -2.0877     0.5412  -3.858 0.000115 ***
```

```
## TRAPVAC      -0.8339     0.2900  -2.876 0.004031 **
## ---
## Signif. codes:  0 '***' 0.001 '**' 0.01 '*' 0.05 '.' 0.1 ' ' 1
##
## Zero-inflation model:
##             Estimate Std. Error z value Pr(>|z|)
## (Intercept)  -0.5820     0.4641  -1.254     0.21
```

```r
#Runs the DHARMa residuals to test distrbutions and model fits
sim_res.HB <- simulateResiduals(fittedModel = model.HB, plot = TRUE)
```

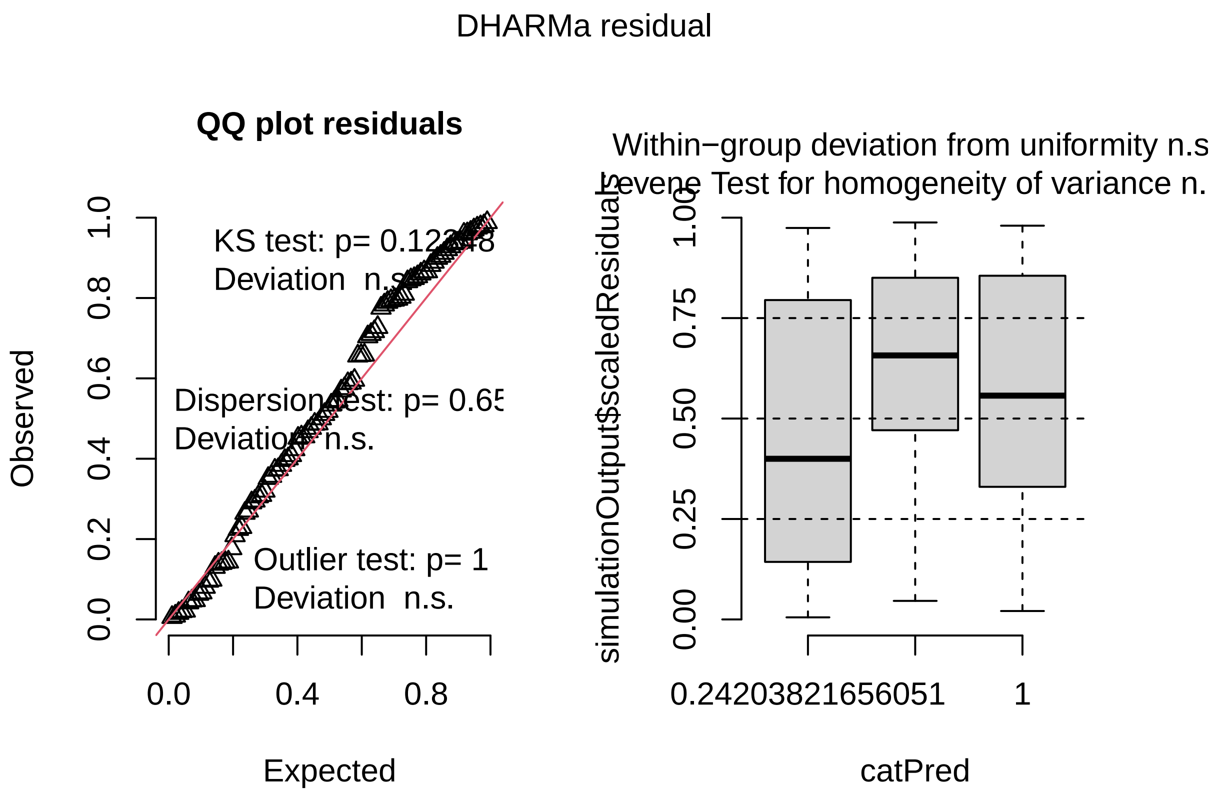


```r
testResiduals(sim_res.HB)
```

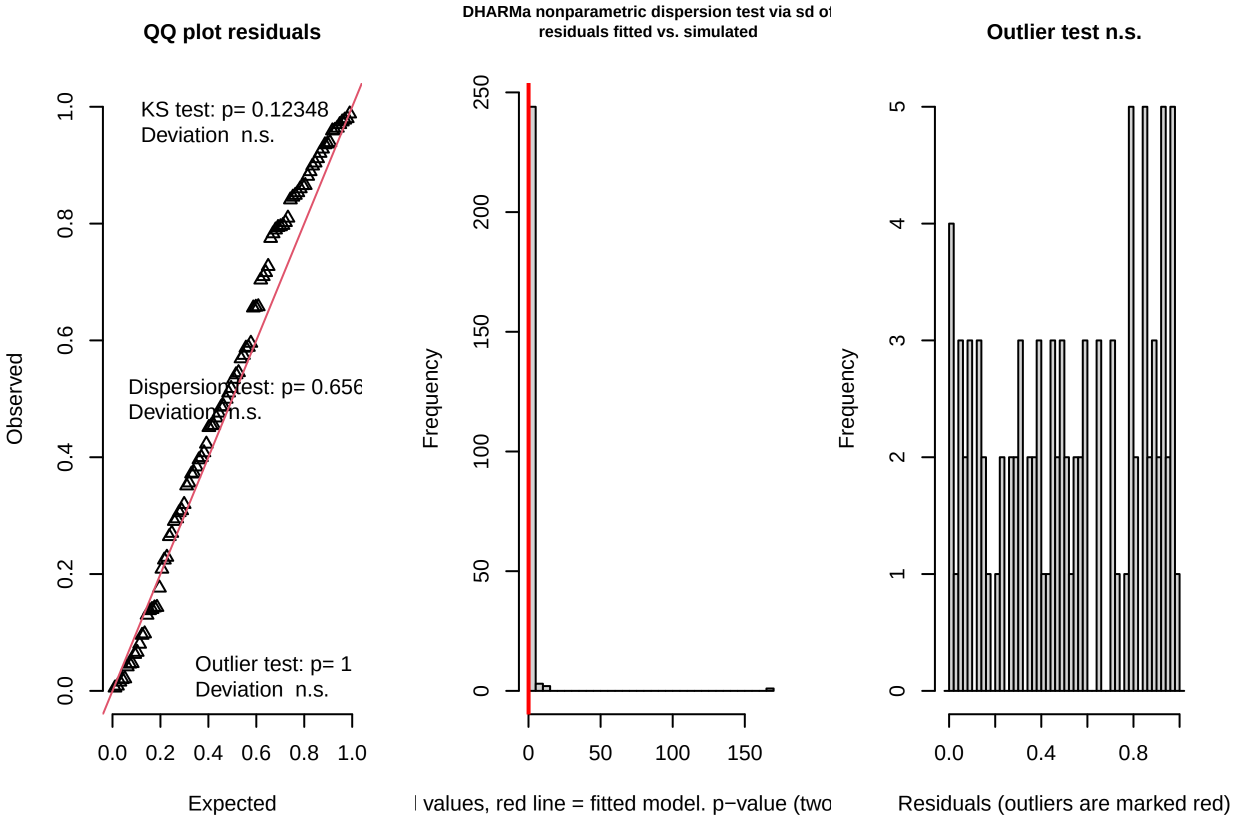


```
## $uniformity
##
##  Exact one-sample Kolmogorov-Smirnov test
##
## data:  simulationOutput$scaledResiduals
## D = 0.11865, p-value = 0.1235
## alternative hypothesis: two-sided
##
##
## $dispersion
##
##  DHARMa nonparametric dispersion test via sd of residuals fitted vs.
##  simulated
##
## data:  simulationOutput
## dispersion = 0.049634, p-value = 0.656
## alternative hypothesis: two.sided
##
##
## $outliers
##
##  DHARMa bootstrapped outlier test
##
## data:  simulationOutput
## outliers at both margin(s) = 0, observations = 96, p-value = 1
## alternative hypothesis: two.sided
```

```
##  percent confidence interval:
##  0.00000000 0.06796875
## sample estimates:
## outlier frequency (expected: 0.00708333333333333 )
##                                                 0
```

```r
testDispersion(sim_res.HB)
```

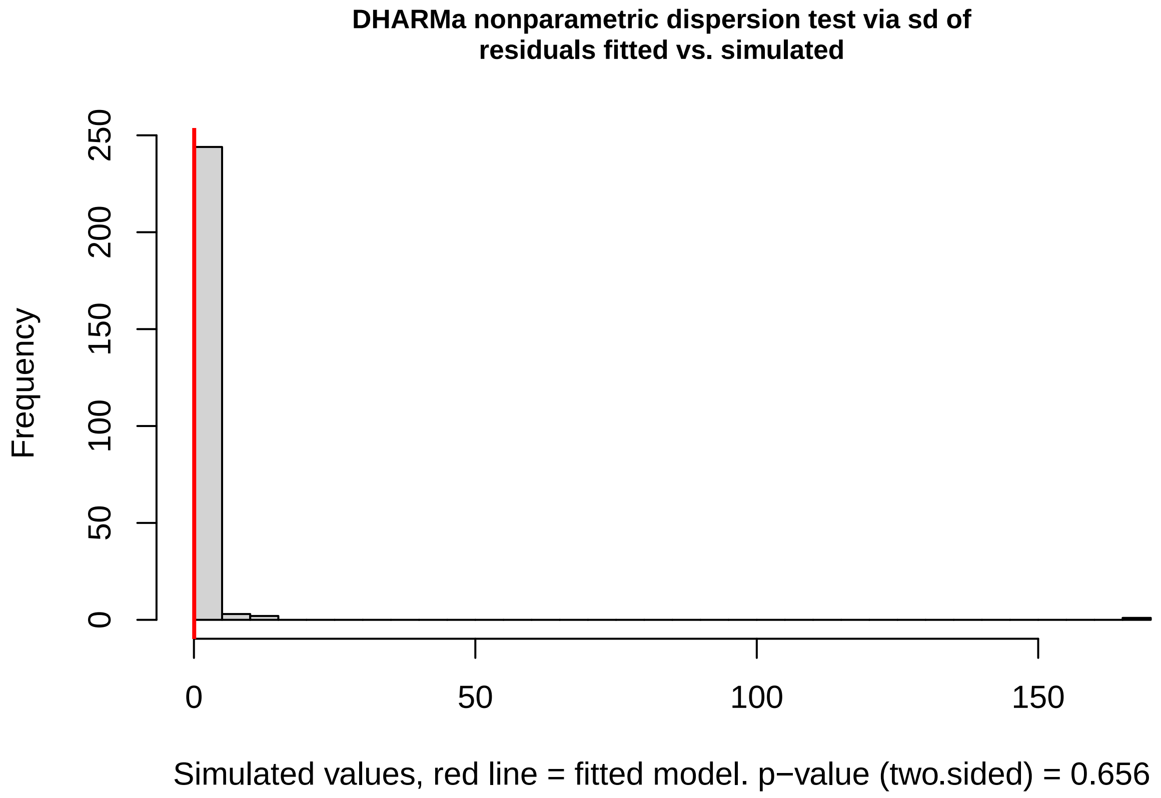


```
##
##  DHARMa nonparametric dispersion test via sd of residuals fitted vs.
##  simulated
##
## data:  simulationOutput
## dispersion = 0.049634, p-value = 0.656
## alternative hypothesis: two.sided
```

```r
testZeroInflation(sim_res.HB)
```

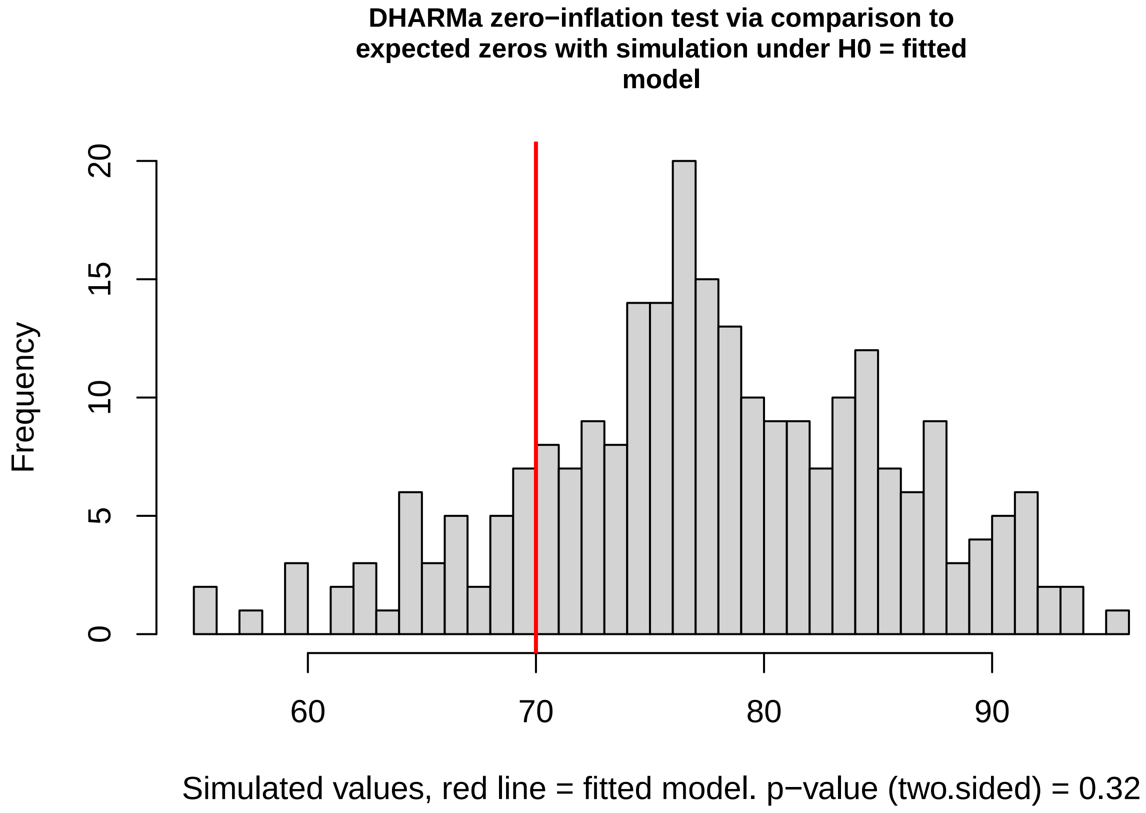


```
##
##  DHARMa zero-inflation test via comparison to expected zeros with
##  simulation under H0 = fitted model
##
## data:  simulationOutput
## ratioObsSim = 0.89656, p-value = 0.32
## alternative hypothesis: two.sided
```

```
# Reduced model for LRT
hb_reduced <- glmmTMB(HB ~ 1  + (1 | DATE),
             ziformula = ~1,
             family = poisson(),
            data = Trap)

# Likelihood ratio test
anova(model.HB, hb_reduced, test = "Chisq")
```

```
## Data: Trap
## Models:
## hb_reduced: HB ~ 1 + (1 | DATE), zi=~1, disp=~1
## model.HB: HB ~ TRAP + (1 | DATE), zi=~1, disp=~1
##            Df    AIC    BIC   logLik deviance  Chisq Chi Df Pr(>Chisq)
## hb_reduced  3 223.83 231.52 -108.914   217.83
## model.HB    5 205.09 217.91  -97.545   195.09 22.739      2  1.154e-05 ***
## ---
## Signif. codes:  0 '***' 0.001 '**' 0.01 '*' 0.05 '.' 0.1 ' ' 1
```

```r
#running emmeans with sidak
marginal.hb = emmeans(model.HB, ~ TRAP, type = "response")

hb.cld <- cld(marginal.hb,
    alpha=0.05,
    Letters=letters,  ### Use lower-case letters for .group
    adjust="sidak")

# Clean up for plotting to add letters to plot
hb.cld_result <- as.data.frame(hb.cld)
hb.cld_result$group <- as.character(hb.cld_result$.group)

#PULL ONLY HB for graphing
HB.PLOT <- TRAP.1 %>% filter(MORPHO == 'HB')
Count.HB <- HB.PLOT %>% select_("TRAP", "MORPHO", "COUNT")
```

```
## Warning: `select_()` was deprecated in dplyr 0.7.0.
## i Please use `select()` instead.
## Call `lifecycle::last_lifecycle_warnings()` to see where this warning was
## generated.
```

```r
# HB violin plot
p.hb <-ggplot(HB.PLOT, aes(x=TRAP, y=COUNT, fill=TRAP)) +
  geom_violin() + scale_x_discrete(labels = c("Bowl" = "Bowl",
                              "VAC " = "Vacuum",
                              "VIS " = "Visual")) + labs(y = "Counts per 15 m")  #renames variables

p.hb.1 <- p.hb + theme(axis.title.x=element_blank(),axis.title.y = element_text(size = 10))  # removes 

HB.final.plot <- p.hb.1 + theme(legend.position = "none", plot.title = element_text(size = 10)) + ggtit

HB.final.plot
```

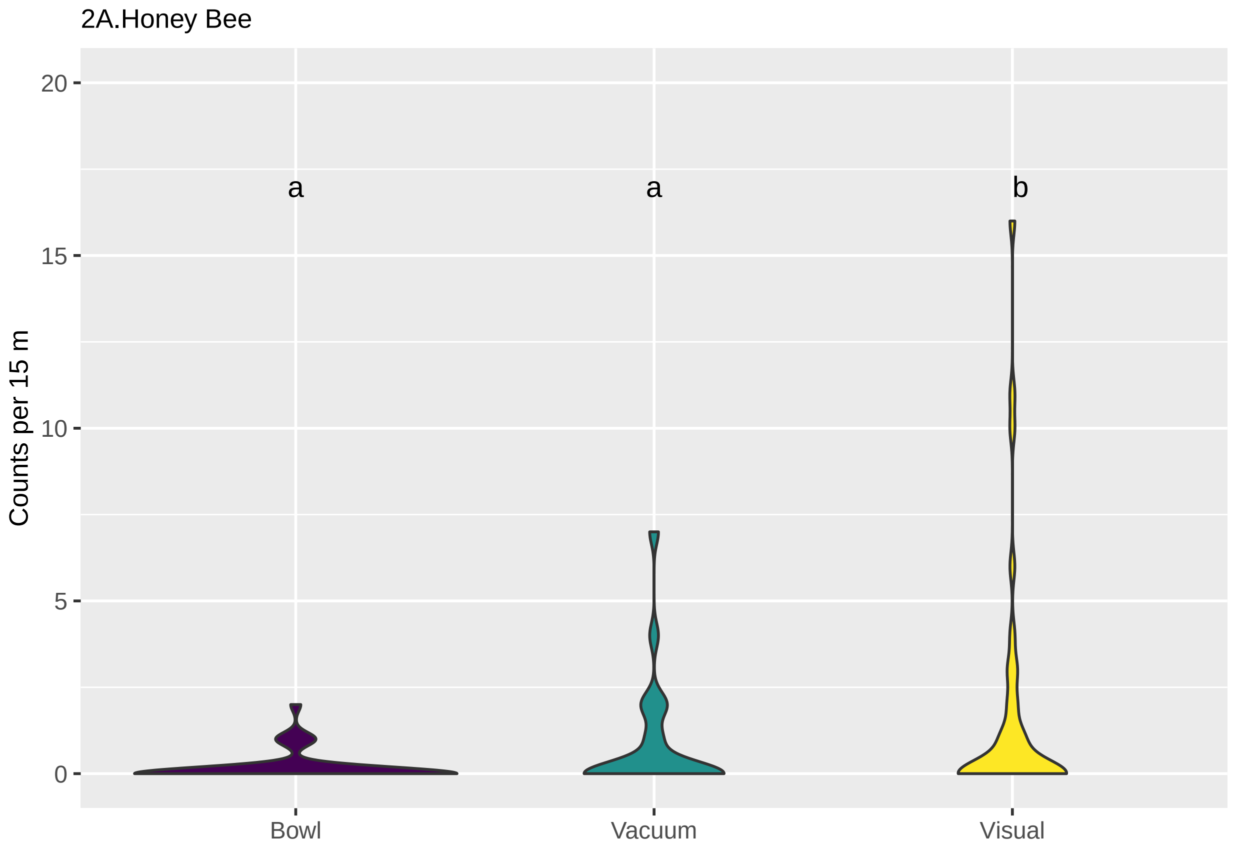

Bumble Bee

```
# Checking data for zeroinflation
hist(Trap$BB)
```

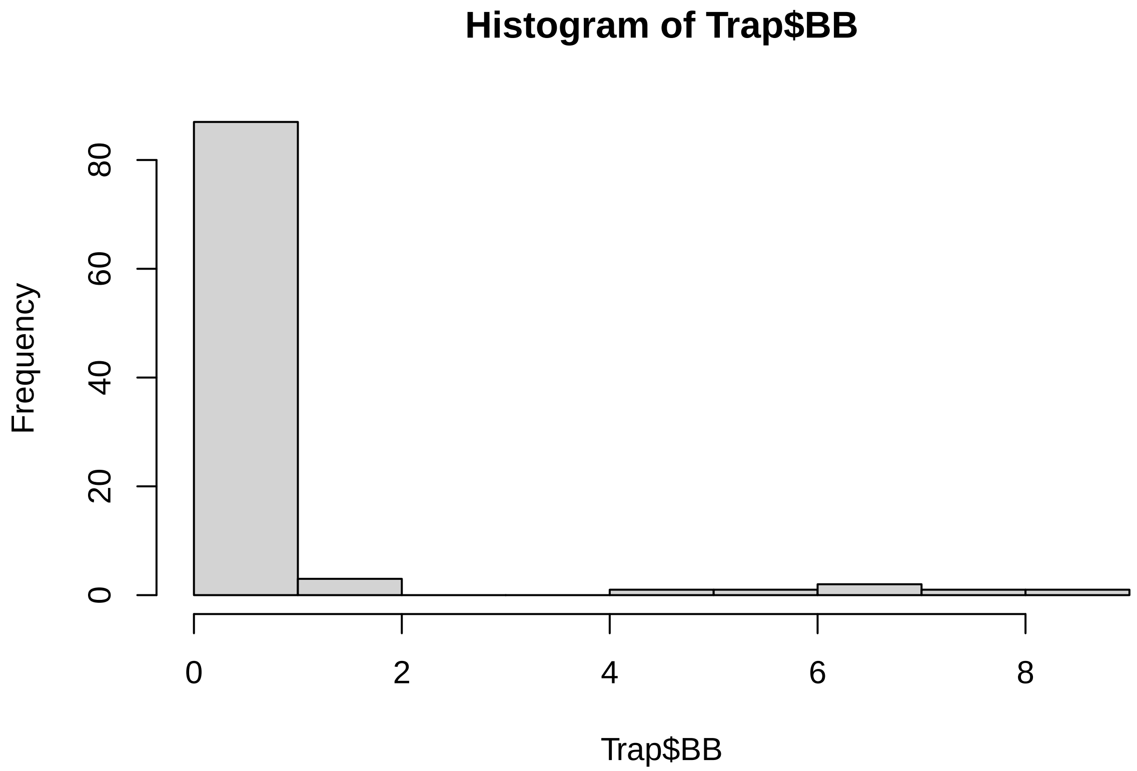


```
qqnorm(Trap$BB)
```

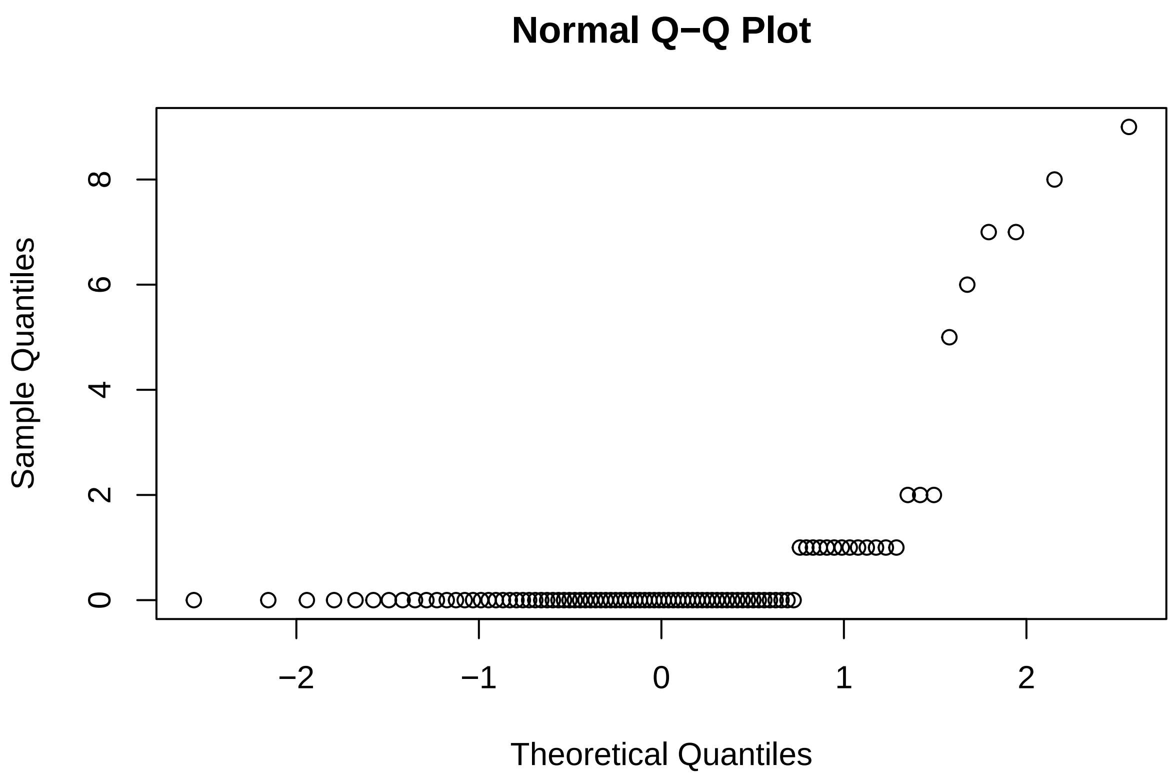


```r
#glmm zero inflated
model.BB <- glmmTMB(BB ~ TRAP + (1 | DATE),
                    ziformula = ~1,
                    family = poisson(),
                    data = Trap.novac)
summary(model.BB)
```

```
##  Family: poisson  ( log )
## Formula:          BB ~ TRAP + (1 | DATE)
## Zero inflation:      ~1
## Data: Trap.novac
##
##      AIC      BIC   logLik -2*log(L)  df.resid
##    112.8    122.0    -52.4     104.8        69
##
## Random effects:
##
## Conditional model:
##  Groups Name        Variance Std.Dev.
##  DATE   (Intercept) 2.213    1.488
## Number of obs: 73, groups:  DATE, 8
##
## Conditional model:
##             Estimate Std. Error z value Pr(>|z|)
## (Intercept)  -0.8239     0.6514  -1.265    0.206
## TRAPBowl     -2.1878     0.4344  -5.037 4.74e-07 ***
```

```
## ---
## Signif. codes:  0 '***' 0.001 '**' 0.01 '*' 0.05 '.' 0.1 ' ' 1
##
## Zero-inflation model:
##             Estimate Std. Error z value Pr(>|z|)
## (Intercept)    -21.4    14640.3  -0.001    0.999
```

```
#Runs the DHARMa residuals to test distrbutions and model fits
sim_res.BB <- simulateResiduals(fittedModel = model.BB, plot = TRUE)
```

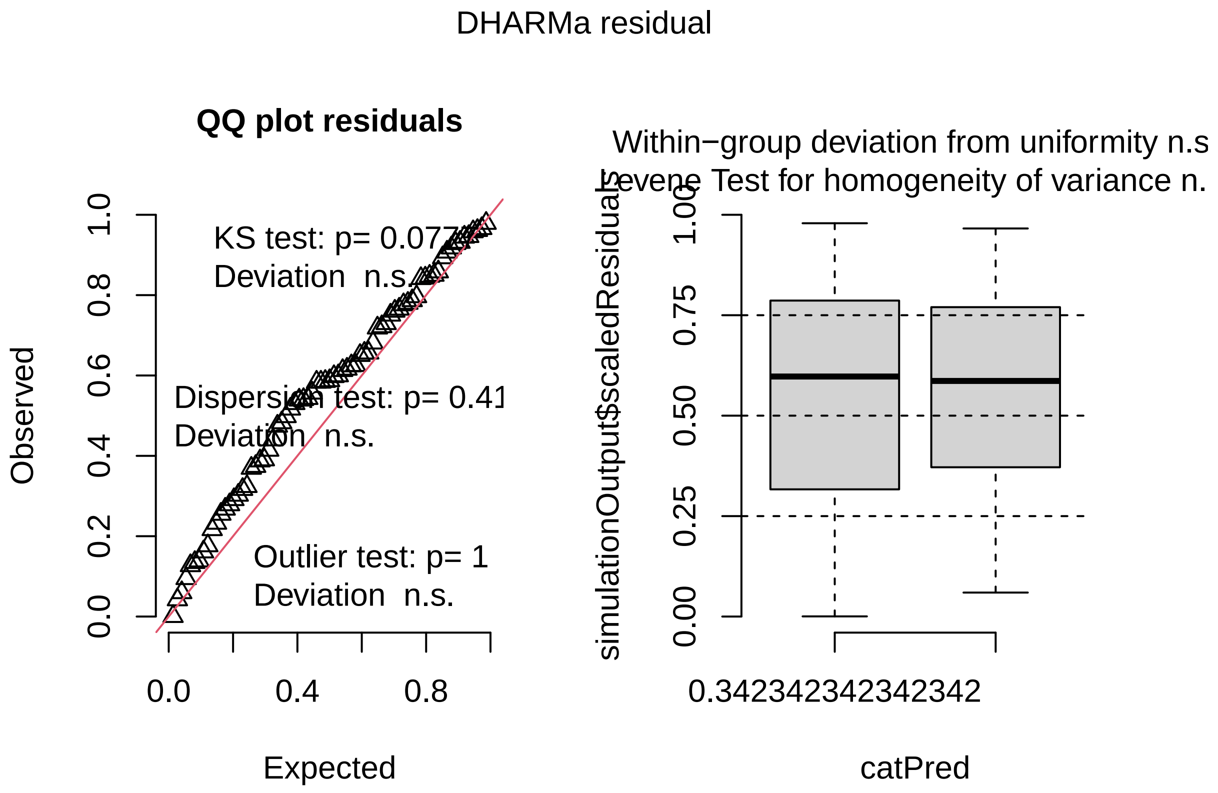


```
testResiduals(sim_res.BB)
```

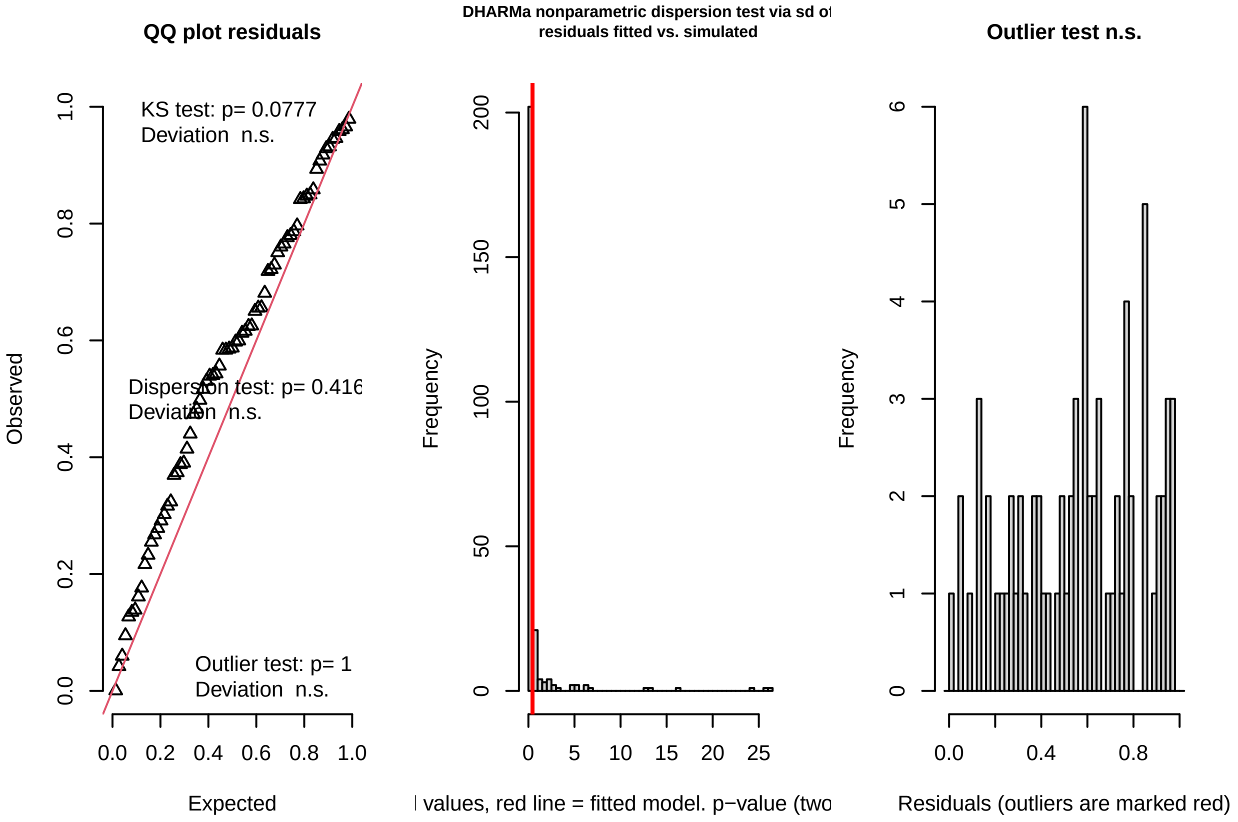


```
## $uniformity
##
##  Exact one-sample Kolmogorov-Smirnov test
##
## data:  simulationOutput$scaledResiduals
## D = 0.14675, p-value = 0.0777
## alternative hypothesis: two-sided
##
##
## $dispersion
##
##  DHARMa nonparametric dispersion test via sd of residuals fitted vs.
##  simulated
##
## data:  simulationOutput
## dispersion = 0.49117, p-value = 0.416
## alternative hypothesis: two.sided
##
##
## $outliers
##
##  DHARMa bootstrapped outlier test
##
## data:  simulationOutput
## outliers at both margin(s) = 0, observations = 73, p-value = 1
## alternative hypothesis: two.sided
```

```
##  percent confidence interval:
##  0.00000000 0.06986301
## sample estimates:
## outlier frequency (expected: 0.00575342465753425 )
##                                                    0
```

```r
testDispersion(sim_res.BB)
```

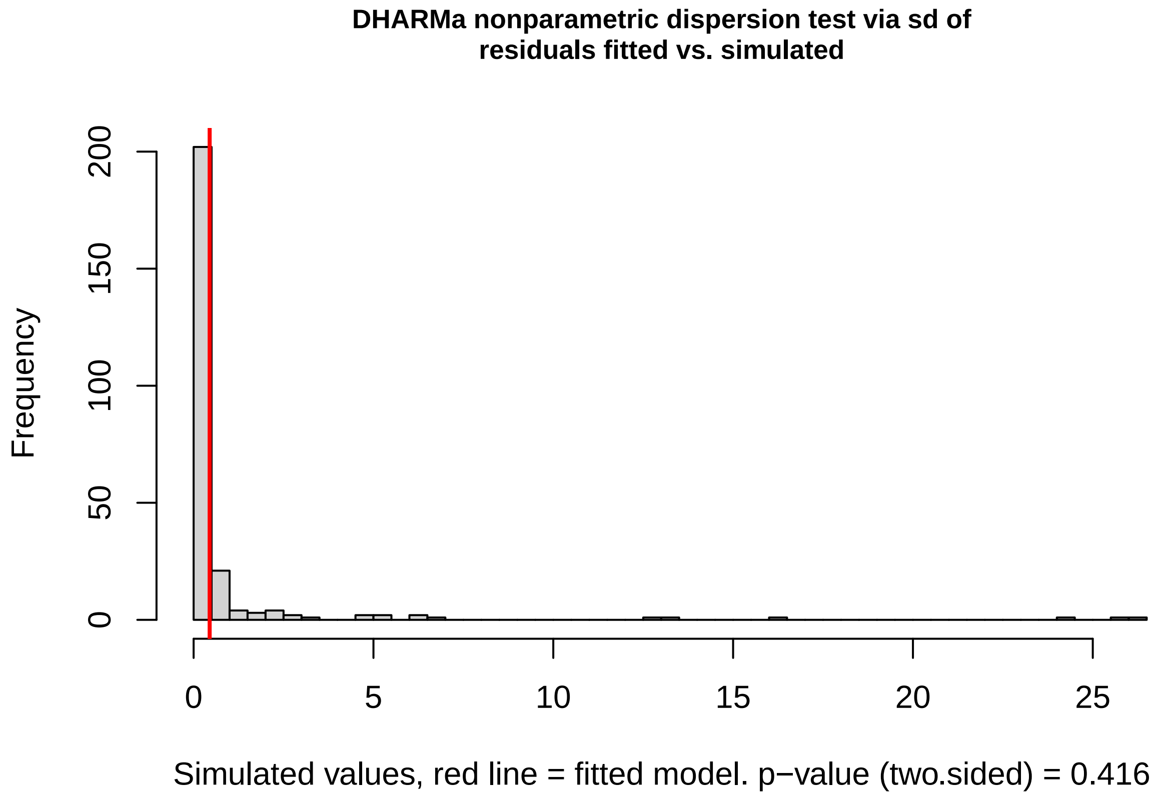


```
##
##  DHARMa nonparametric dispersion test via sd of residuals fitted vs.
##  simulated
##
## data:  simulationOutput
## dispersion = 0.49117, p-value = 0.416
## alternative hypothesis: two.sided
```

```r
testZeroInflation(sim_res.BB)
```

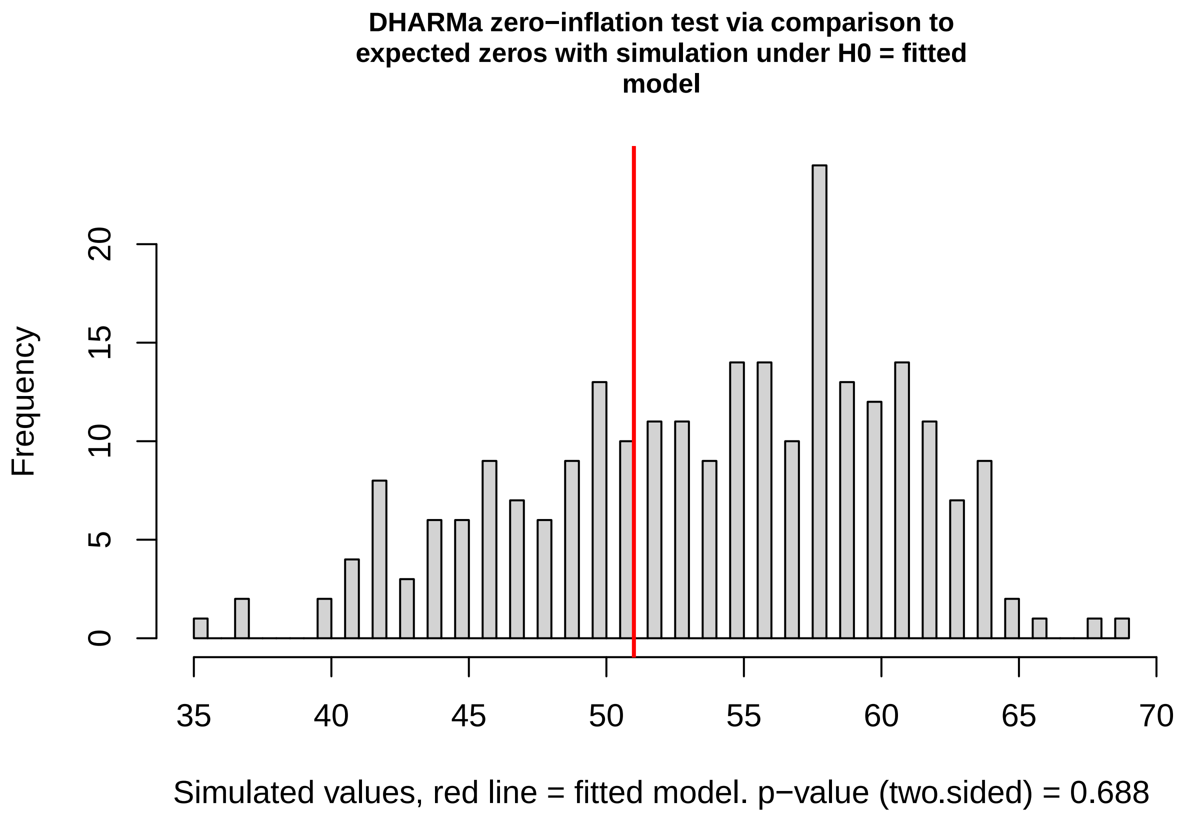


```
##
##  DHARMa zero-inflation test via comparison to expected zeros with
##  simulation under H0 = fitted model
##
## data:  simulationOutput
## ratioObsSim = 0.94347, p-value = 0.688
## alternative hypothesis: two.sided
```

```r
# Reduced model for LRT
bb_reduced <- glmmTMB(BB ~ 1 + (1 | DATE),
             ziformula = ~1,
             family = poisson(),
            data = Trap.novac)

# Likelihood ratio test
anova(model.BB, bb_reduced, test = "Chisq")
```

```
## Data: Trap.novac
## Models:
## bb_reduced: BB ~ 1 + (1 | DATE), zi=~1, disp=~1
## model.BB: BB ~ TRAP + (1 | DATE), zi=~1, disp=~1
##            Df    AIC    BIC  logLik deviance Chisq Chi Df Pr(>Chisq)
## bb_reduced  3 147.09 153.96 -70.547   141.09
## model.BB    4 112.83 122.00 -52.417   104.83 36.26      1  1.727e-09 ***
## ---
## Signif. codes:  0 '***' 0.001 '**' 0.01 '*' 0.05 '.' 0.1 ' ' 1
```

```r
#running emmeans with sidak
em.BB <- emmeans(model.BB, "TRAP", type = "response" )
bb.cld <- cld(em.BB,
    alpha=0.05,
    Letters=letters,  ### Use lower-case letters for .group
    adjust="sidak")

# Clean up for plotting to add letters to plot
bb.cld_result <- as.data.frame(bb.cld)
bb.cld_result$group <- as.character(bb.cld_result$.group)

#PULL ONLY BB, for graphing purposes
BB.PLOT <- TRAP.1 %>% filter(MORPHO == 'BB')
Count.BB <- BB.PLOT %>% select_("TRAP", "MORPHO", "COUNT")
```

```
## Warning: `select_()` was deprecated in dplyr 0.7.0.
## i Please use `select()` instead.
## Call `lifecycle::last_lifecycle_warnings()` to see where this warning was
## generated.
```

```r
# BB violin plot
p.bb <-ggplot(BB.PLOT, aes(x=TRAP, y=COUNT, fill=TRAP)) +
  geom_violin() + scale_x_discrete(labels = c("Bowl" = "Bowl",
                              "VAC " = "Vacuum",
                              "VIS " = "Visual"))  + labs(y = "Counts per 15 m")
p.bb.1 <- p.bb + theme(axis.title.x=element_blank(),axis.title.y = element_text(size = 10))

# adds title and axis settings
BB.final.plot <- p.bb.1 +
  scale_fill_viridis_d() + ggtitle("2D.Bumble Bee") + coord_cartesian(ylim = c(0, 20)) + theme(legend.p

BB.final.plot
```

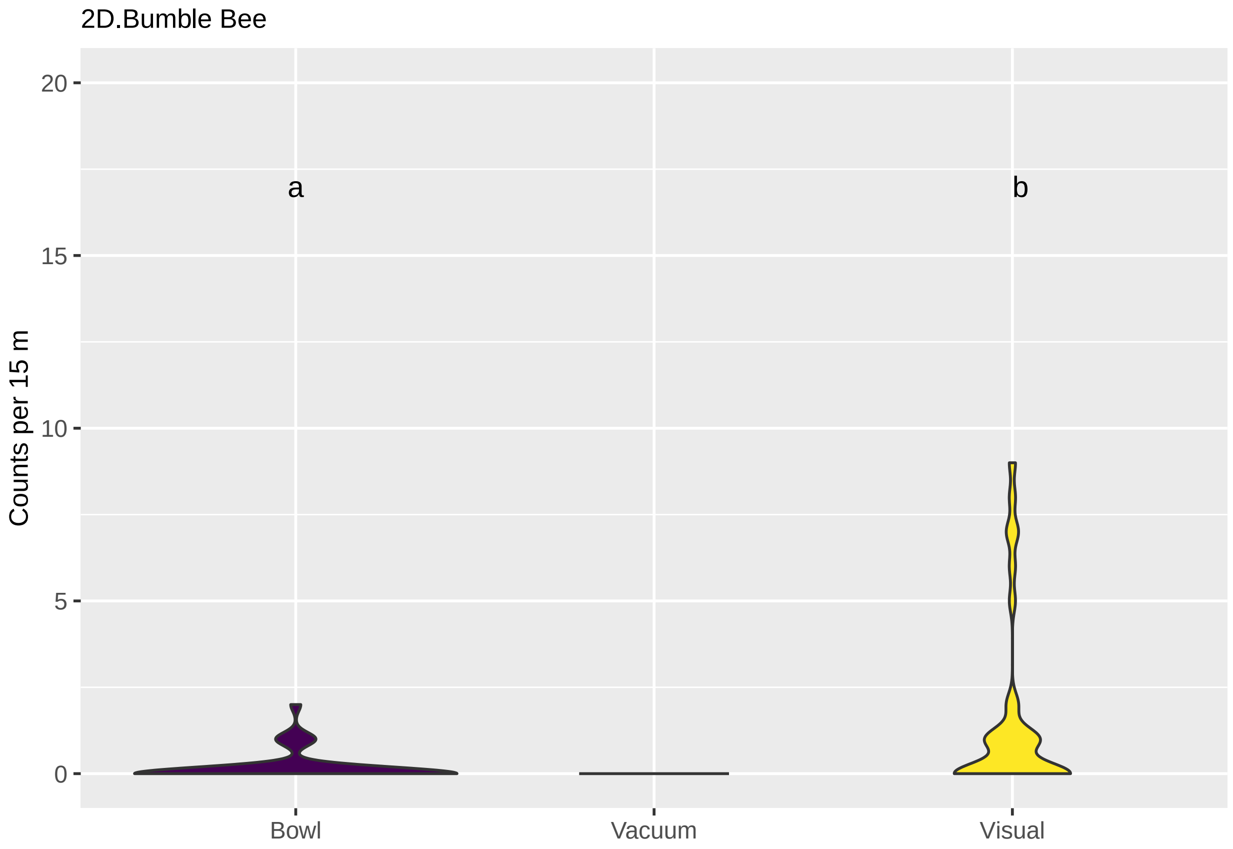

Squash Bee

```
#checking for zero inflation
hist(Trap$SQB)
```

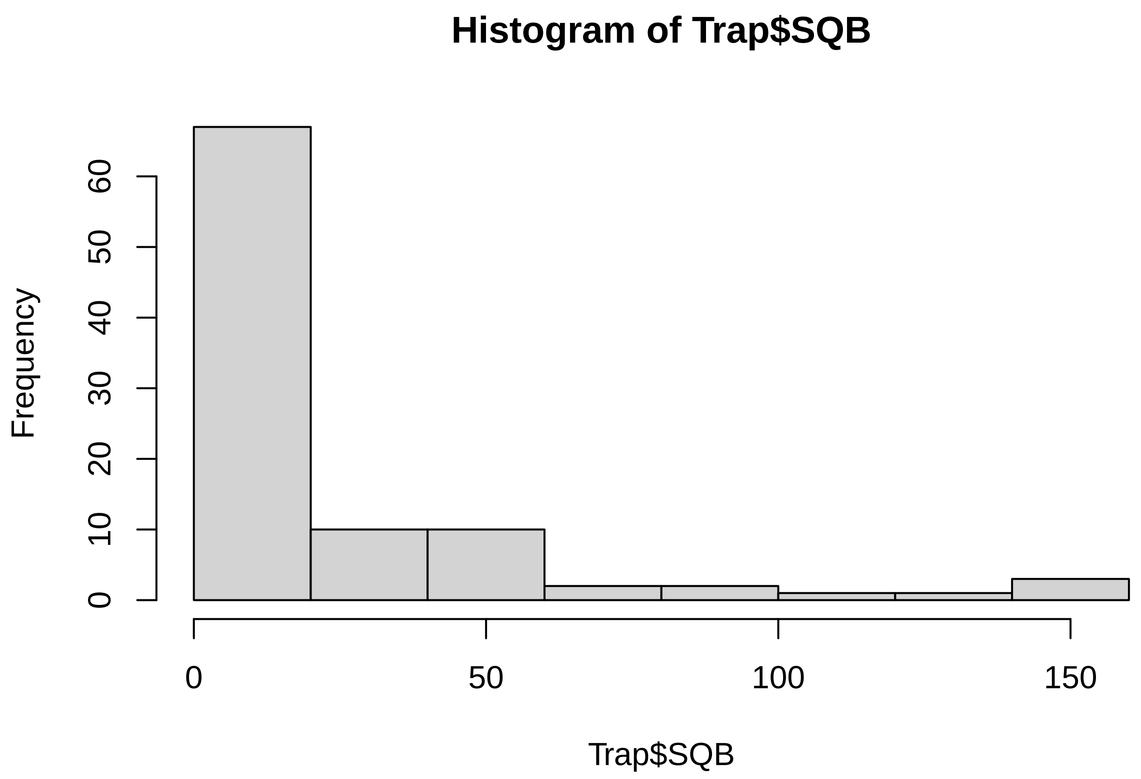


```
qqnorm(Trap$SQB)
```

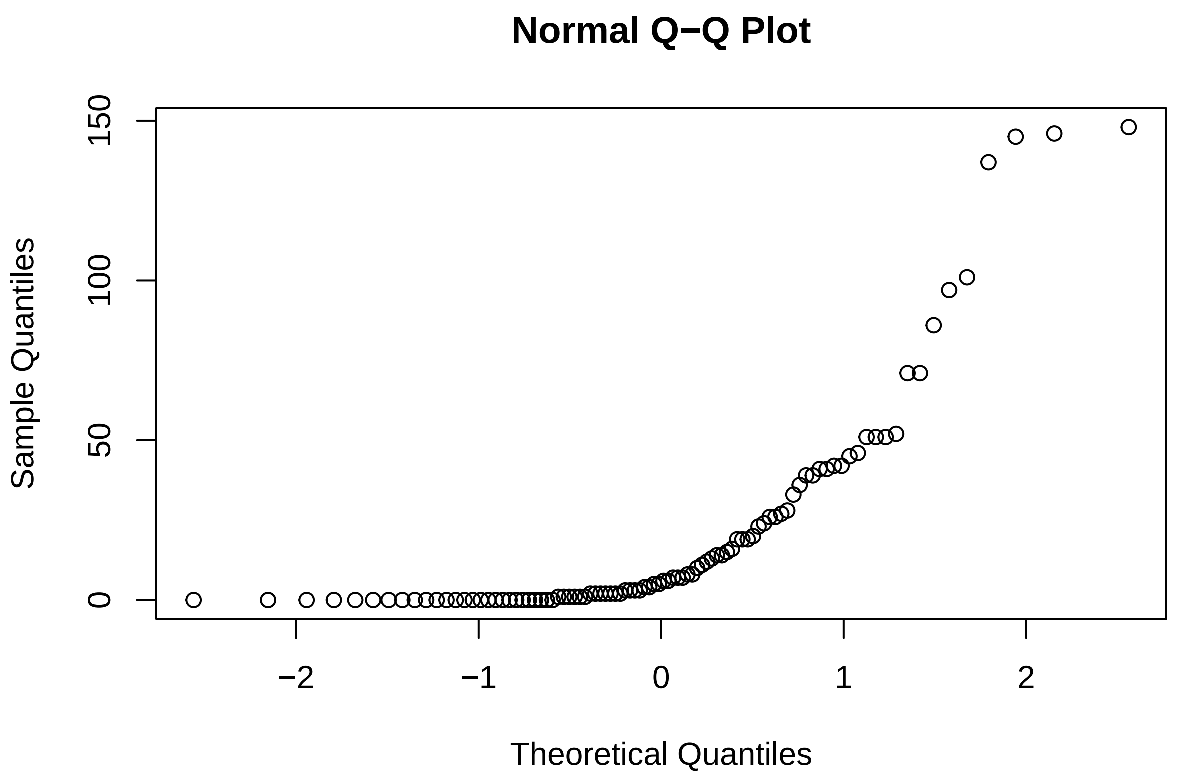


```r
#glmm zero inflated, with HB count as response and Trap as fixed variable with Location and year as ran
model.SQB <- glmmTMB(SQB ~ TRAP + (1 | LOC) + (1 | DATE),
                  ziformula = ~1,
                  family = nbinom12(),
                  data = Trap)

summary(model.SQB)
```

```
##  Family: nbinom12  ( log )
## Formula:          SQB ~ TRAP + (1 | LOC) + (1 | DATE)
## Zero inflation:       ~1
## Data: Trap
##
##      AIC       BIC    logLik -2*log(L)  df.resid
##    590.1     610.6    -287.0     574.1        88
##
## Random effects:
##
## Conditional model:
##  Groups Name        Variance Std.Dev.
##  LOC    (Intercept) 0.8057   0.8976
##  DATE   (Intercept) 3.1179   1.7658
## Number of obs: 96, groups:  LOC, 3; DATE, 8
##
## Dispersion parameter for nbinom12 family (): 5.81
##
```

```
## Conditional model:
##              Estimate Std. Error z value Pr(>|z|)
## (Intercept)    2.3775     0.8961   2.653  0.00797 **
## TRAPBowl      -1.1991     0.1626  -7.375 1.64e-13 ***
## TRAPVAC       -0.5081     0.1927  -2.636  0.00838 **
## ---
## Signif. codes:  0 '***' 0.001 '**' 0.01 '*' 0.05 '.' 0.1 ' ' 1
##
## Zero-inflation model:
##              Estimate Std. Error z value Pr(>|z|)
## (Intercept)    -4.294      1.430  -3.004  0.00267 **
## ---
## Signif. codes:  0 '***' 0.001 '**' 0.01 '*' 0.05 '.' 0.1 ' ' 1
```

```r
#Runs the DHARMa residuals to test distrbutions and model fits
sim_res.SQB <- simulateResiduals(fittedModel = model.SQB, plot = TRUE)
```

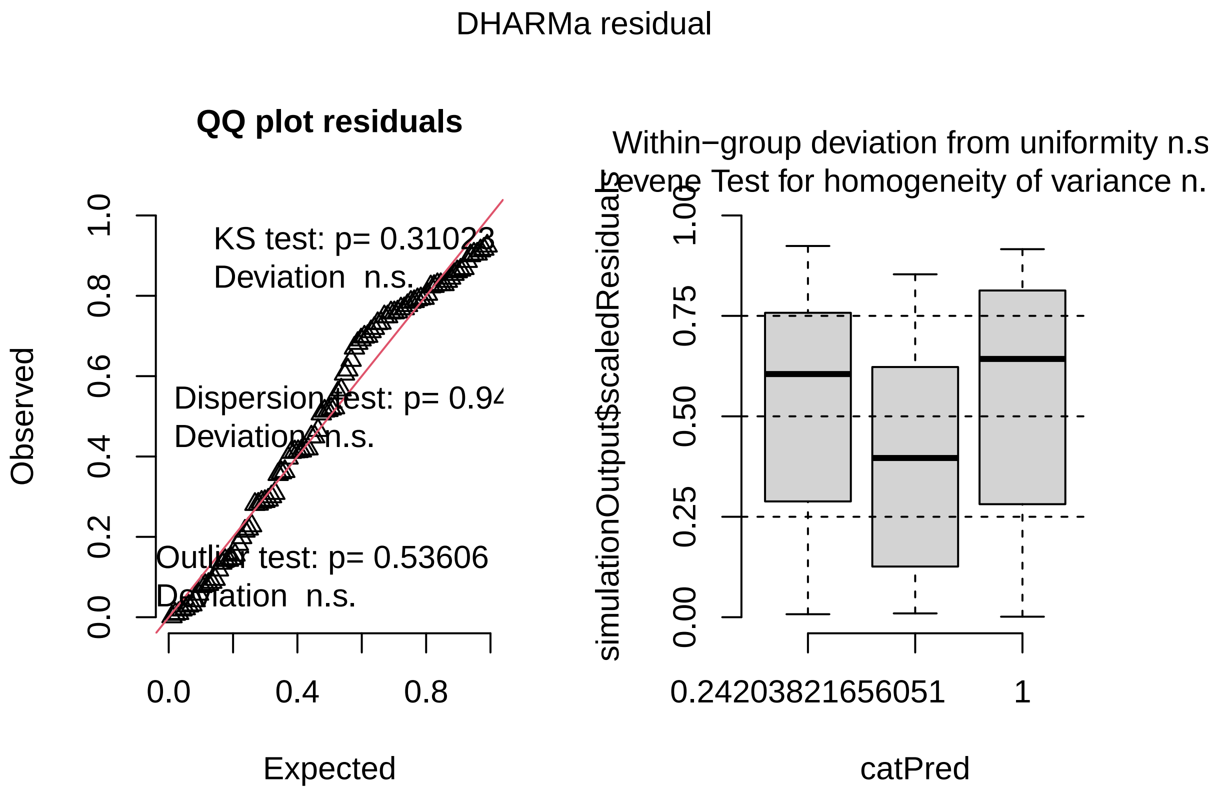


```r
testResiduals(sim_res.SQB)
```

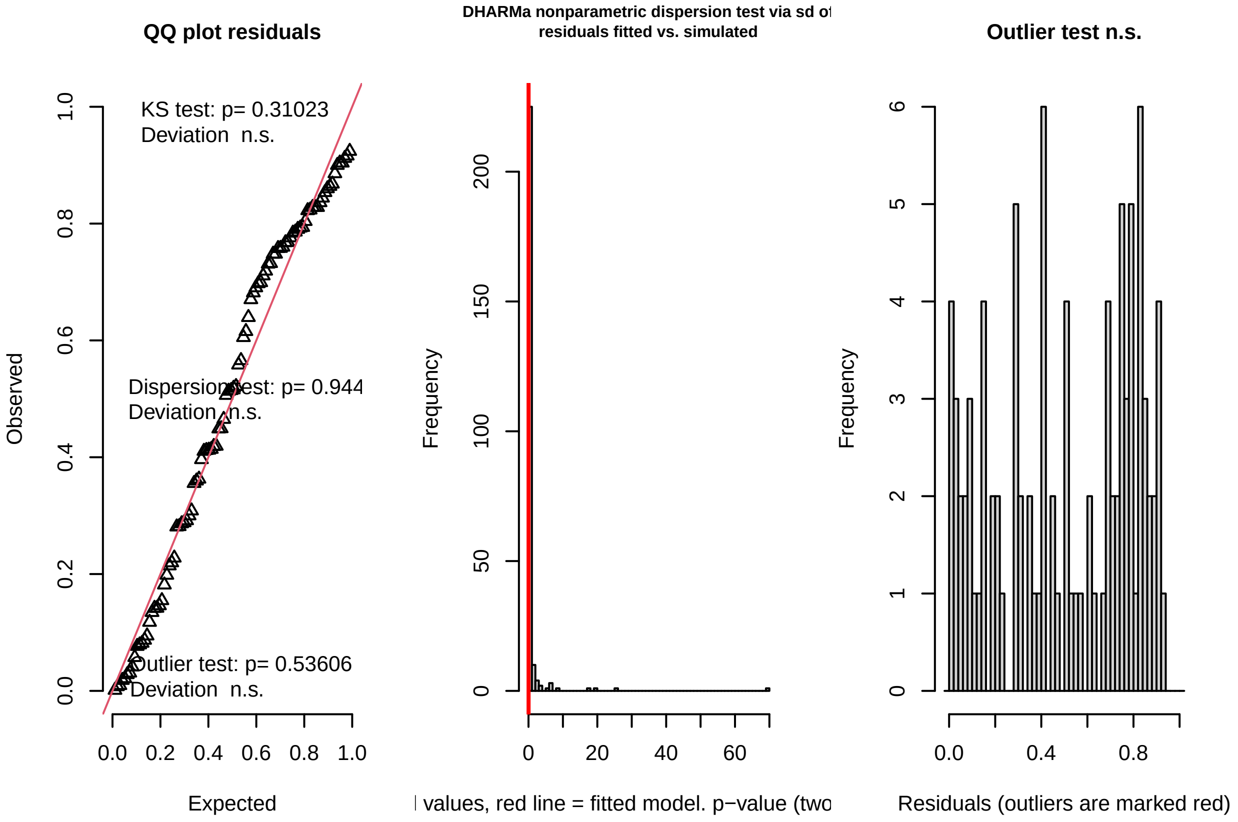


```
## $uniformity
##
##  Asymptotic one-sample Kolmogorov-Smirnov test
##
## data:  simulationOutput$scaledResiduals
## D = 0.098422, p-value = 0.3102
## alternative hypothesis: two-sided
##
##
## $dispersion
##
##  DHARMa nonparametric dispersion test via sd of residuals fitted vs.
##  simulated
##
## data:  simulationOutput
## dispersion = 0.026951, p-value = 0.944
## alternative hypothesis: two.sided
##
##
## $outliers
##
##  DHARMa outlier test based on exact binomial test with approximate
##  expectations
##
## data:  simulationOutput
## outliers at both margin(s) = 1, observations = 96, p-value = 0.5361
```

```
## alternative hypothesis: true probability of success is not equal to 0.007968127
## 95 percent confidence interval:
##  0.0002636924 0.0566747070
## sample estimates:
## frequency of outliers (expected: 0.00796812749003984 )
##                                              0.01041667
```

```r
testDispersion(sim_res.SQB)
```

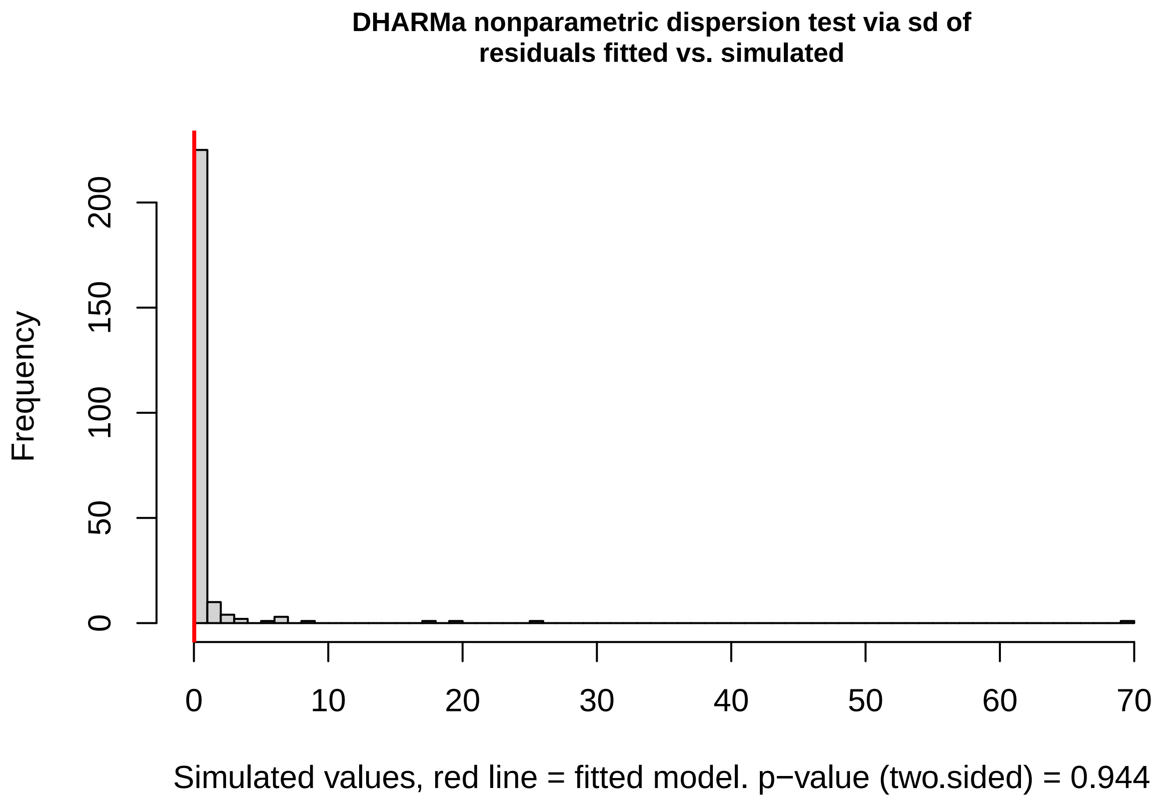


```
##
##  DHARMa nonparametric dispersion test via sd of residuals fitted vs.
##  simulated
##
## data:  simulationOutput
## dispersion = 0.026951, p-value = 0.944
## alternative hypothesis: two.sided
```

```r
testZeroInflation(sim_res.SQB)
```

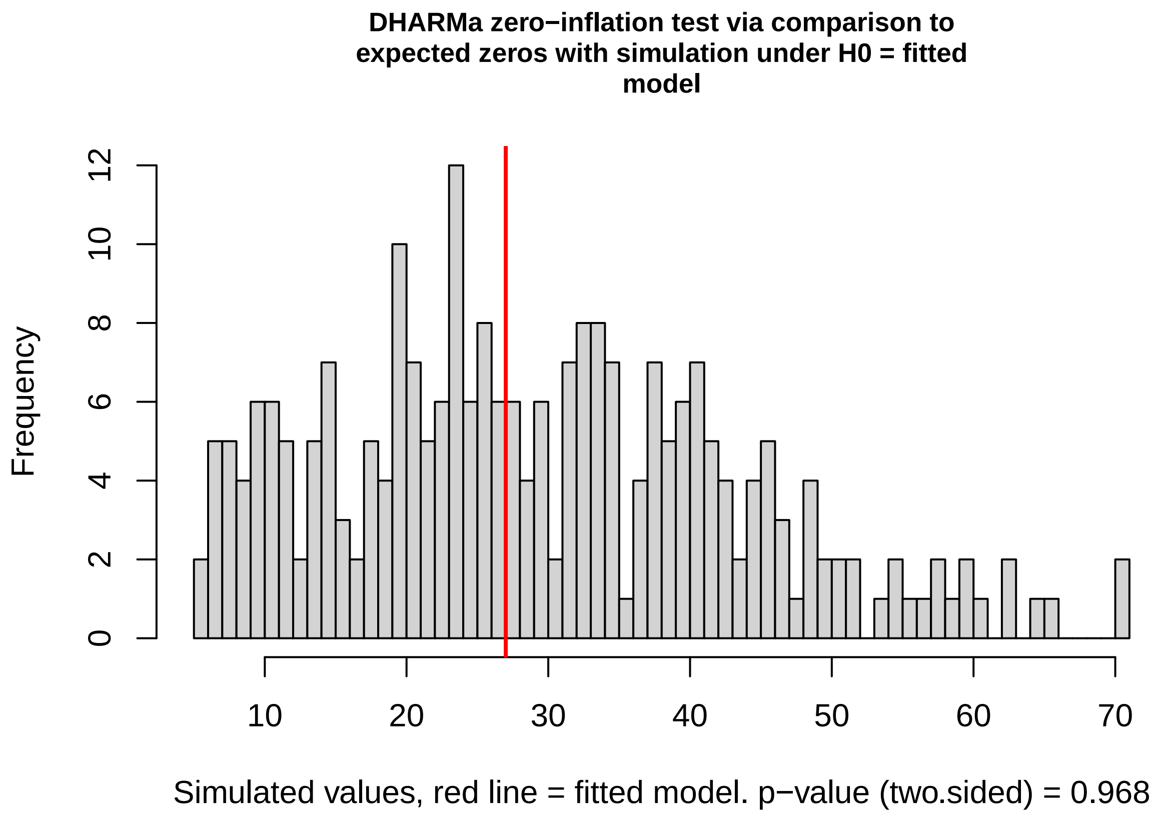


```
##
##  DHARMa zero-inflation test via comparison to expected zeros with
##  simulation under H0 = fitted model
##
## data:  simulationOutput
## ratioObsSim = 0.9041, p-value = 0.968
## alternative hypothesis: two.sided
```

```r
# Reduced model for LRT
sqb_reduced <- glmmTMB(SQB ~ 1 + (1 | LOC) + (1 | DATE),
                  ziformula = ~1,
                  family = nbinom12(),
                  data = Trap)

# Likelihood ratio test
anova(model.SQB, sqb_reduced, test = "Chisq")
```

```
## Data: Trap
## Models:
## sqb_reduced: SQB ~ 1 + (1 | LOC) + (1 | DATE), zi=~1, disp=~1
## model.SQB: SQB ~ TRAP + (1 | LOC) + (1 | DATE), zi=~1, disp=~1
##             Df    AIC    BIC  logLik deviance  Chisq Chi Df Pr(>Chisq)
## sqb_reduced  6 610.36 625.75 -299.18   598.36
## model.SQB    8 590.09 610.60 -287.04   574.09 24.274      2  5.357e-06 ***
## ---
## Signif. codes:  0 '***' 0.001 '**' 0.01 '*' 0.05 '.' 0.1 ' ' 1
```

```
#running emmeans with sidak
marginal.SQB = emmeans(model.SQB,
                   ~ TRAP, type = "response")
sqb.cld <- cld(marginal.SQB,
    alpha=0.05,
    Letters=letters,  ### Use lower-case letters for .group
    adjust="sidak")

# Clean up for plotting to add letters to plot
sqb.cld_result <- as.data.frame(sqb.cld)
sqb.cld_result$group <- as.character(sqb.cld_result$.group)


#PULL ONLY SQB for graphing
SQB.PLOT <- TRAP.1 %>% filter(MORPHO == 'SQB')
Count.SQB <- SQB.PLOT %>% select_("TRAP", "MORPHO", "COUNT")
```

```
## Warning: `select_()` was deprecated in dplyr 0.7.0.
## i Please use `select()` instead.
## Call `lifecycle::last_lifecycle_warnings()` to see where this warning was
## generated.
```

```
#plotting the SQB Viloin Graph
p.sqb <-ggplot(Count.SQB, aes(x=TRAP, y=COUNT, fill=TRAP)) +
  geom_violin() + scale_x_discrete(labels = c("Bowl" = "Bowl",
                              "VAC " = "Vacuum",
                              "VIS " = "Visual")) + labs(y = "Counts per 15 m")
p.sqb.1 <- p.sqb + theme(axis.title.x = element_blank(),axis.title.y = element_text(size = 10))
SQB.final.plot <- p.sqb.1 +
  scale_fill_viridis_d() + theme(legend.position = "none", plot.title = element_text(size = 10)) + ggti
SQB.final.plot
```

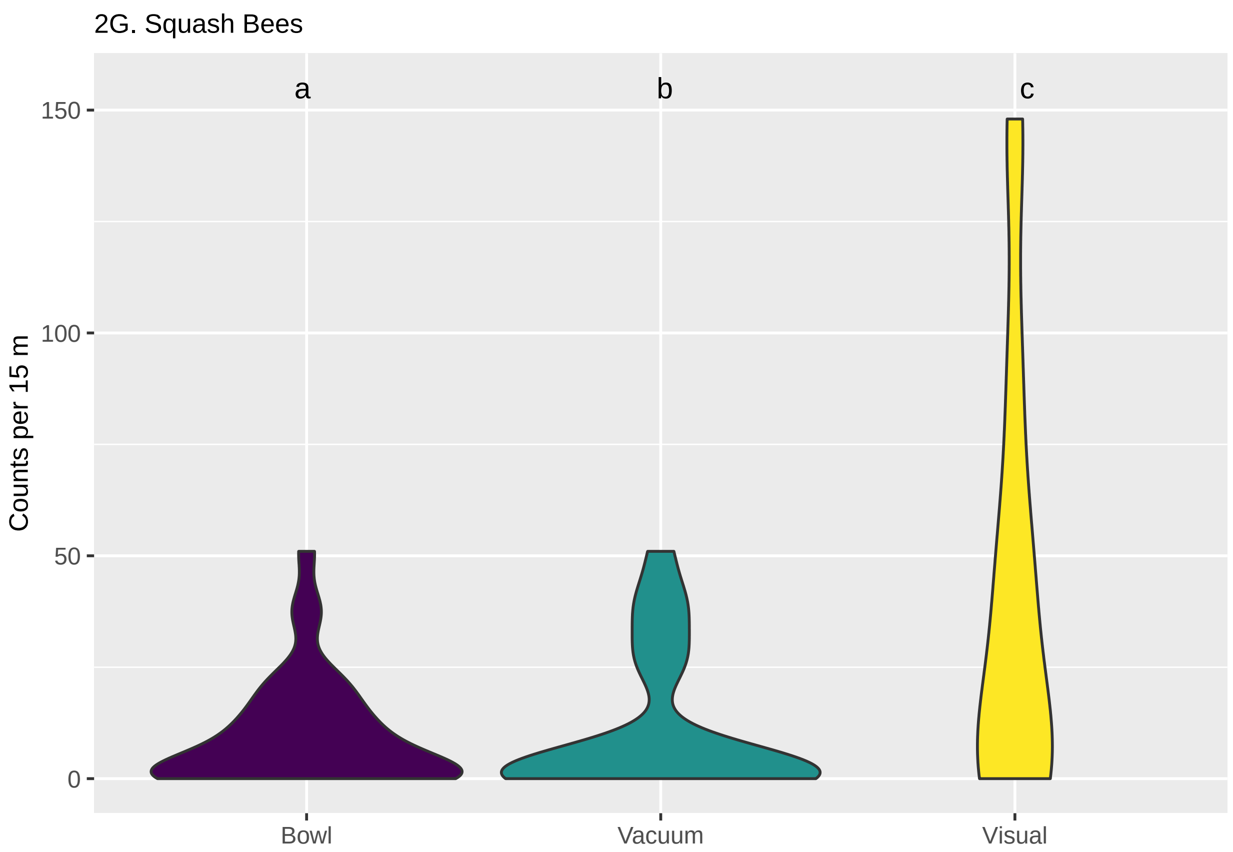

Small Black Bee

```
#checking for zero inflation
hist(Trap$SBB)
```

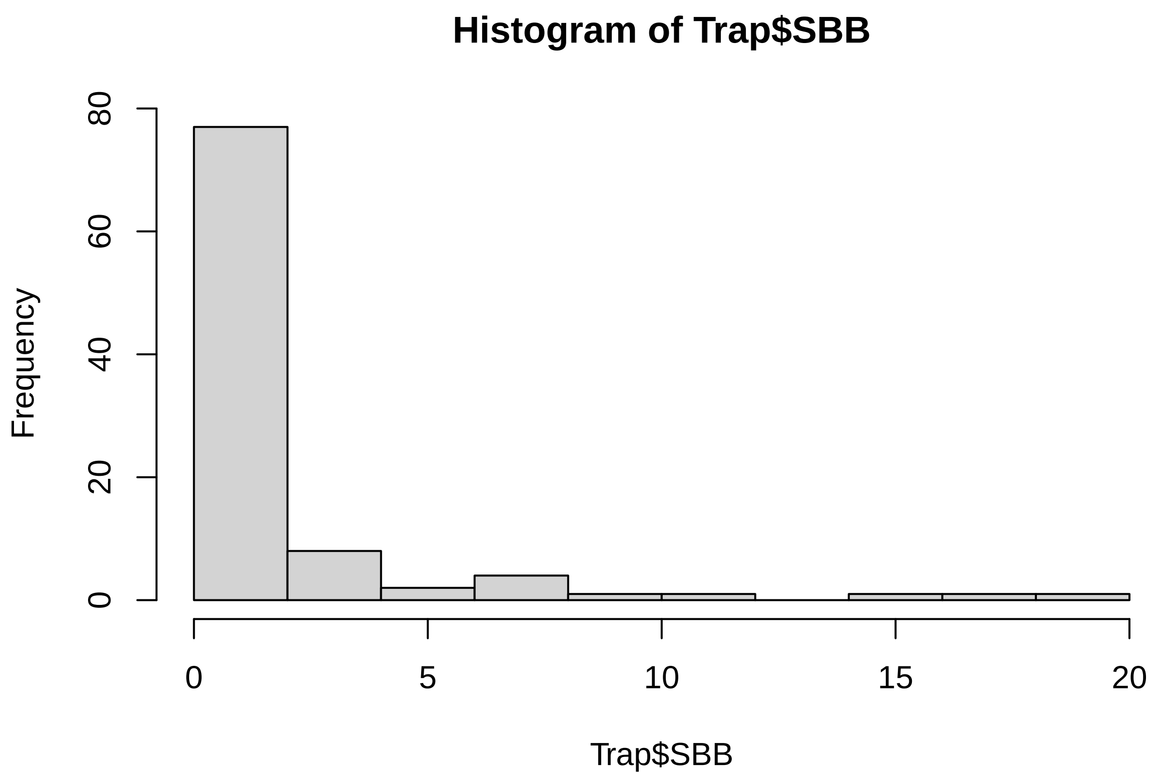


```
qqnorm(Trap$SBB)
```

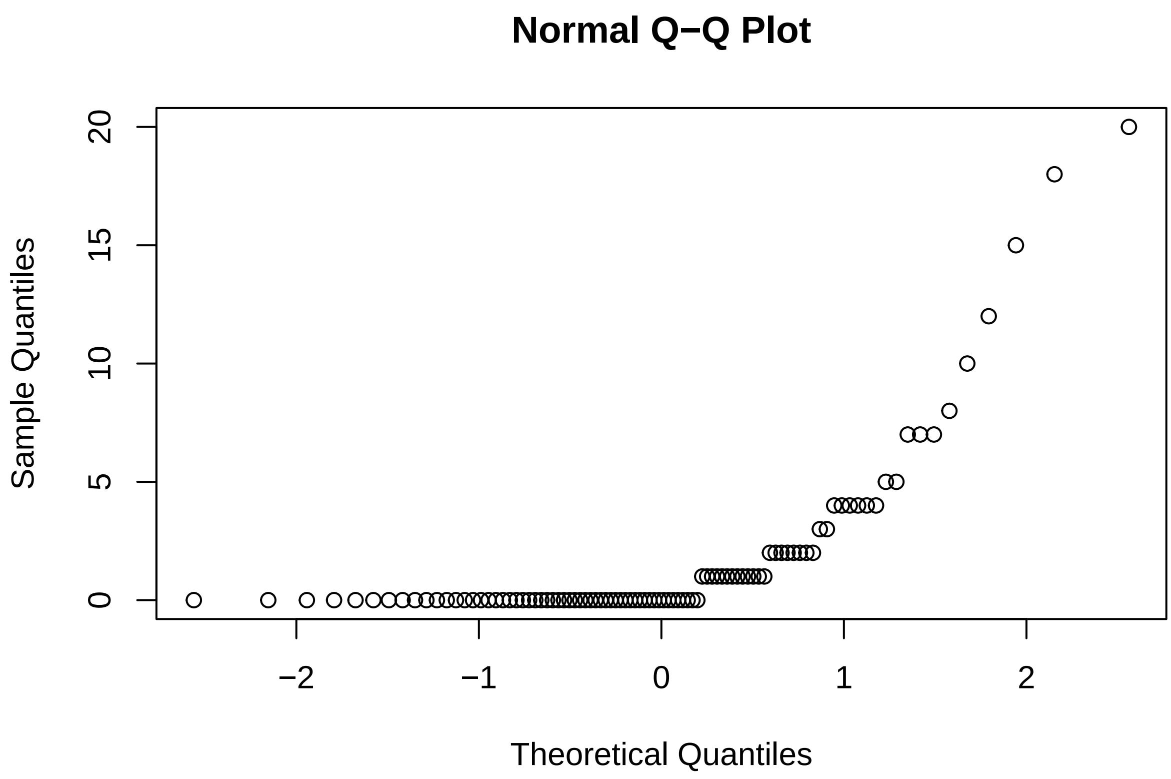


```r
#zero inflated glmm
model.SBB <- glmmTMB(SBB ~ TRAP + (1| LOC) + (1|DATE),
                     ziformula = ~1,
                     family = nbinom2(),
                     data = Trap)

summary(model.SBB)
```

```
##  Family: nbinom2  ( log )
## Formula:          SBB ~ TRAP + (1 | LOC) + (1 | DATE)
## Zero inflation:       ~1
## Data: Trap
##
##      AIC       BIC    logLik -2*log(L)  df.resid
##    263.3     281.3    -124.7     249.3        89
##
## Random effects:
##
## Conditional model:
##  Groups Name        Variance Std.Dev.
##  LOC    (Intercept) 0.545440 0.73854
##  DATE   (Intercept) 0.006215 0.07884
## Number of obs: 96, groups:  LOC, 3; DATE, 8
##
## Dispersion parameter for nbinom2 family (): 1.02
##
```

```
## Conditional model:
##              Estimate Std. Error z value Pr(>|z|)
## (Intercept)   -2.4132     0.8270  -2.918  0.00352 **
## TRAPBowl       4.4435     0.7676   5.789 7.09e-09 ***
## TRAPVAC        1.4199     0.7454   1.905  0.05681 .
## ---
## Signif. codes:  0 '***' 0.001 '**' 0.01 '*' 0.05 '.' 0.1 ' ' 1
##
## Zero-inflation model:
##              Estimate Std. Error z value Pr(>|z|)
## (Intercept)    -8.897   1003.434  -0.009    0.993
```

```
#Runs the DHARMa residuals to test distrbutions and model fits
sim_res.SBB <- simulateResiduals(fittedModel = model.SBB, plot = TRUE)
```

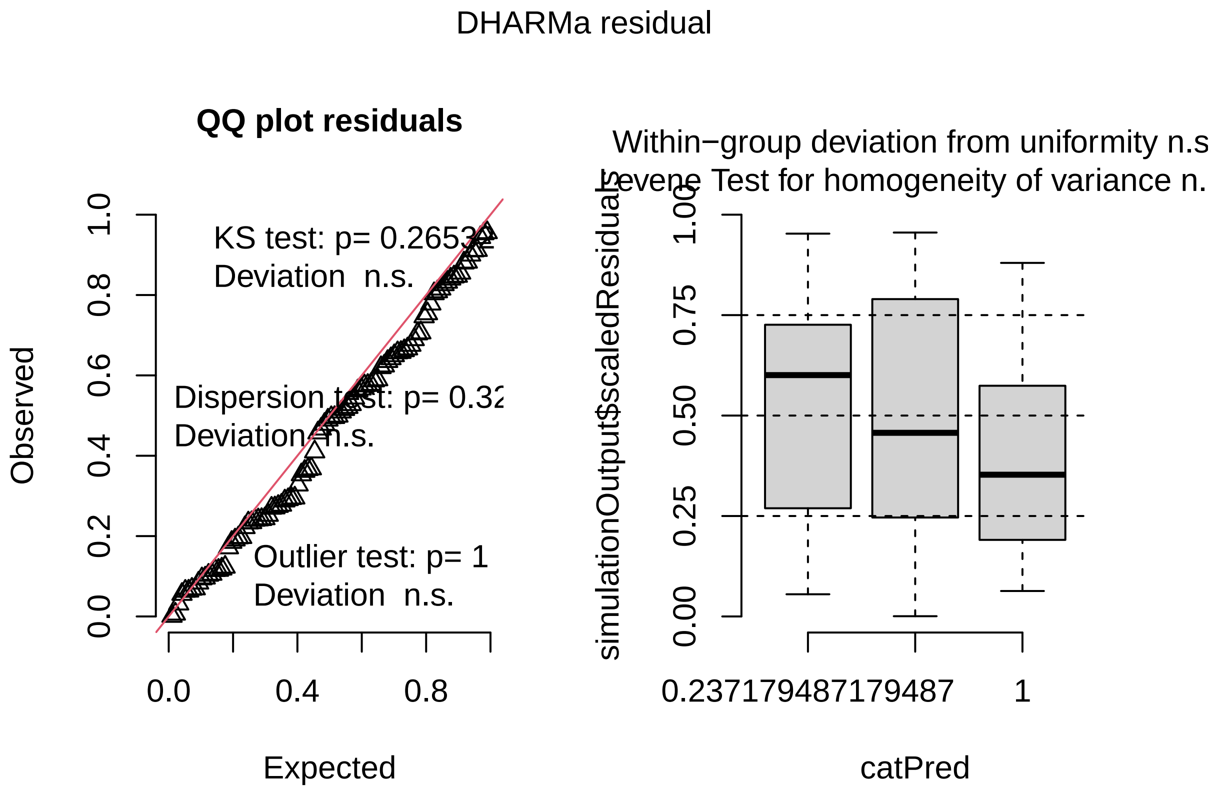


```
testResiduals(sim_res.SBB)
```

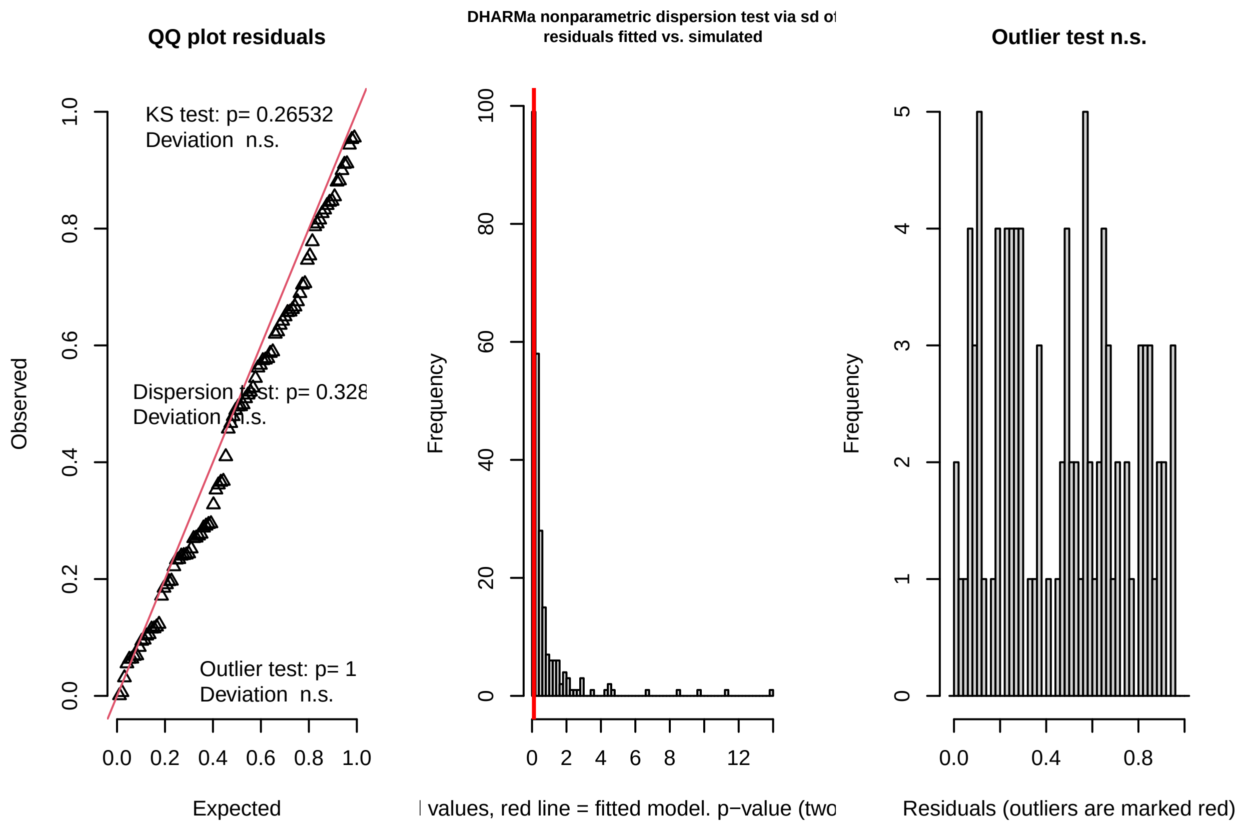


```
## $uniformity
##
##  Exact one-sample Kolmogorov-Smirnov test
##
## data:  simulationOutput$scaledResiduals
## D = 0.10079, p-value = 0.2653
## alternative hypothesis: two-sided
##
##
## $dispersion
##
##  DHARMa nonparametric dispersion test via sd of residuals fitted vs.
##  simulated
##
## data:  simulationOutput
## dispersion = 0.14655, p-value = 0.328
## alternative hypothesis: two.sided
##
##
## $outliers
##
##  DHARMa bootstrapped outlier test
##
## data:  simulationOutput
## outliers at both margin(s) = 0, observations = 96, p-value = 1
## alternative hypothesis: two.sided
```

```
##  percent confidence interval:
##  0.00000000 0.02630208
## sample estimates:
## outlier frequency (expected: 0.003125 )
##                                       0
```

```r
testDispersion(sim_res.SBB)
```

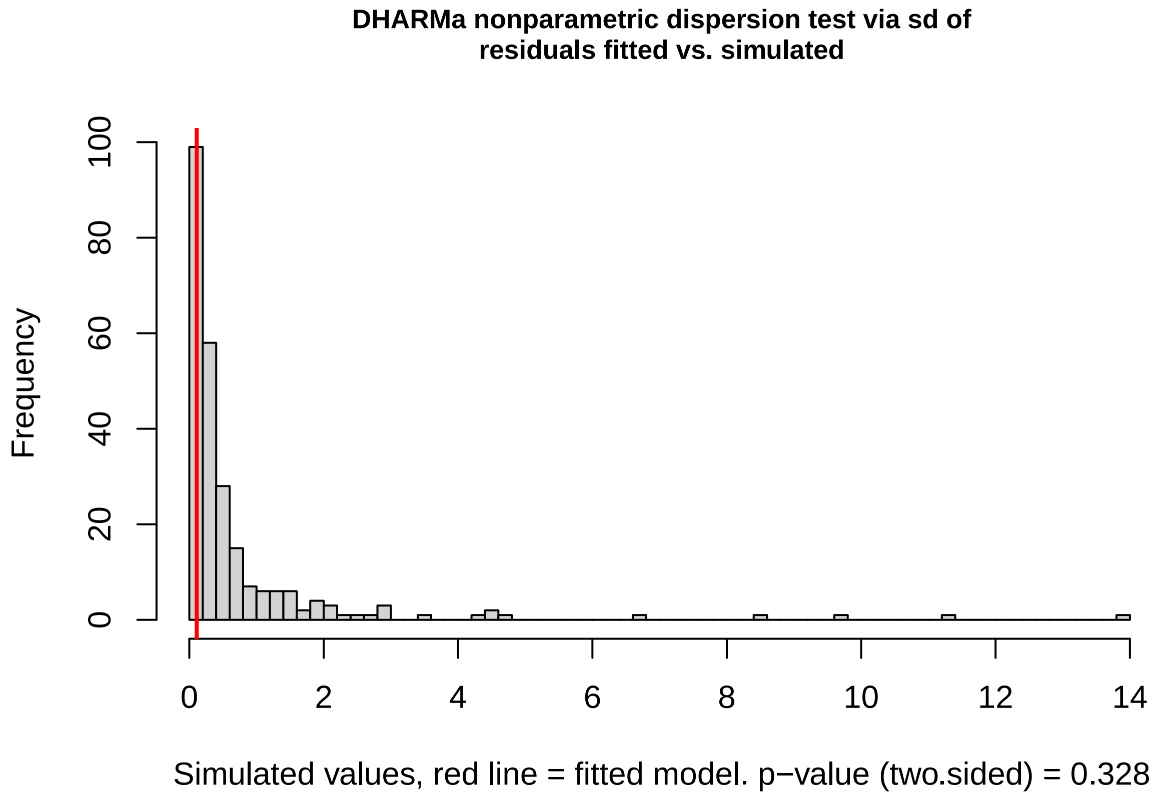


```
##
##  DHARMa nonparametric dispersion test via sd of residuals fitted vs.
##  simulated
##
## data:  simulationOutput
## dispersion = 0.14655, p-value = 0.328
## alternative hypothesis: two.sided
```

```r
testZeroInflation(sim_res.SBB)
```

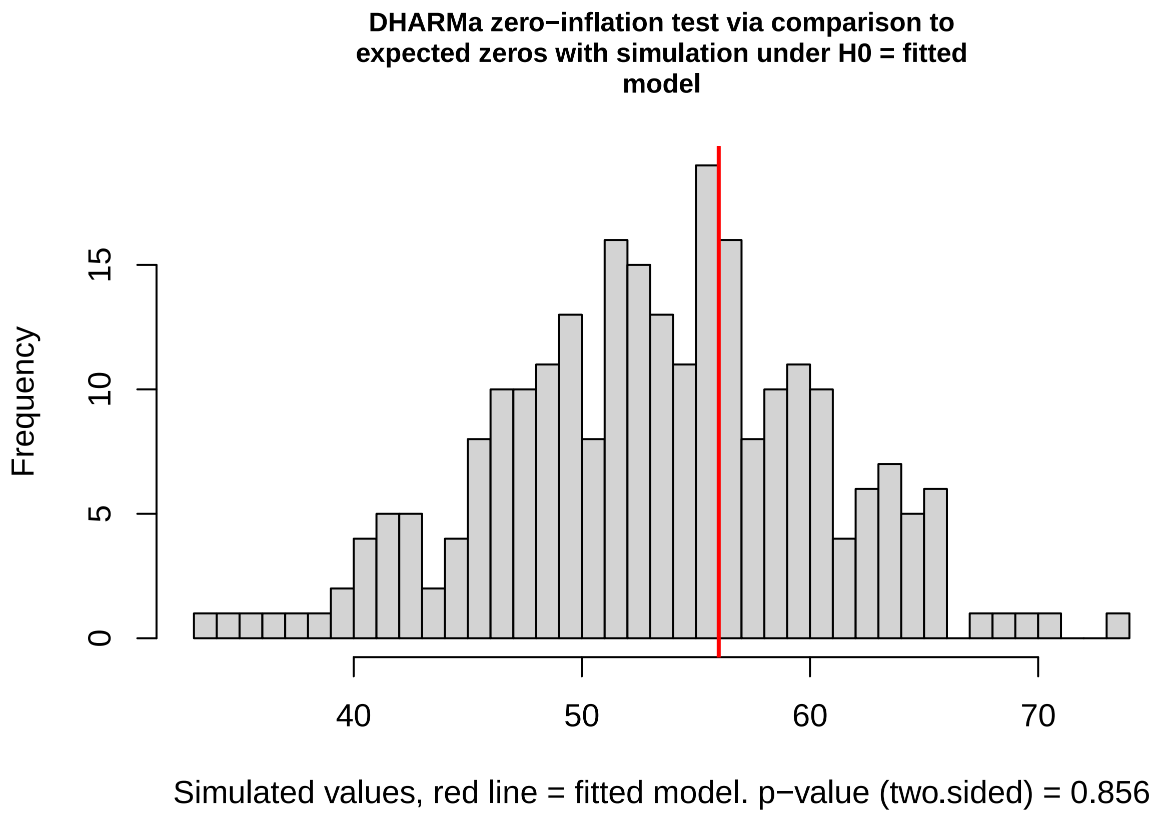


```
##
##  DHARMa zero-inflation test via comparison to expected zeros with
##  simulation under H0 = fitted model
##
## data:  simulationOutput
## ratioObsSim = 1.0408, p-value = 0.856
## alternative hypothesis: two.sided
```

```
# Reduced model for LRT
sbb_reduced <- glmmTMB(SBB ~ 1 + (1 | LOC) + (1 | DATE),
                       ziformula = ~1,
                       family = nbinom2(),
                       data = Trap)

# Likelihood ratio test
anova(model.SBB, sbb_reduced, test = "Chisq")
```

```
## Data: Trap
## Models:
## sbb_reduced: SBB ~ 1 + (1 | LOC) + (1 | DATE), zi=~1, disp=~1
## model.SBB: SBB ~ TRAP + (1 | LOC) + (1 | DATE), zi=~1, disp=~1
##             Df    AIC    BIC  logLik deviance  Chisq Chi Df Pr(>Chisq)
## sbb_reduced  5 323.87 336.69 -156.94   313.87
## model.SBB    7 263.34 281.29 -124.67   249.34 64.531      2  9.711e-15 ***
## ---
## Signif. codes:  0 '***' 0.001 '**' 0.01 '*' 0.05 '.' 0.1 ' ' 1
```

```r
#running emmeans with sidak
marginal.SBB = emmeans(model.SBB,
                       ~ TRAP, type = "response")
sbb.cld <- cld(marginal.SBB,
    alpha=0.05,
    Letters=letters,  ### Use lower-case letters for .group
    adjust="sidak")

# Clean up for plotting to add letters to plot
sbb.cld_result <- as.data.frame(sbb.cld)
sbb.cld_result$group <- as.character(sbb.cld_result$.group)

#PULL ONLY SBB for graphing
SBB.PLOT <- TRAP.1 %>% filter(MORPHO == 'SBB')
Count.SBB <- SBB.PLOT %>% select_("TRAP", "MORPHO", "COUNT")
```

```
## Warning: `select_()` was deprecated in dplyr 0.7.0.
## i Please use `select()` instead.
## Call `lifecycle::last_lifecycle_warnings()` to see where this warning was
## generated.
```

```r
# plotting the SQB Violin Graph
p.sbb <-ggplot(SBB.PLOT, aes(x=TRAP, y=COUNT, fill=TRAP)) +
  geom_violin() + scale_x_discrete(labels = c("Bowl" = "Bowl",
                              "VAC " = "Vacuum",
                              "VIS " = "Visual"))  + labs(y = "Counts per 15 m")
p.sbb.1 <- p.sbb + theme(axis.title.x=element_blank(),axis.title.y = element_text(size = 10))
SBB.final.plot <- p.sbb.1 +
  scale_fill_viridis_d() + theme(legend.position = "none", plot.title = element_text(size = 10)) + ggti

SBB.final.plot
```

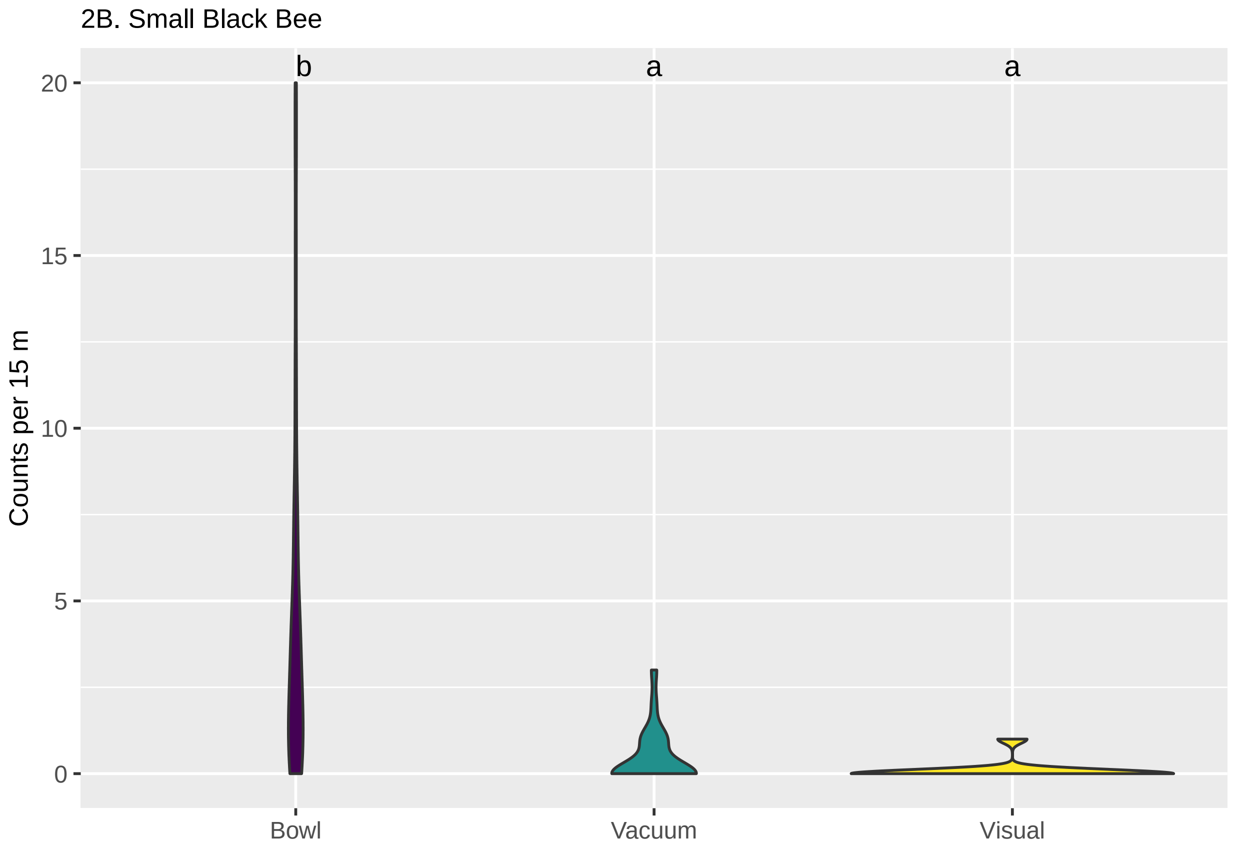

Large Black Bee

```
#checking for zero inflation
hist(Trap$LBB)
```

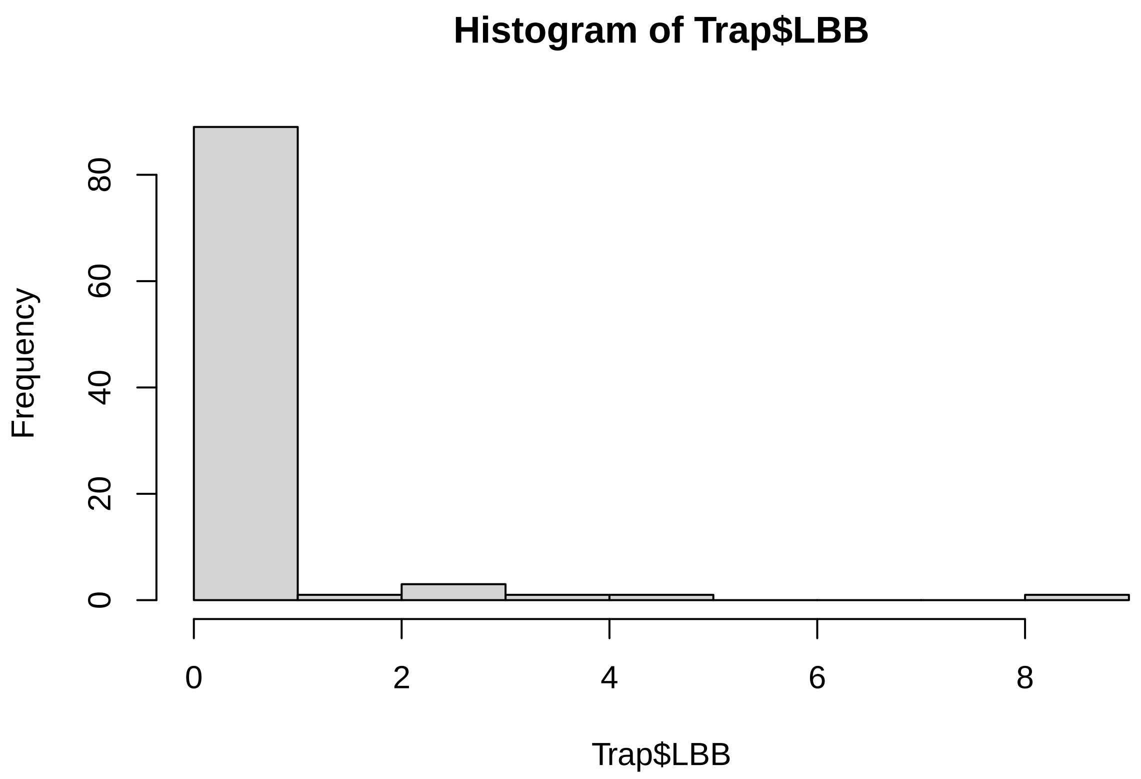


```
qqnorm(Trap$LBB)
```

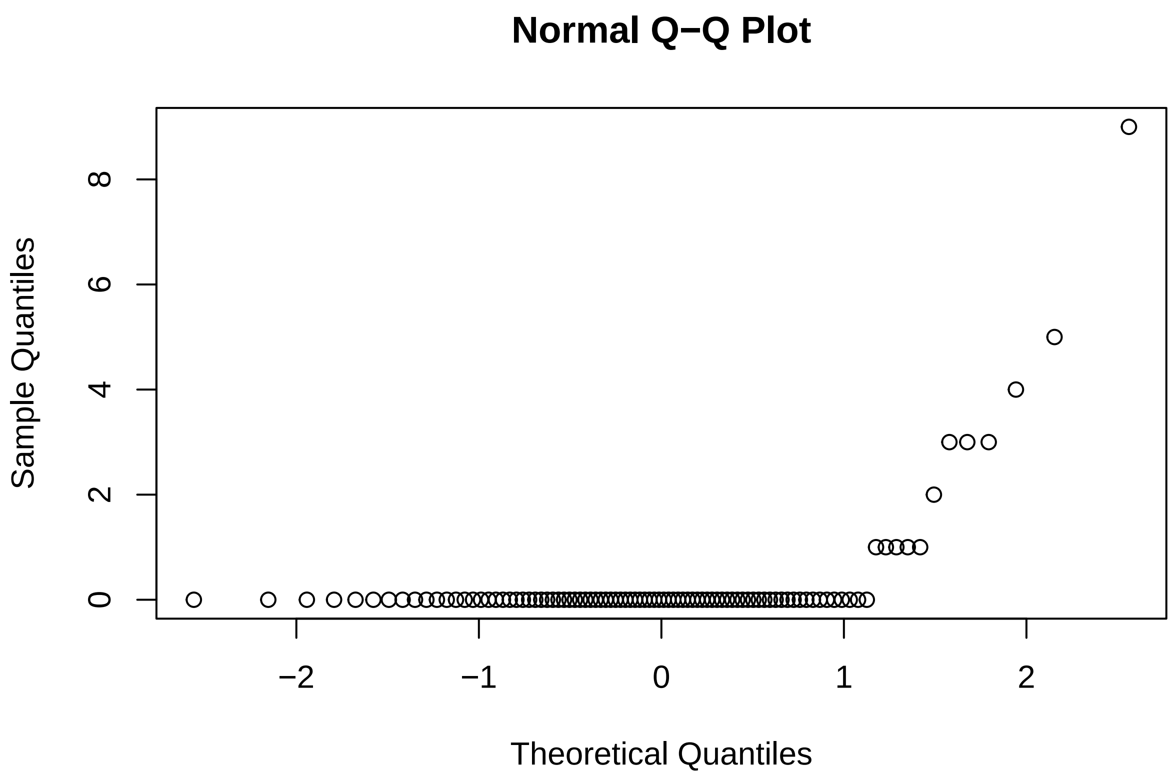


```
#zero inflated glmm
model.LBB <- glmmTMB(LBB ~ TRAP  + (1 | DATE),
                     ziformula = ~1,
                     family = poisson,
                     data = Trap.novac)

summary(model.LBB)
```

```
##  Family: poisson  ( log )
## Formula:          LBB ~ TRAP + (1 | DATE)
## Zero inflation:       ~1
## Data: Trap.novac
##
##      AIC      BIC   logLik -2*log(L)  df.resid
##    106.4    115.6    -49.2      98.4        69
##
## Random effects:
##
## Conditional model:
##  Groups Name        Variance Std.Dev.
##  DATE   (Intercept) 0.3876   0.6225
## Number of obs: 73, groups:  DATE, 8
##
## Conditional model:
##              Estimate Std. Error z value Pr(>|z|)
## (Intercept)  -1.4343     0.8075  -1.776  0.07569 .
```

```
## TRAPBowl       2.4018     0.7708   3.116  0.00183 **
## ---
## Signif. codes:  0 '***' 0.001 '**' 0.01 '*' 0.05 '.' 0.1 ' ' 1
##
## Zero-inflation model:
##             Estimate Std. Error z value Pr(>|z|)
## (Intercept)   0.9127     0.4088   2.233   0.0256 *
## ---
## Signif. codes:  0 '***' 0.001 '**' 0.01 '*' 0.05 '.' 0.1 ' ' 1
```

```
#Runs the DHARMa residuals to test distrbutions and model fits
sim_res.LBB <- simulateResiduals(fittedModel = model.LBB, plot = TRUE)
```

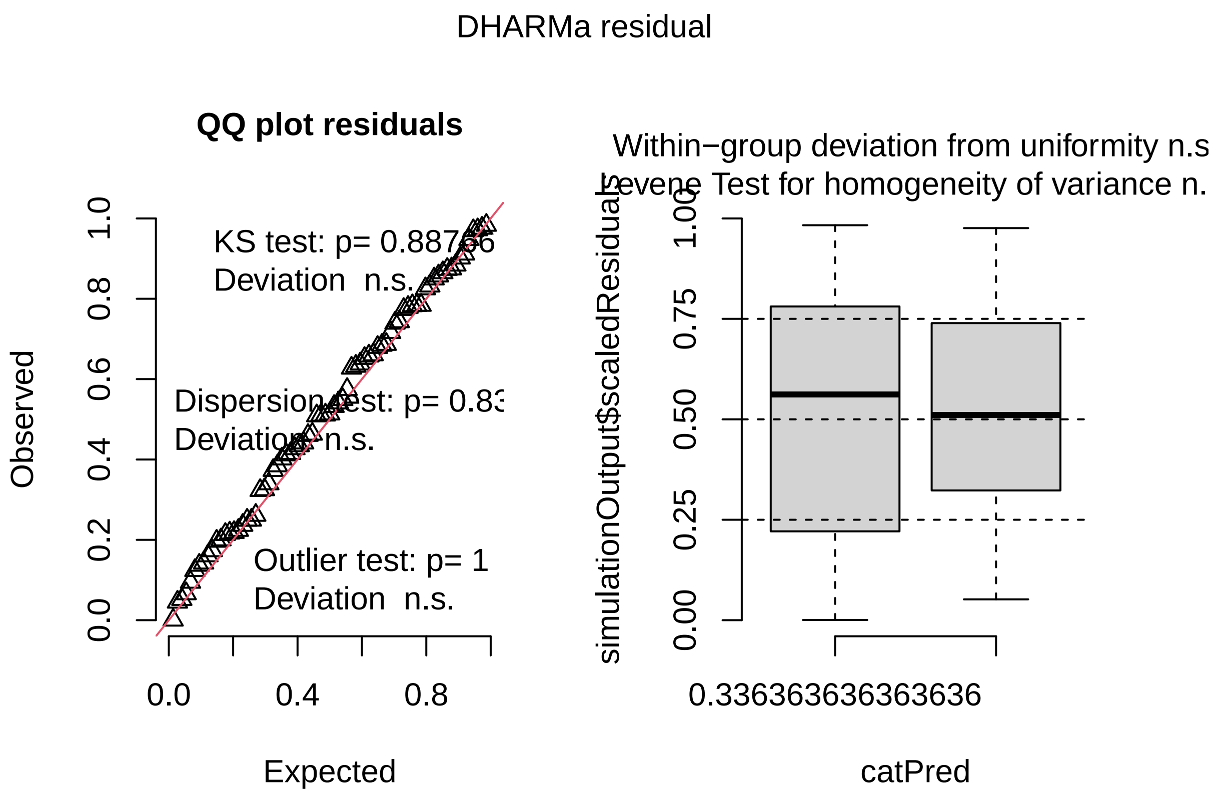


```
testResiduals(sim_res.LBB)
```

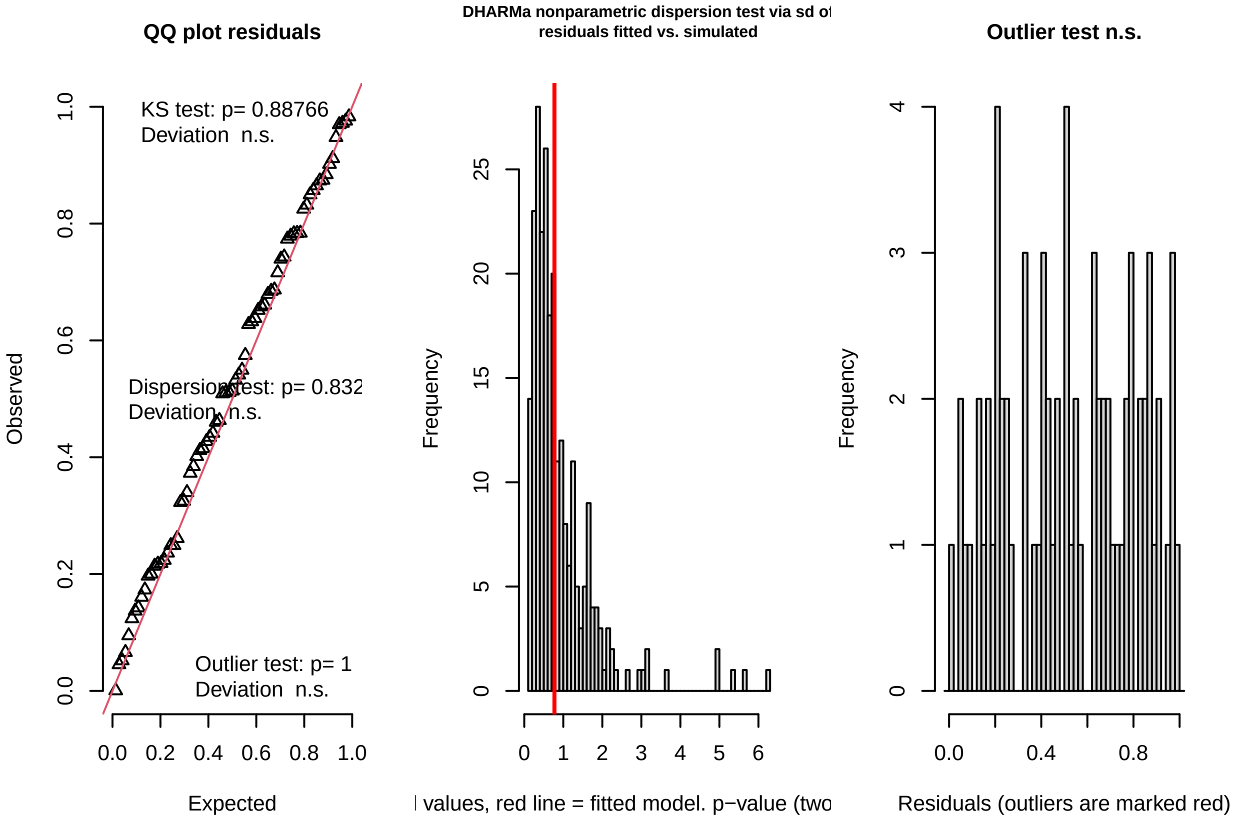


```
## $uniformity
##
##  Exact one-sample Kolmogorov-Smirnov test
##
## data:  simulationOutput$scaledResiduals
## D = 0.065961, p-value = 0.8877
## alternative hypothesis: two-sided
##
##
## $dispersion
##
##  DHARMa nonparametric dispersion test via sd of residuals fitted vs.
##  simulated
##
## data:  simulationOutput
## dispersion = 0.82943, p-value = 0.832
## alternative hypothesis: two.sided
##
##
## $outliers
##
##  DHARMa bootstrapped outlier test
##
## data:  simulationOutput
## outliers at both margin(s) = 0, observations = 73, p-value = 1
## alternative hypothesis: two.sided
```

```
##  percent confidence interval:
##  0.00000000 0.02739726
## sample estimates:
## outlier frequency (expected: 0.00383561643835616 )
##                                                  0
```

```r
testDispersion(sim_res.LBB)
```

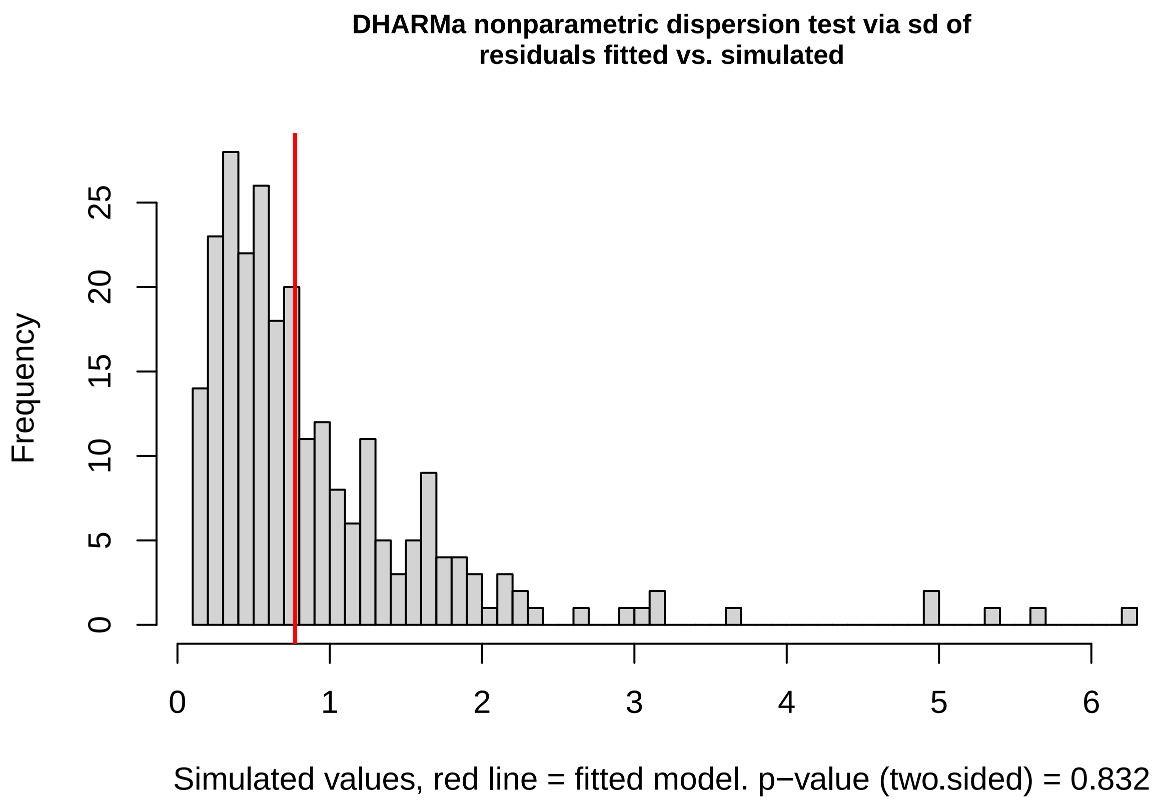


```
##
##  DHARMa nonparametric dispersion test via sd of residuals fitted vs.
##  simulated
##
## data:  simulationOutput
## dispersion = 0.82943, p-value = 0.832
## alternative hypothesis: two.sided
```

```r
testZeroInflation(sim_res.LBB)
```

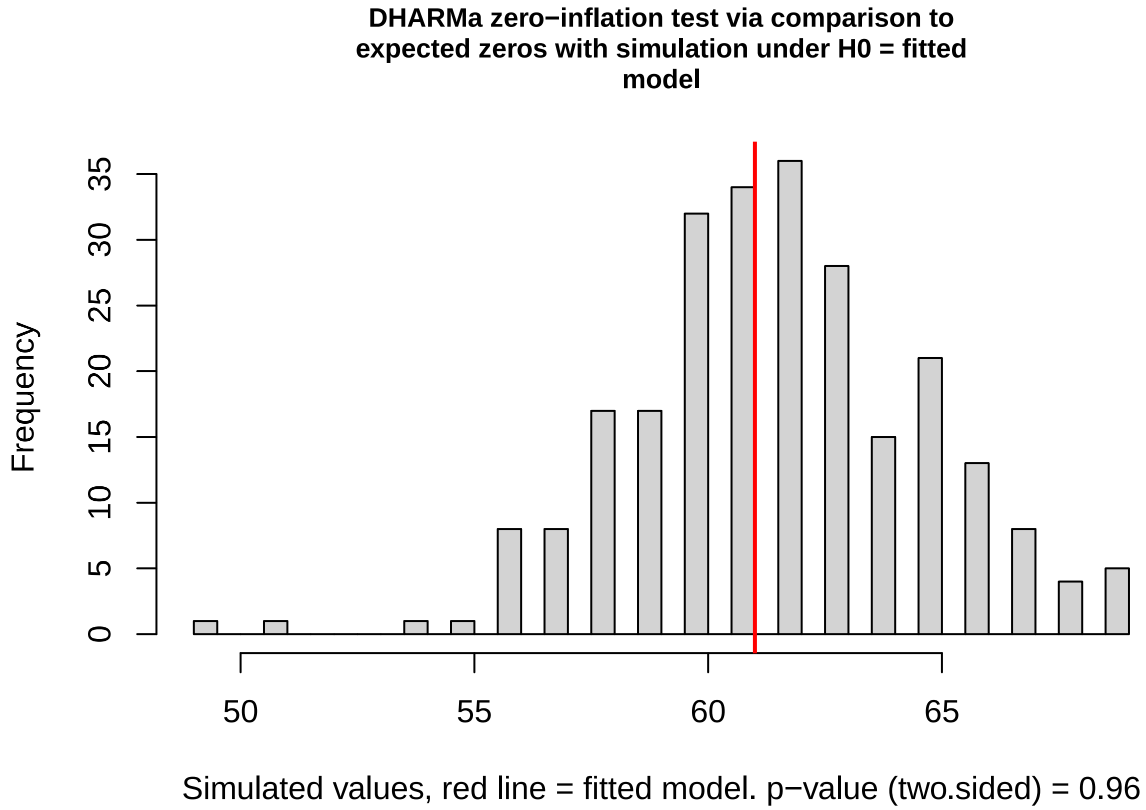


```
##
##  DHARMa zero-inflation test via comparison to expected zeros with
##  simulation under H0 = fitted model
##
## data:  simulationOutput
## ratioObsSim = 0.98846, p-value = 0.96
## alternative hypothesis: two.sided
```

```
# Reduced model for LRT
lbb_reduced <- glmmTMB(LBB ~ 1 + (1 | DATE),
                      ziformula = ~1,
                      family = poisson,
                      data = Trap.novac)

# Likelihood ratio test
anova(model.LBB, lbb_reduced, test = "Chisq")
```

```
## Data: Trap.novac
## Models:
## lbb_reduced: LBB ~ 1 + (1 | DATE), zi=~1, disp=~1
## model.LBB: LBB ~ TRAP + (1 | DATE), zi=~1, disp=~1
##             Df    AIC    BIC  logLik deviance  Chisq Chi Df Pr(>Chisq)
## lbb_reduced  3 112.02 118.89 -53.008  106.016
## model.LBB    4 106.41 115.57 -49.203   98.406 7.6103      1   0.005803 **
## ---
## Signif. codes:  0 '***' 0.001 '**' 0.01 '*' 0.05 '.' 0.1 ' ' 1
```

```
#running emmeans with sidak
marginal.LBB = emmeans(model.LBB, ~ TRAP, type = "response")
lbb.cld <- cld(marginal.LBB,
    alpha=0.05,
    Letters=letters,  ### Use lower-case letters for .group
    adjust="sidak")

# Clean up for plotting to add letters to plot
lbb.cld_result <- as.data.frame(lbb.cld)
lbb.cld_result$group <- as.character(lbb.cld_result$.group)


#PULL ONLY LBB for graphing
LBB.PLOT <- TRAP.1 %>% filter(MORPHO == 'LBB')
Count.LBB <- LBB.PLOT %>% select_("TRAP", "MORPHO", "COUNT")
```

```
## Warning: `select_()` was deprecated in dplyr 0.7.0.
## i Please use `select()` instead.
## Call `lifecycle::last_lifecycle_warnings()` to see where this warning was
## generated.
```

```
# LBB Basic violin plot
p.lbb <-ggplot(LBB.PLOT, aes(x=TRAP, y=COUNT, fill=TRAP)) +
  geom_violin() + scale_x_discrete(labels = c("Bowl" = "Bowl",
                              "VAC " = "Vacuum",
                              "VIS " = "Visual"))  + labs(y = "Counts per 15 m")
p.lbb.1 <- p.lbb + theme(axis.title.x=element_blank(),axis.title.y = element_text(size = 10))

LBB.final.plot <- p.lbb.1 +
  scale_fill_viridis_d() + theme(legend.position = "none", plot.title = element_text(size = 10)) + ggti

LBB.final.plot
```

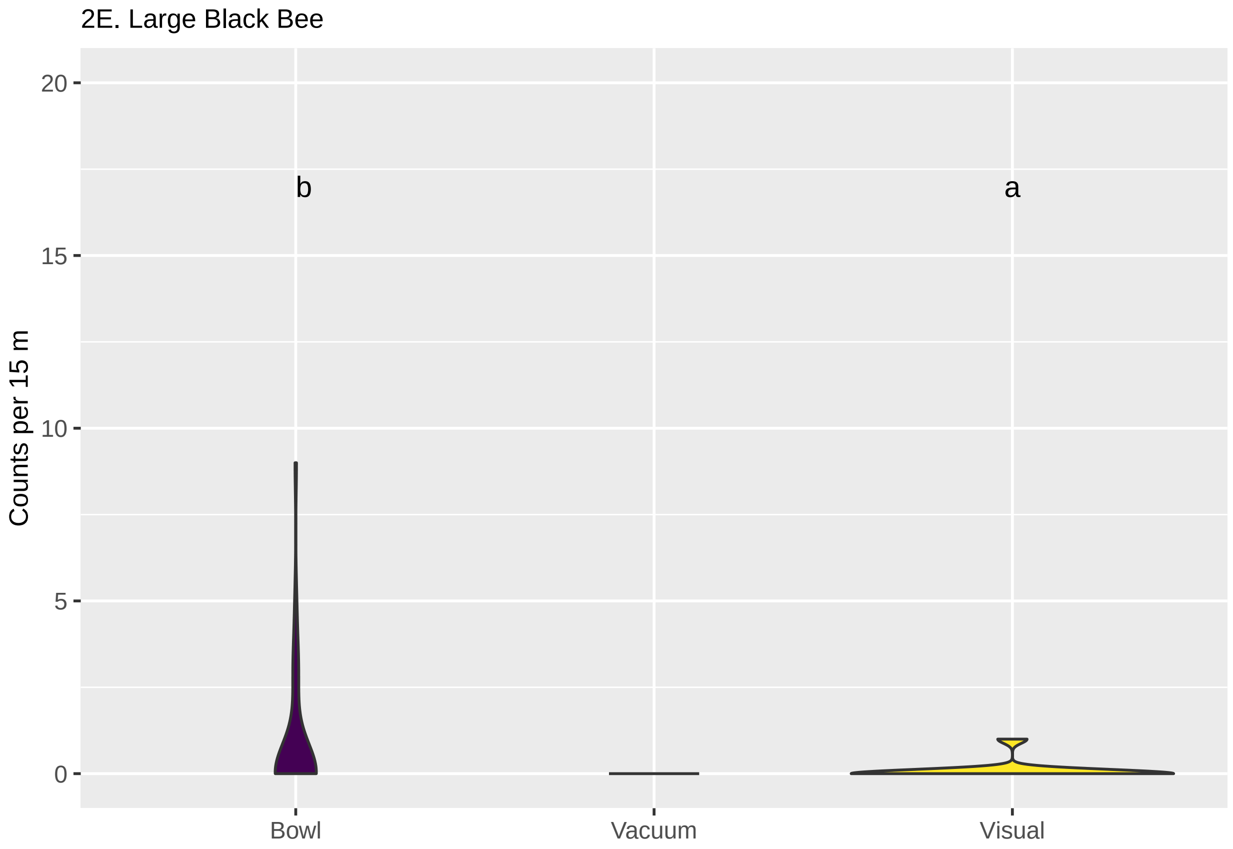

Small Striped Bee

```
#checking for zero inflation
hist(Trap$SSB)
```

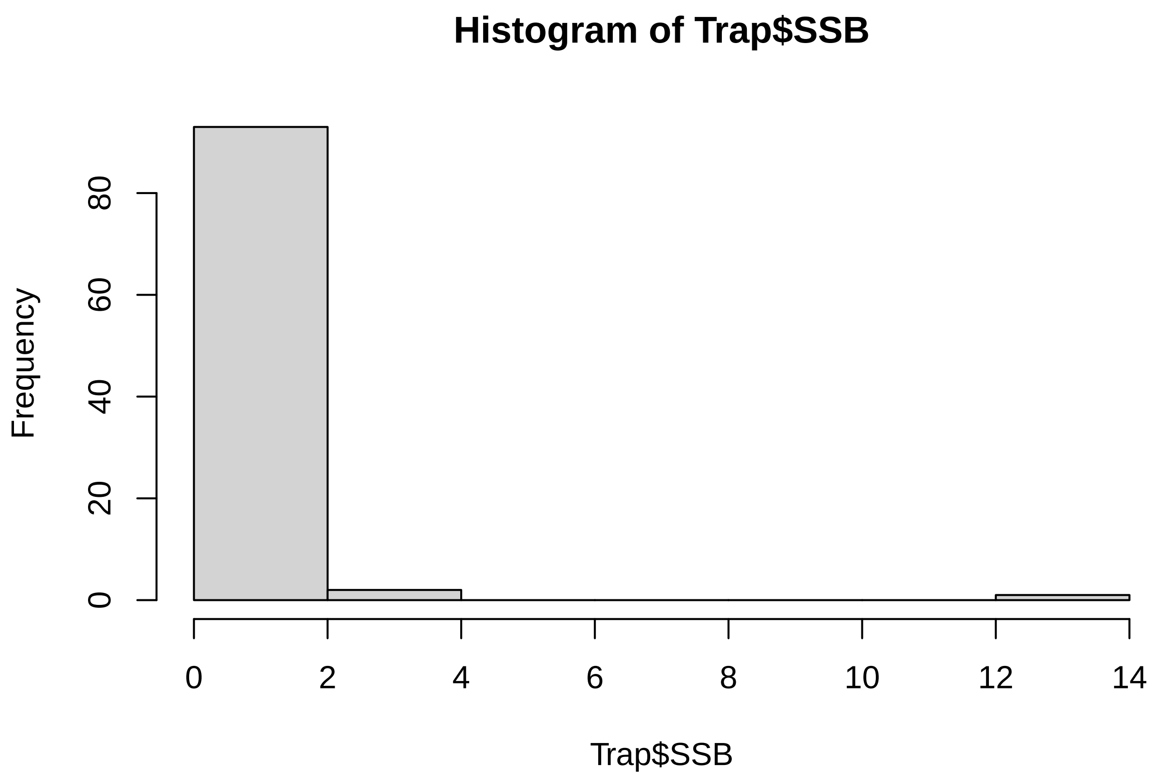


```
qqnorm(Trap$SSB)
```

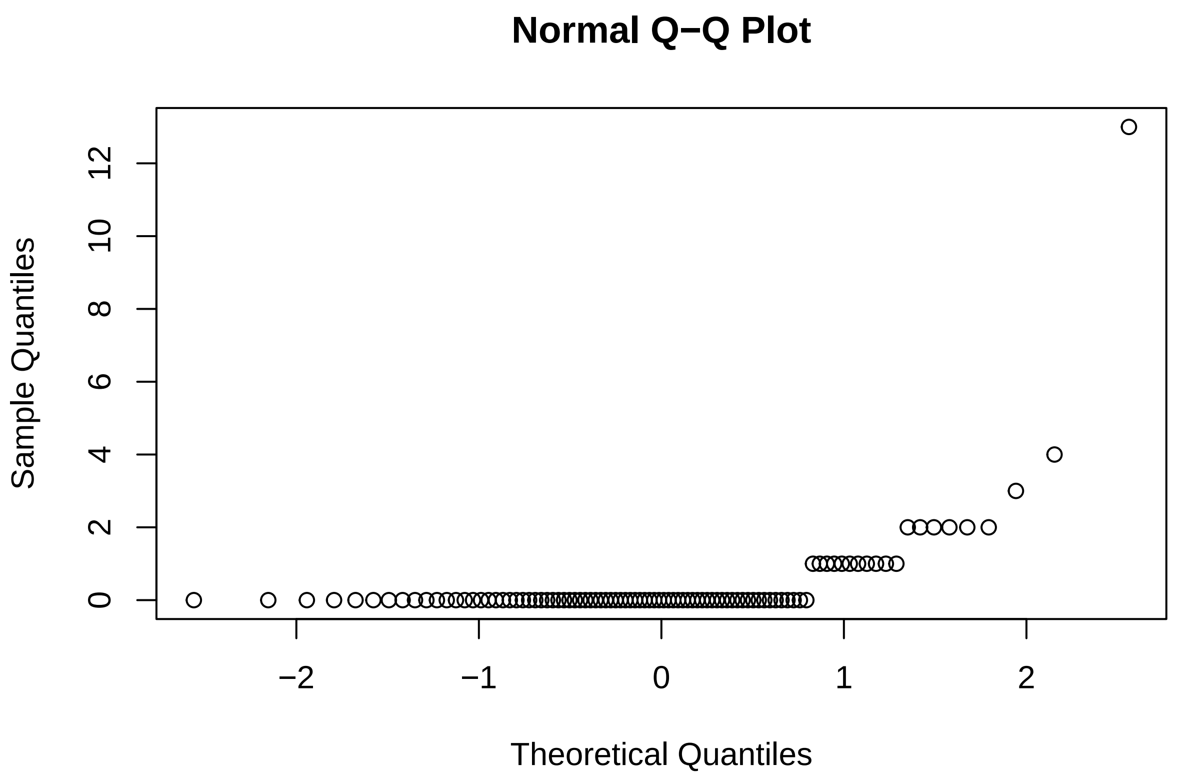


```r
#zero inflated glmm
model.SSB <- glmmTMB(SSB ~ TRAP + (1 | LOC) + (1 | DATE),
                      ziformula = ~1,
                      family = poisson(),
                      data = Trap.novac)

summary(model.SSB)
```

```
##  Family: poisson  ( log )
## Formula:          SSB ~ TRAP + (1 | LOC) + (1 | DATE)
## Zero inflation:       ~1
## Data: Trap.novac
##
##      AIC       BIC    logLik -2*log(L)  df.resid
##    134.9     146.3     -62.4     124.9        68
##
## Random effects:
##
## Conditional model:
##  Groups Name        Variance  Std.Dev.
##  LOC    (Intercept) 6.277e-09 7.923e-05
##  DATE   (Intercept) 1.239e+00 1.113e+00
## Number of obs: 73, groups:  LOC, 3; DATE, 8
##
## Conditional model:
##             Estimate Std. Error z value Pr(>|z|)
```

```
## (Intercept)  -2.3574     0.7346  -3.209  0.00133 **
## TRAPBowl       2.3866     0.5812   4.106 4.02e-05 ***
## ---
## Signif. codes:  0 '***' 0.001 '**' 0.01 '*' 0.05 '.' 0.1 ' ' 1
##
## Zero-inflation model:
##             Estimate Std. Error z value Pr(>|z|)
## (Intercept)  -1.2591     0.8304  -1.516    0.129
```

```
#Runs the DHARMa residuals to test distrbutions and model fits
sim_res.SSB <- simulateResiduals(fittedModel = model.SSB, plot = TRUE)
```

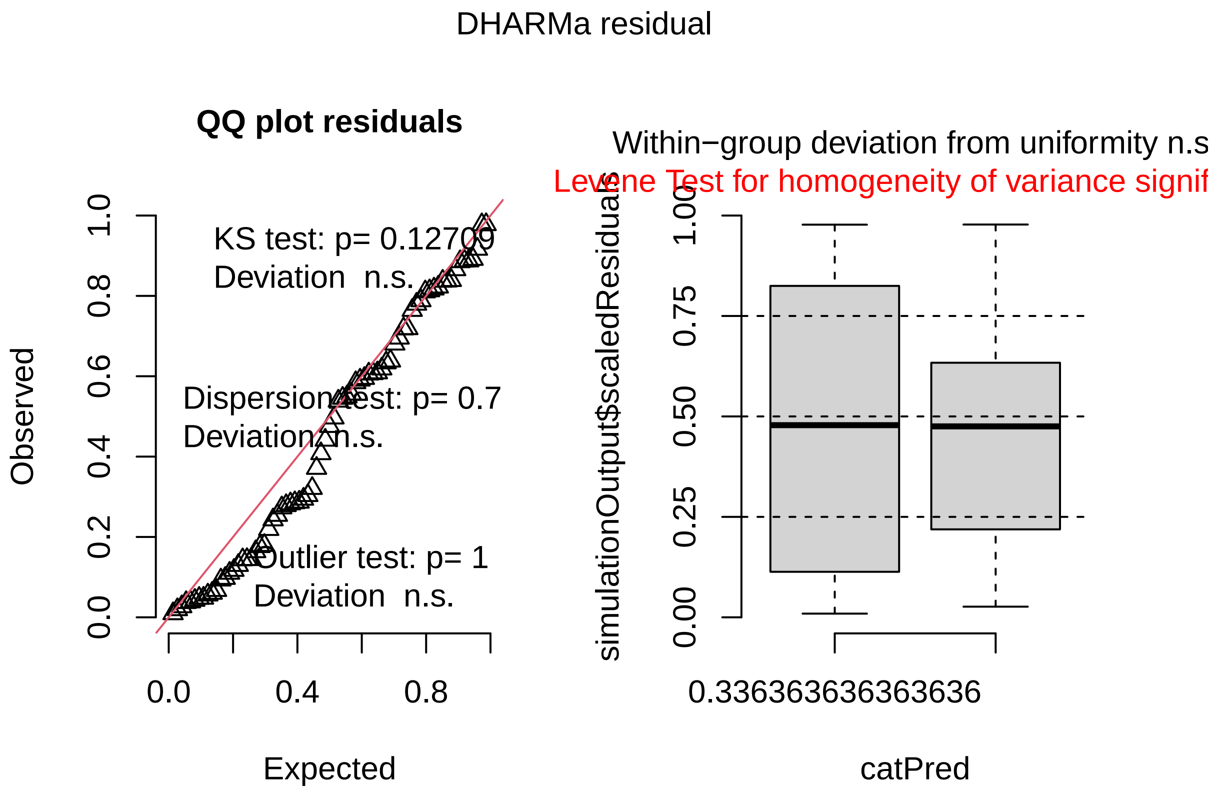


```
testResiduals(sim_res.SSB)
```

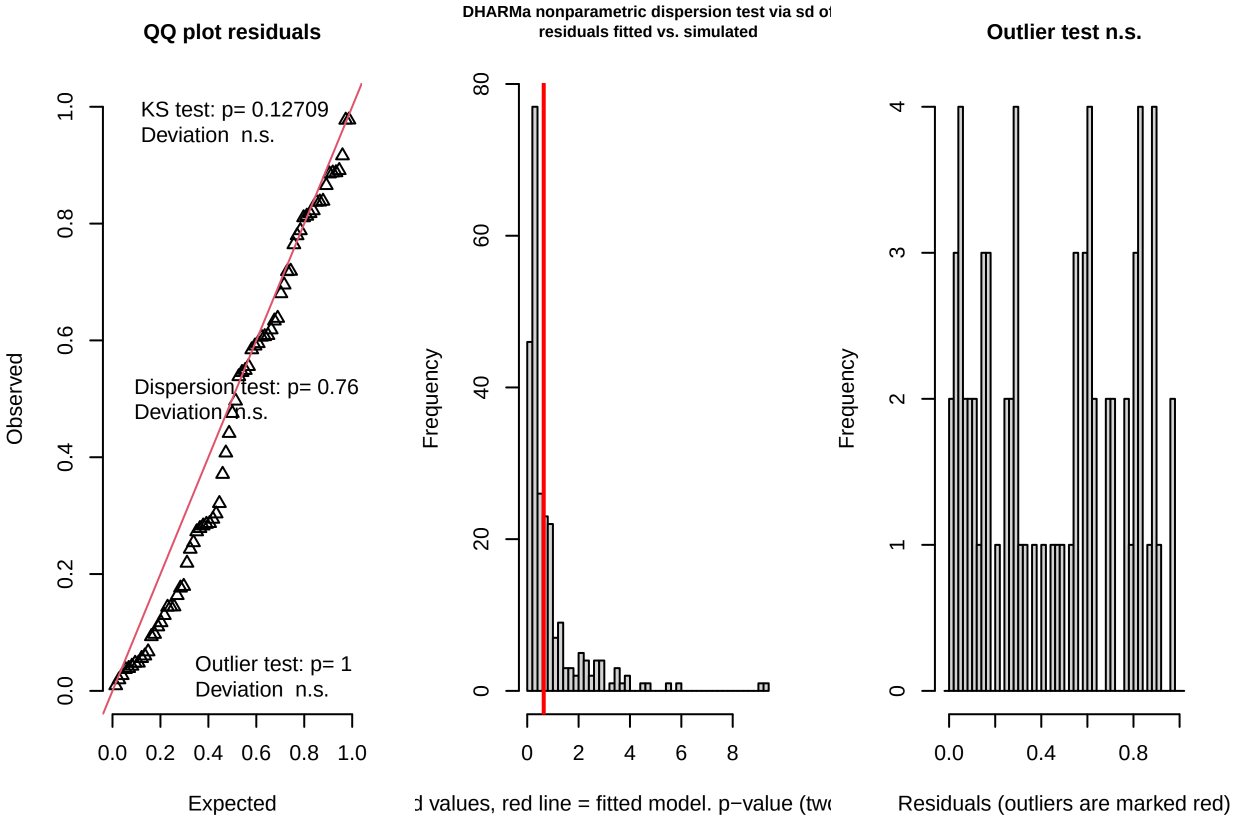


```
## $uniformity
##
##  Exact one-sample Kolmogorov-Smirnov test
##
## data:  simulationOutput$scaledResiduals
## D = 0.13505, p-value = 0.1271
## alternative hypothesis: two-sided
##
##
## $dispersion
##
##  DHARMa nonparametric dispersion test via sd of residuals fitted vs.
##  simulated
##
## data:  simulationOutput
## dispersion = 0.72733, p-value = 0.76
## alternative hypothesis: two.sided
##
##
## $outliers
##
##  DHARMa bootstrapped outlier test
##
## data:  simulationOutput
## outliers at both margin(s) = 0, observations = 73, p-value = 1
## alternative hypothesis: two.sided
```

```
##  percent confidence interval:
##  0.00000000 0.02739726
## sample estimates:
## outlier frequency (expected: 0.00273972602739726 )
##                                                  0
```

```
testDispersion(sim_res.SSB)
```

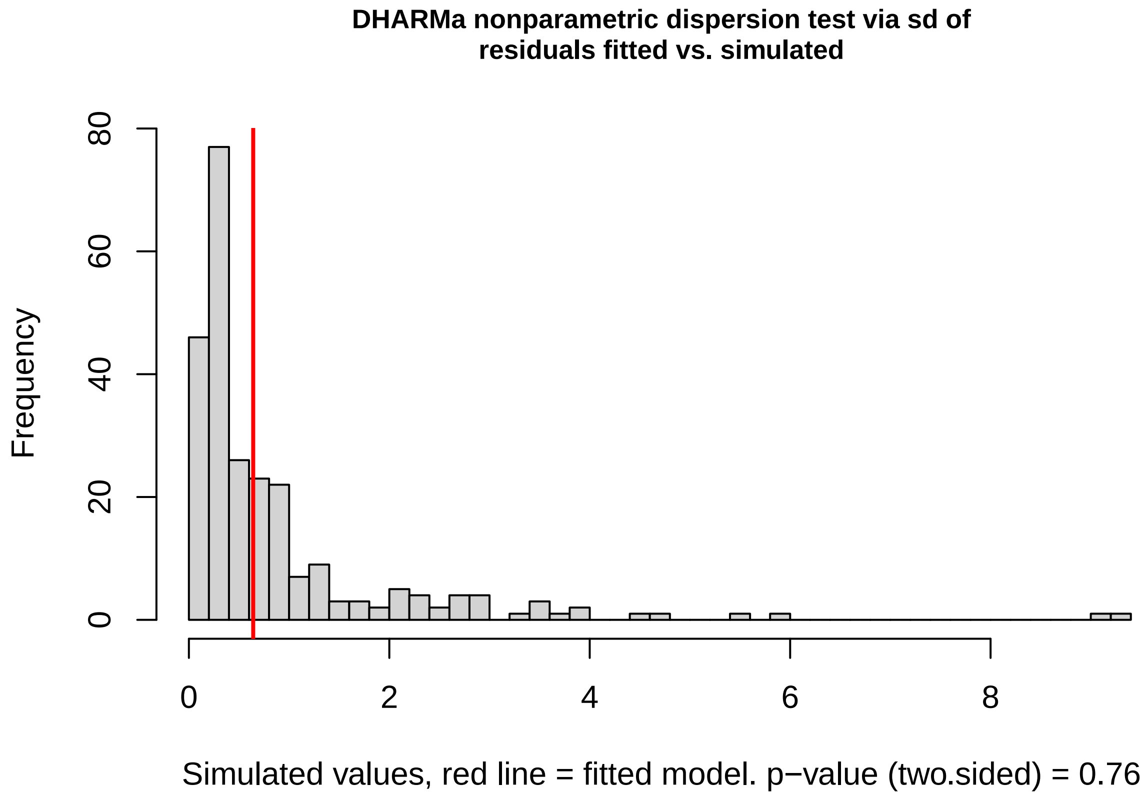


```
##
##  DHARMa nonparametric dispersion test via sd of residuals fitted vs.
##  simulated
##
## data:  simulationOutput
## dispersion = 0.72733, p-value = 0.76
## alternative hypothesis: two.sided
```

```
testZeroInflation(sim_res.SSB)
```

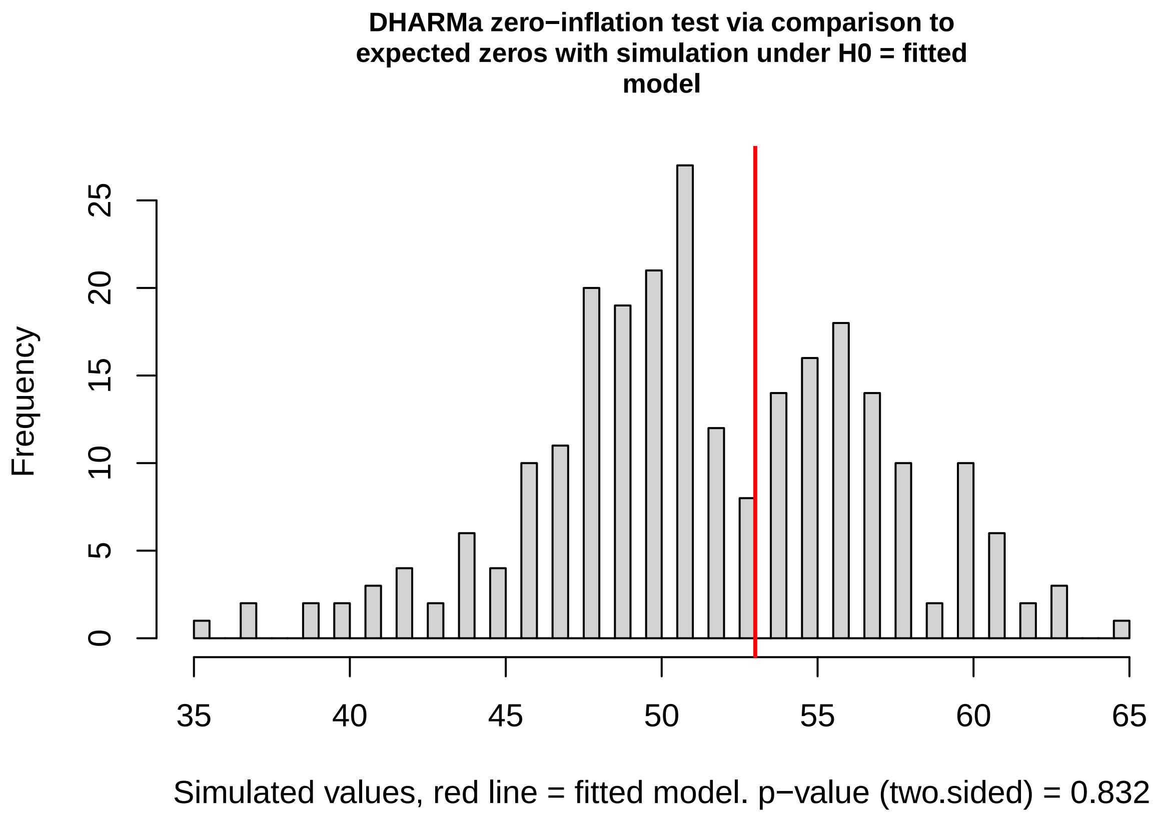


```
##
##  DHARMa zero-inflation test via comparison to expected zeros with
##  simulation under H0 = fitted model
##
## data:  simulationOutput
## ratioObsSim = 1.0259, p-value = 0.832
## alternative hypothesis: two.sided
```

```
# Reduced model for LRT
ssb_reduced <- glmmTMB(SSB ~ 1 + (1 | LOC) + (1 | DATE),
                      ziformula = ~1,
                      family = poisson(),
                      data = Trap.novac)

# Likelihood ratio test
anova(model.SSB,ssb_reduced, test = "Chisq")
```

```
## Data: Trap.novac
## Models:
## ssb_reduced: SSB ~ 1 + (1 | LOC) + (1 | DATE), zi=~1, disp=~1
## model.SSB: SSB ~ TRAP + (1 | LOC) + (1 | DATE), zi=~1, disp=~1
##             Df    AIC    BIC  logLik deviance  Chisq Chi Df Pr(>Chisq)
## ssb_reduced  4 150.62 159.78 -71.309   142.62
## model.SSB    5 134.87 146.32 -62.434   124.87 17.751      1  2.518e-05 ***
## ---
## Signif. codes:  0 '***' 0.001 '**' 0.01 '*' 0.05 '.' 0.1 ' ' 1
```

```
#running emmeans with sidak
marginal.SSB = emmeans(model.SSB,
                   ~ TRAP, type = "response")
cld.ssb <- cld(marginal.SSB,
    alpha=0.05,
    Letters=letters,  ### Use lower-case letters for .group
    adjust="sidak")

# Clean up for plotting to add letters to plot
ssb.cld_result <- as.data.frame(cld.ssb)
ssb.cld_result$group <- as.character(ssb.cld_result$.group)

#PULL ONLY SSB for graphing
SSB.PLOT <- TRAP.1 %>% filter(MORPHO == 'SSB')
Count.SSB <- SSB.PLOT %>% select_("TRAP", "MORPHO", "COUNT")
```

```
## Warning: `select_()` was deprecated in dplyr 0.7.0.
## i Please use `select()` instead.
## Call `lifecycle::last_lifecycle_warnings()` to see where this warning was
## generated.
```

```
# SSB Basic violin plot
p.ssb <-ggplot(SSB.PLOT, aes(x=TRAP, y=COUNT, fill=TRAP)) +
  geom_violin() + scale_x_discrete(labels = c("Bowl" = "Bowl",
                              "VAC " = "Vacuum",
                              "VIS " = "Visual"))  + labs(y = "Counts per 15 m")
p.ssb.1 <- p.ssb + theme(axis.title.x=element_blank(),axis.title.y = element_text(size = 10))
SSB.final.plot <- p.ssb.1 +
  scale_fill_viridis_d() + theme(legend.position = "none", plot.title = element_text(size = 10)) + ggti
SSB.final.plot
```

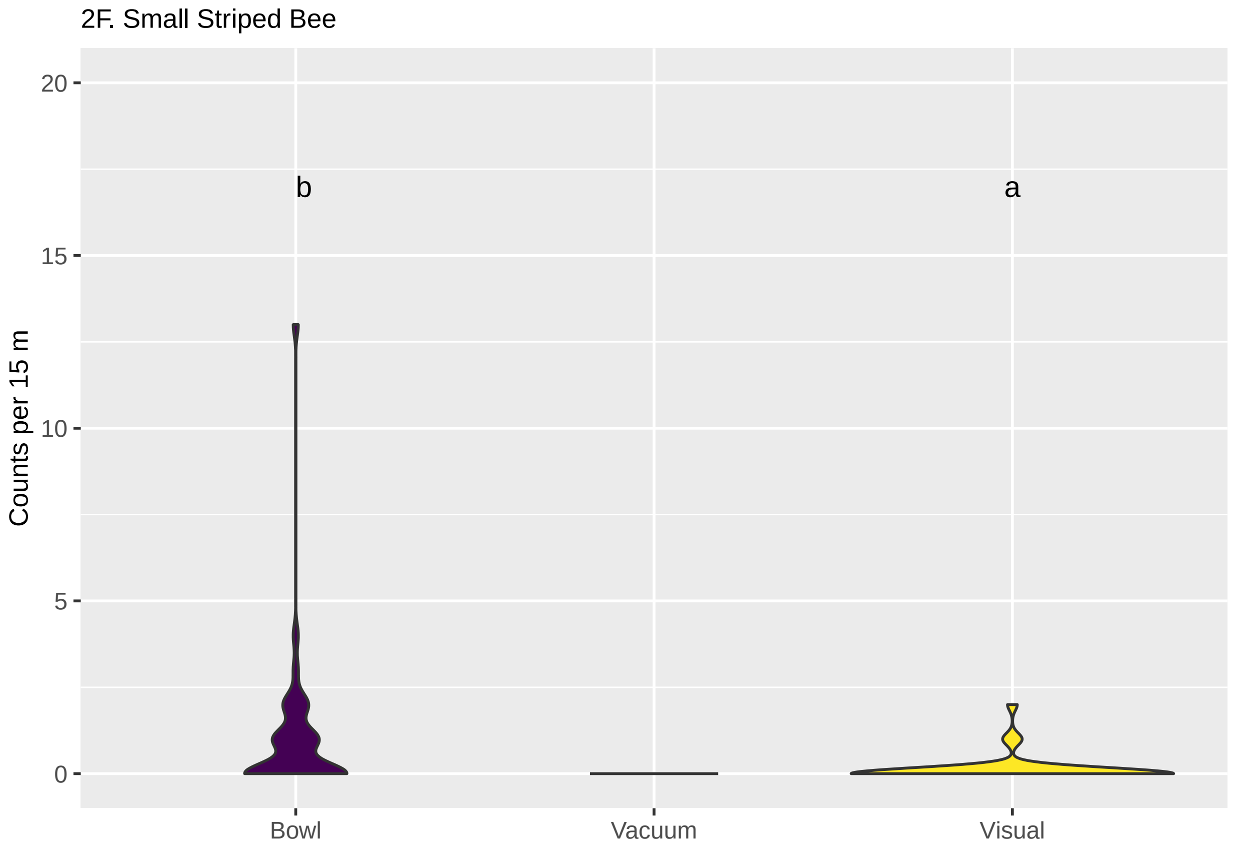

Green Bee

```
#checking for zero inflation
hist(Trap$GB)
```

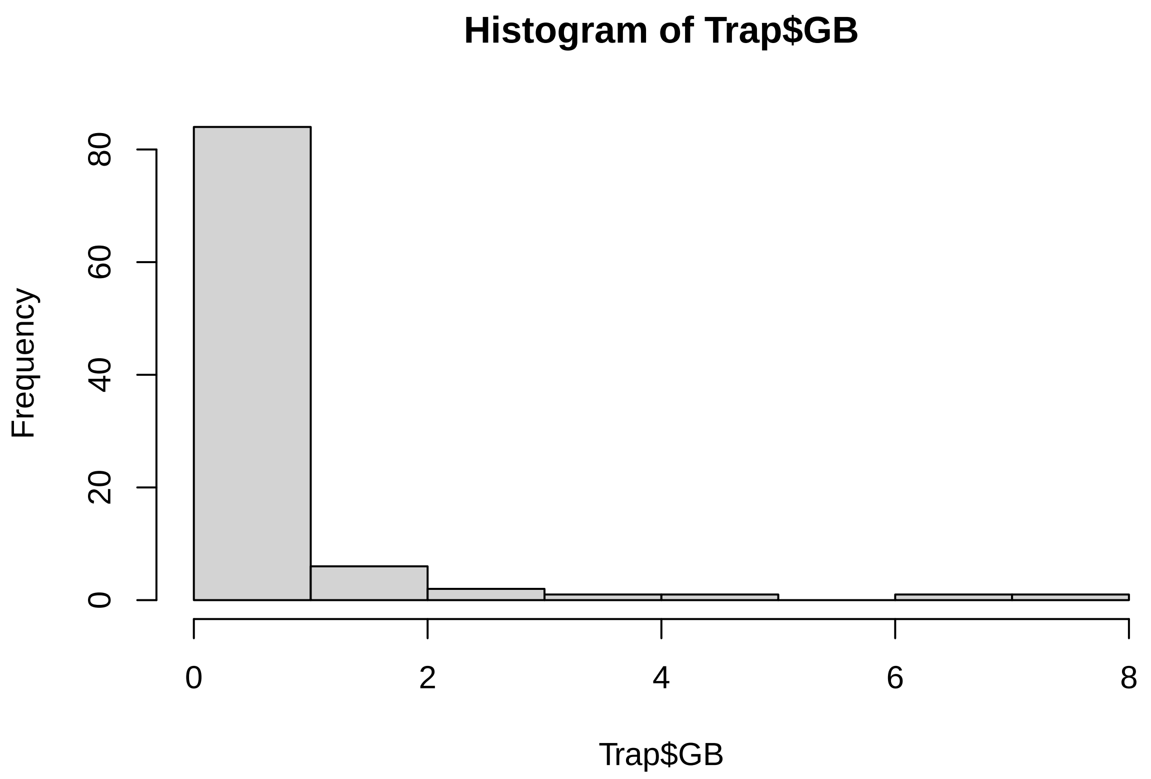


```
qqnorm(Trap$GB)
```

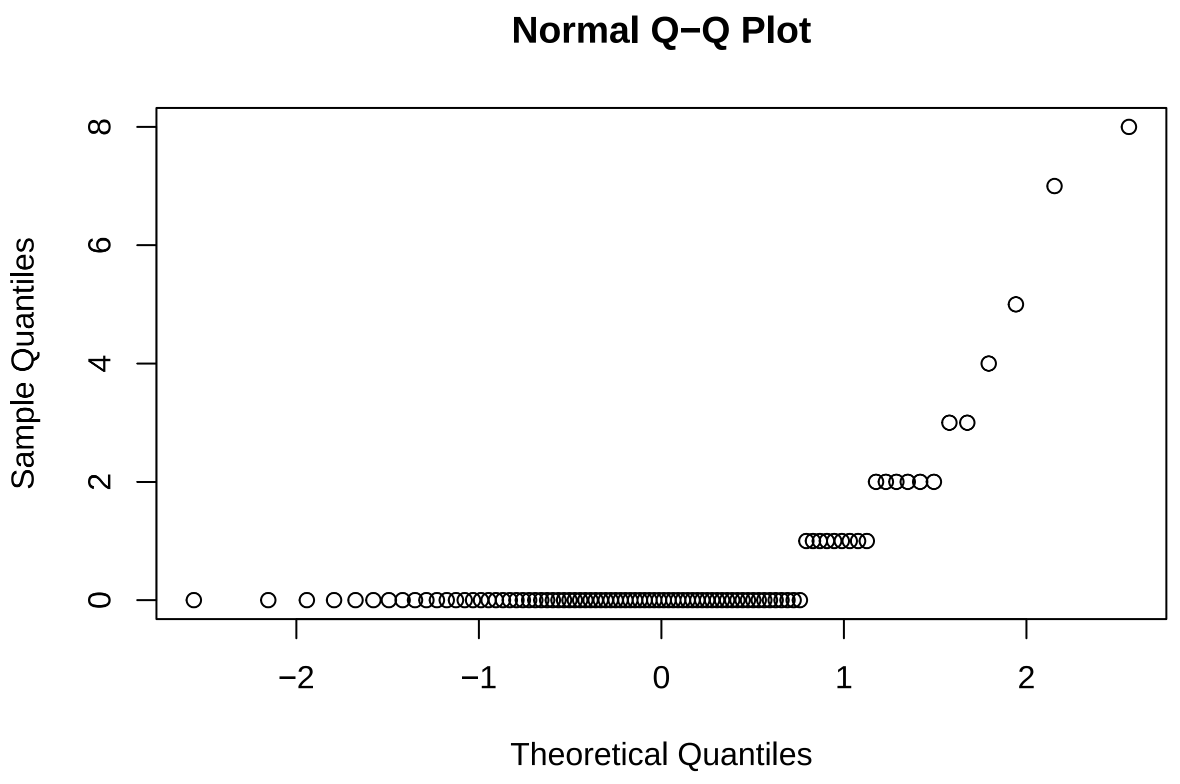


```r
#run zero inflated glmm
model.GB <- glmmTMB(GB ~ TRAP + (1 | LOC) + (1 | DATE),
                    ziformula = ~1,
                    family = poisson(),
                    data = Trap)

summary(model.GB)
```

```
##  Family: poisson  ( log )
## Formula:          GB ~ TRAP + (1 | LOC) + (1 | DATE)
## Zero inflation:      ~1
## Data: Trap
##
##      AIC       BIC    logLik -2*log(L)  df.resid
##    166.8     182.2     -77.4     154.8        90
##
## Random effects:
##
## Conditional model:
##  Groups Name        Variance Std.Dev.
##  LOC    (Intercept) 0.2945   0.5427
##  DATE   (Intercept) 0.3479   0.5898
## Number of obs: 96, groups:  LOC, 3; DATE, 8
##
## Conditional model:
##             Estimate Std. Error z value Pr(>|z|)
```

```
## (Intercept)  -1.1906     0.7745  -1.537  0.12423
## TRAPBowl      2.2737     0.8299   2.740  0.00615 **
## TRAPVAC      -1.6503     1.1263  -1.465  0.14287
## ---
## Signif. codes:  0 '***' 0.001 '**' 0.01 '*' 0.05 '.' 0.1 ' ' 1
##
## Zero-inflation model:
##             Estimate Std. Error z value Pr(>|z|)
## (Intercept)  0.02292    0.57946    0.04    0.968
```

```
#Runs the DHARMa residuals to test distrbutions and model fits
sim_res.GB <- simulateResiduals(fittedModel = model.GB, plot = TRUE)
```

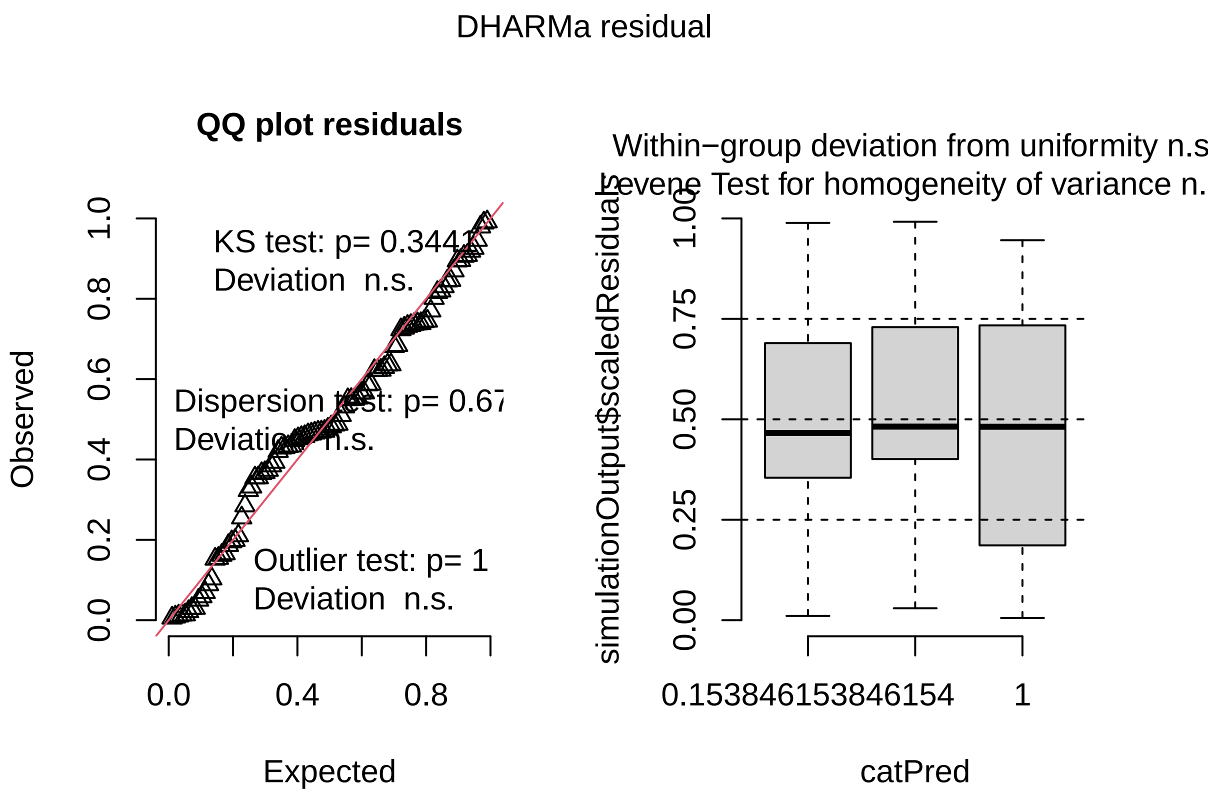


```
testResiduals(sim_res.GB)
```

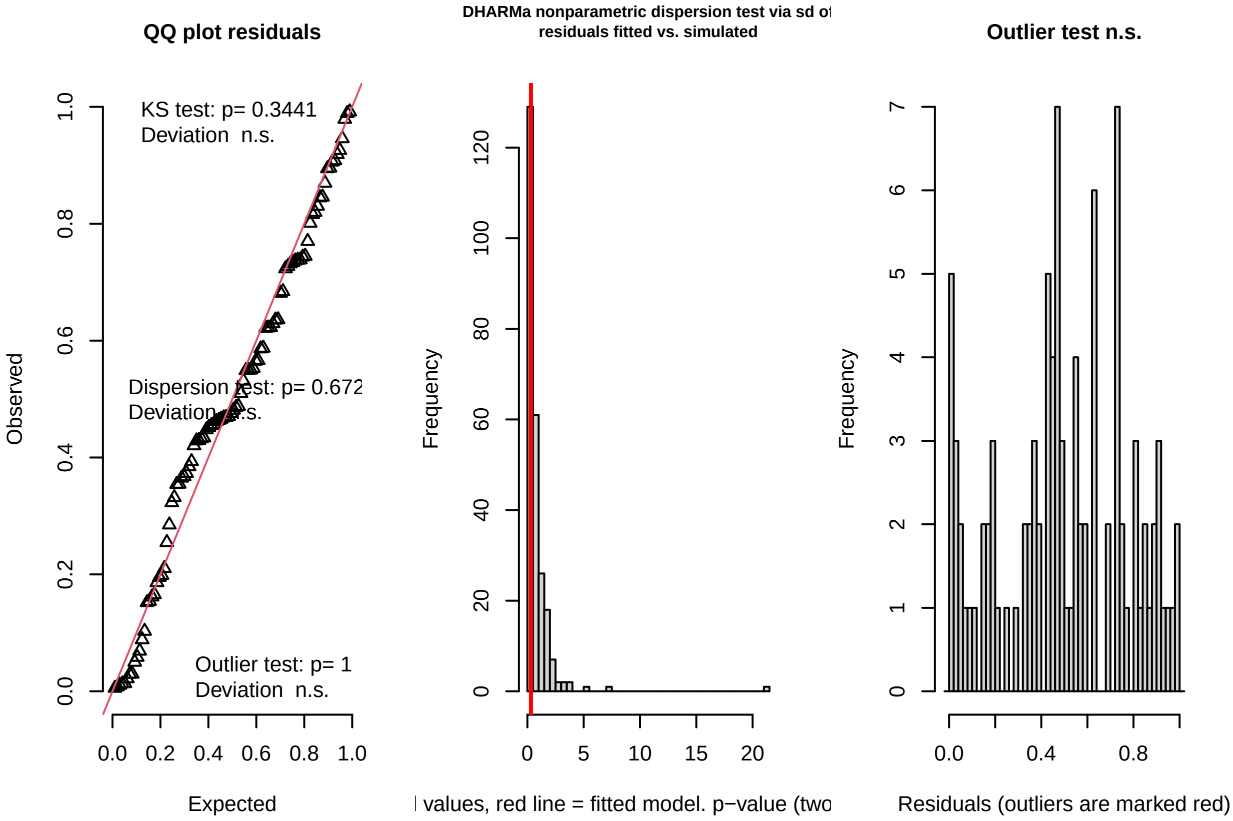


```
## $uniformity
##
##  Exact one-sample Kolmogorov-Smirnov test
##
## data:  simulationOutput$scaledResiduals
## D = 0.0939, p-value = 0.3441
## alternative hypothesis: two-sided
##
##
## $dispersion
##
##  DHARMa nonparametric dispersion test via sd of residuals fitted vs.
##  simulated
##
## data:  simulationOutput
## dispersion = 0.3819, p-value = 0.672
## alternative hypothesis: two.sided
##
##
## $outliers
##
##  DHARMa bootstrapped outlier test
##
## data:  simulationOutput
## outliers at both margin(s) = 0, observations = 96, p-value = 1
## alternative hypothesis: two.sided
```

```
##  percent confidence interval:
##  0.00000 0.03125
## sample estimates:
## outlier frequency (expected: 0.00479166666666667 )
##                                                   0
```

```r
testDispersion(sim_res.GB)
```

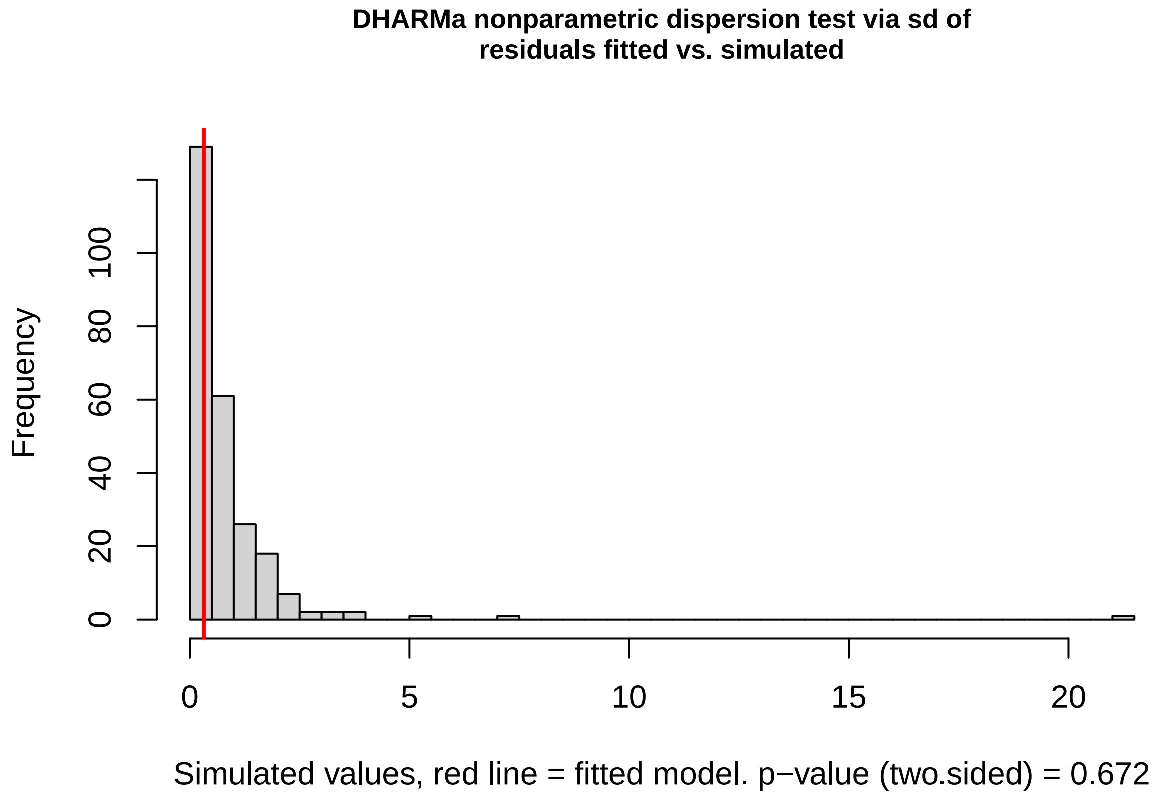


```
##
##  DHARMa nonparametric dispersion test via sd of residuals fitted vs.
##  simulated
##
## data:  simulationOutput
## dispersion = 0.3819, p-value = 0.672
## alternative hypothesis: two.sided
```

```r
testZeroInflation(sim_res.GB)
```

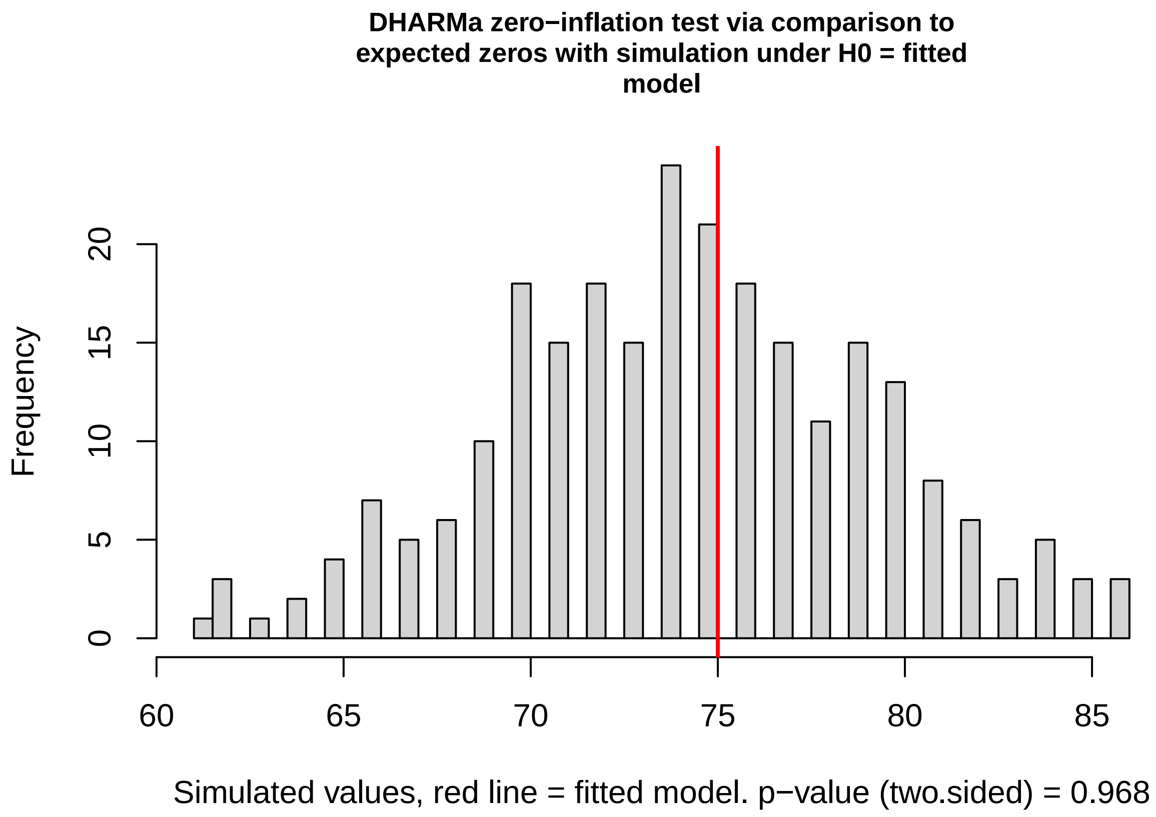


```
##
##  DHARMa zero-inflation test via comparison to expected zeros with
##  simulation under H0 = fitted model
##
## data:  simulationOutput
## ratioObsSim = 1.0087, p-value = 0.968
## alternative hypothesis: two.sided
```

```
# Reduced model for LRT
gb_reduced <- glmmTMB(GB ~ 1 + (1 | LOC) + (1 | DATE),
                    ziformula = ~1,
                    family = poisson(),
                    data = Trap)

# Likelihood ratio test
anova(model.GB,gb_reduced, test = "Chisq")
```

```
## Data: Trap
## Models:
## gb_reduced: GB ~ 1 + (1 | LOC) + (1 | DATE), zi=~1, disp=~1
## model.GB: GB ~ TRAP + (1 | LOC) + (1 | DATE), zi=~1, disp=~1
##            Df    AIC    BIC  logLik deviance  Chisq Chi Df Pr(>Chisq)
## gb_reduced  4 185.40 195.65 -88.698   177.40
## model.GB    6 166.78 182.16 -77.389   154.78 22.618      2  1.226e-05 ***
## ---
## Signif. codes:  0 '***' 0.001 '**' 0.01 '*' 0.05 '.' 0.1 ' ' 1
```

```r
#running emmeans with sidak
marginal.GB = emmeans(model.GB,
                   ~ TRAP, type = "response")
gb.cld <- cld(marginal.GB,
    alpha=0.05,
    Letters=letters,  ### Use lower-case letters for .group
    adjust="sidak")

# Clean up for plotting to add letters to plot
gb.cld_result <- as.data.frame(gb.cld)
gb.cld_result$group <- as.character(gb.cld_result$.group)

#PULL ONLY GB for graphing
GB.PLOT <- TRAP.1 %>% filter(MORPHO == 'GB')
Count.GB <- GB.PLOT %>% select_("TRAP", "MORPHO", "COUNT")
```

```
## Warning: `select_()` was deprecated in dplyr 0.7.0.
## i Please use `select()` instead.
## Call `lifecycle::last_lifecycle_warnings()` to see where this warning was
## generated.
```

```r
# GB Basic violin plot
p.gb <-ggplot(GB.PLOT, aes(x=TRAP, y=COUNT, fill=TRAP)) +
  geom_violin() + scale_x_discrete(labels = c("Bowl" = "Bowl",
                              "VAC " = "Vacuum",
                              "VIS " = "Visual"))  + labs(y = "Counts per 15 m")
p.gb.1 <- p.gb + theme(axis.title.x=element_blank(),axis.title.y = element_text(size = 10))
GB.final.plot <- p.gb.1 +
  scale_fill_viridis_d() + theme(legend.position = "none", plot.title = element_text(size = 10)) + ggti

GB.final.plot
```

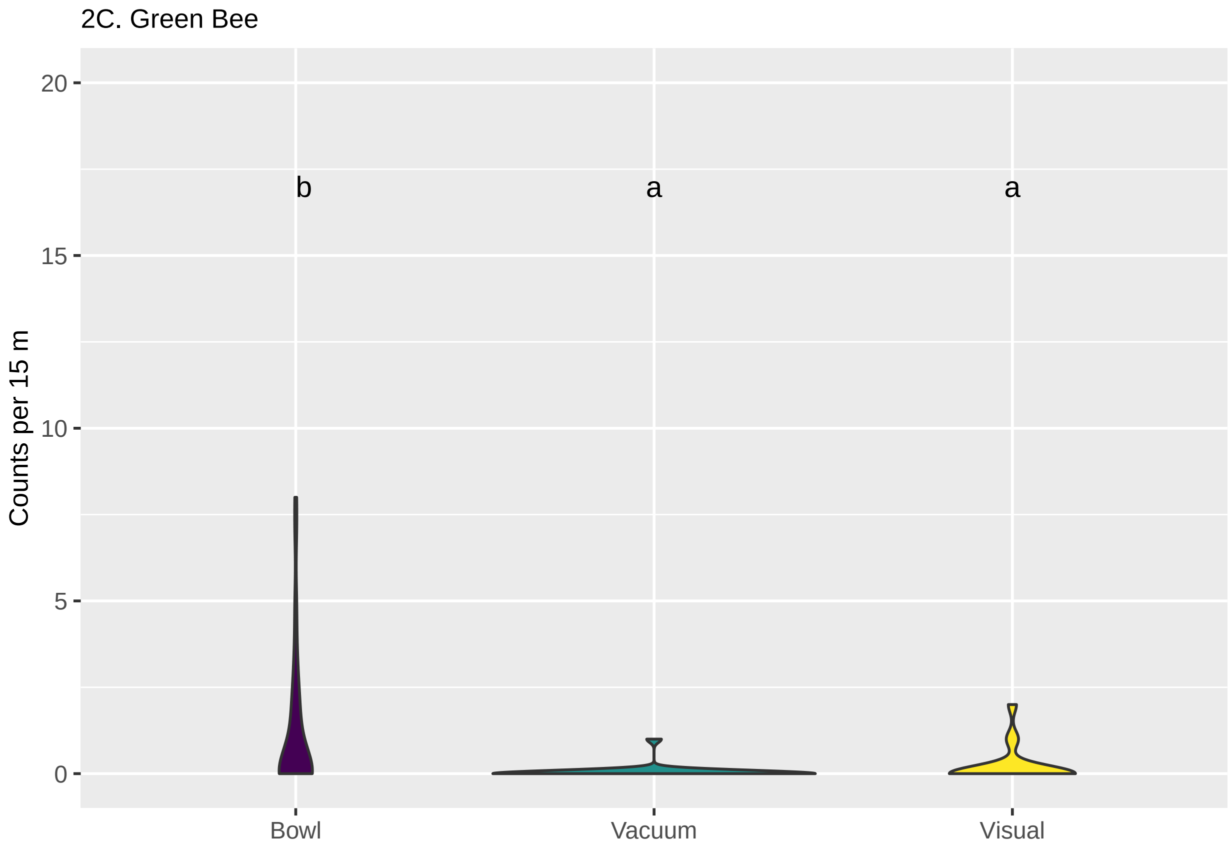

Make the final combine violin plot

```
# Create title and caption as grobs
title.fig2 <- textGrob("Figure 2. Violin plots of bees counts by plot of observation by Morphotaxa", gp

long.fig2 <- "Each violin plots represents counts of bees by observation event are on the y axis and th

# Wrap the text to ~80 characters wide (adjust to taste)
wrapped_caption.fig2 <- str_wrap(long.fig2, width = 120)
# Create the caption as a grob
caption <- textGrob(wrapped_caption.fig2, gp = gpar(fontsize = 8), x = .5, hjust = .5)


# Combine the top 6 plots into a 3-column layout
top_grid <- arrangeGrob(
  HB.final.plot, SBB.final.plot, GB.final.plot,
  BB.final.plot, LBB.final.plot, SSB.final.plot,
  ncol = 3
)

# Final full layout
fig2 <- grid.arrange(
  top_grid,                       # 3-column grid of plots
  SQB.final.plot,
  title.fig2,                     # Title
  caption,                        # Caption
  ncol = 1,
```

```
  heights = unit(c(20, 10, 1, 2), "null")  # Adjust heights as needed
)
```

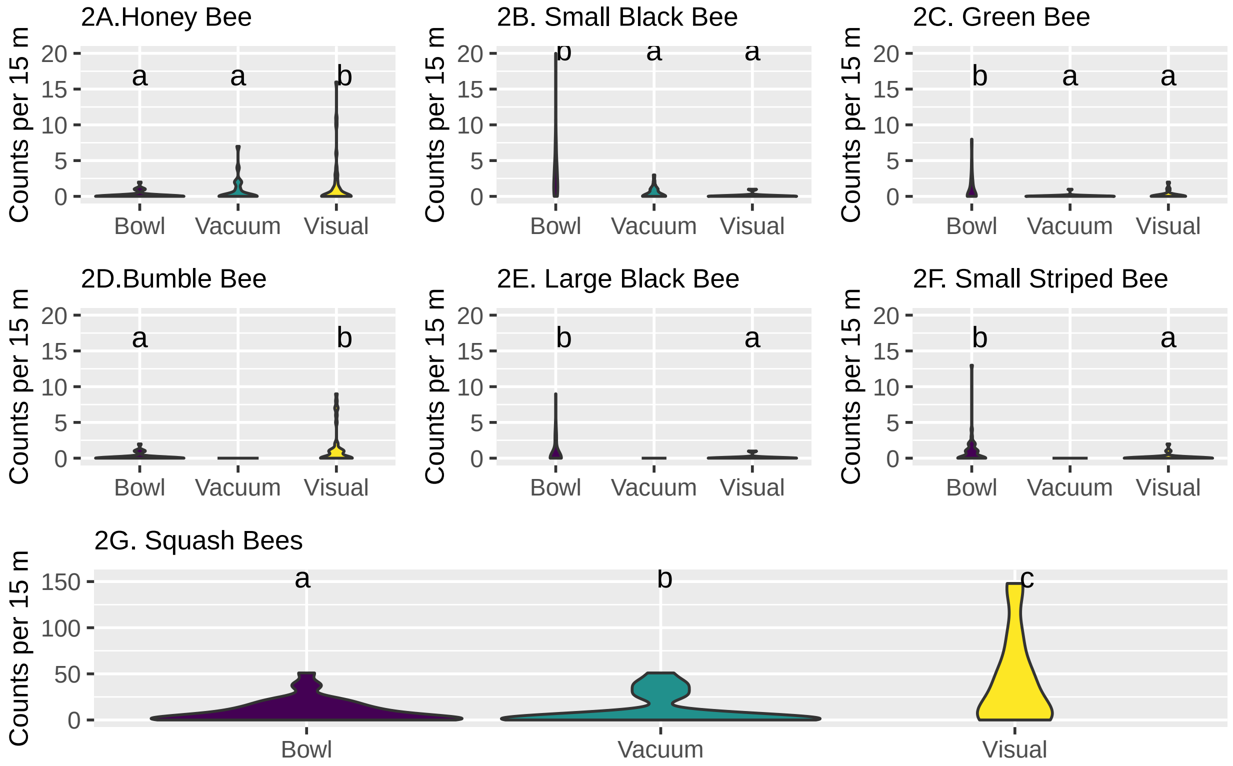

Figure 2. Violin plots of bees counts by plot of observation by Morphotaxa

Each violin plots represents counts of bees by observation event are on the y axis and the x axis is trap type for bowl, vacuum, or visual sampling. Violin plots have greater width at count values with higher frequencies of observation. For each morphotaxa, groups sharing the same cld letter are not significantly different based on zero−inflated general

```
ggsave(filename = "~/Desktop/trap.type.final/peerJ/Figs and tables for Peer J/figure2.all.png", plot = [illegible]

ggsave(filename = "~/Desktop/trap.type.final/peerJ/Figs and tables for Peer J/figure2.all.pdf", plot = [illegible]
```
