# Supplementary material for: Effects of sampling methods on bee pollinators observed in Cucurbita pepo
Source: PeerJ. 2026 Feb 2;14:e20649. doi: 10.7717/peerj.20649 (PMC12875216; doi:10.7717/peerj.20649)
Supplement: Supplemental Information 3 [file peerj-14-20649-s003.docx]

| Supplementary Table 1. Collected Bee Specimens From Trapping Methods  Collected bee voucher specimens identified by Sam Droege (USGS), T’ai Roulston, and Courtney Walls. Specimens were collected in *Cucurbita pepo* flowers or within the *Cucurbita pepo* field. Bees were separated by sex. Morphotaxa labels indicate the morphotaxa category given when bees were labeled in field. HB = Honey Bee, BB = Bumble Bee, SQB = Squash Bee, SBB = Small Black Bee, LBB =Large Black Bee, SSB = Small Striped Bee, LSB = Large Striped Bee, GB = Green Bee, and O = Other. This includes all samples collected from the bowl and vacuum sampling and bowl sampling methods. Visual sampling only includes representative samples. | | | | | | | | |
| --- | --- | --- | --- | --- | --- | --- | --- | --- |
| Family | Species | Sex | Morphotaxa | Bowl (yellow) | Bowl  (white) | Bowl  (blue) | Vacuum | Visual |
| Andrenidae | *Calliopsis andreniformis* Smith, 1853 | F | SSB | 4 | 10 | 4 |  |  |
|  |  | M | SBB | 2 | 10 | 10 |  |  |
| Apidae | *Apis mellifera* Linnaeus, 1758 | F | HB | 2 | 2 | 2 | 19 | 6 |
|  | *Bombus impatiens* Cresson, 1863 | F | BB |  | 3 | 3 |  | 3 |
|  | *Melissodes bimaculatus* Lepeletier, 1825 | F | LBB | 1 |  | 7 |  |  |
|  |  | M | LBB |  |  | 23 |  |  |
|  | *Triepeolus remigatus* Fabricius, 1804 | F | LSB | 3 | 1 |  |  |  |
|  |  | M | LSB |  | 2 |  |  | 1 |
|  | *Xenoglossa pruinosa* Say, 1837 | F | SQB | 19 |  | 119 | 240 | 2 |
|  |  | M | SQB | 26 | 3 | 182 |  |  |
|  | *Xenoglossa strenua* Cresson, 1878 | F | SQB |  |  | 1 | 1 |  |
| Halictidae | *Agapostemon texanus* Cresson, 1872 | F | GB |  |  | 1 |  |  |
|  | *Agapostemon virescens* Fabricius, 1775 | F | GB |  | 1 |  |  |  |
|  |  | M | GB |  | 1 |  |  |  |
|  | *Augochlora pura* Say, 1837 | F | GB |  |  |  | 1 | 1 |
|  | *Augochlorella aurata* Smith, 1853 | F | GB | 12 | 5 | 21 | 1 |  |
|  |  | M | GB | 1 | 1 |  |  |  |
|  | *Halictus ligatus/poeyi* Say, 1837/Lepeletier, 1841 | F | SSB | 2 | 4 | 5 |  |  |
|  |  | M | SSB | 1 |  | 2 |  |  |
|  | *Halictus parallelus* Say, 1837 | F | SSB | 3 | 1 | 1 |  |  |
|  | *Lasioglossum admirandum* Sandhouse, 1924 | F | SBB | 4 | 6 | 2 | 1 | 1 |
|  | *Lasioglossum albipenne* Robertson,1890 | F | SBB | 1 |  | 1 |  |  |
|  | *Lasioglossum bruneri* Crawford, 1902 | F | SBB |  |  | 1 |  |  |
|  | *Lasioglossum callidum* Sandhouse, 1924 | F | SBB | 5 | 3 | 4 |  | 2 |
|  | *Lasioglossum coreopsis* Robertson, 1902 | F | SBB |  |  | 1 |  |  |
|  | *Lasioglossum hitchensi* Gibbs, 2012 | F | SBB | 13 | 13 | 26 | 4 |  |
|  | *Lasioglossum imitatum* Smith, 1853 | F | SBB | 4 |  |  |  |  |
|  | *Lasioglossum leucozonium* Schrank, 1781 | M | SBB |  |  | 1 |  |  |
|  | *Lasioglossum lustrans* Cockerell, 1897 | F | SBB | 1 |  |  |  |  |
|  | *Lasioglossum pectorale* Smith, 1853 | F | SBB | 1 |  |  |  |  |
|  | *Lasioglossum pilosum* Smith, 1853 | F | SBB | 2 | 2 | 4 | 2 | 2 |
|  |  | M | SBB |  |  | 1 |  |  |
|  | *Lasioglossum pruinosum* Robertson, 1892 | F | SBB |  | 1 | 2 |  |  |
|  |  | M | SBB |  |  | 2 |  |  |
|  | *Lasioglossum spp* | F | SBB |  |  | 1 |  | 1 |
|  |  | M | SBB |  | 1 |  |  |  |
|  | *Lasioglossum tegulare* Robertson,1890 | F | SBB | 8 | 3 | 11 | 1 |  |
|  | *Lasioglossum trigeminum* Gibbs, 2011 | F | SBB | 3 | 2 | 4 |  |  |
|  | *Lasioglossum truncatum* Robertson, 1901 | F | SSB |  |  | 2 |  |  |
|  | *Lasioglossum versatum* Robertson, 1902 | F | SBB | 1 |  | 1 |  |  |
|  | *Lasioglossum weemsi* Mitchell, 1960 | F | SBB |  |  | 1 |  |  |
